# Supplementary material for: Analysis on personnel costs and working time for implementing a more person-centred care approach: a case study with embedded units in a Swedish region
Source: BMJ Open. 2023 Oct 11;13(10):e073829. doi: 10.1136/bmjopen-2023-073829 (PMC10582865; doi:10.1136/bmjopen-2023-073829)
Supplement: Supplementary data [file bmjopen-2023-073829supp001.pdf]

Study protocol, not to be referred to or cited 2020-03-12

Health economics of implementing person-centred care: the IMPROVE project

Suggested authors (order to be decided)

Hanna Gyllensten, Malin Tistad, Helena Fridberg, Eric Carlström, Lars Wallin

Background

Healthcare systems in many countries experiences increasing economic demands, both through the development of new technologies and treatments, and through a changing age distribution in the population resulting in more people with multiple chronic conditions.

Person-centred care (PCC) has been promoted both as a means to handle dissatisfaction with healthcare access and delivery,(1) and as potentially cost-saving or cost containing through more effective use of resources.(2) PCC acknowledges and endorses every person´s resources, interests, and needs, comprising shared responsibility and power, as well as a coordinated care and treatment.(3–5) It is related to the so called integrated people-centred health services promoted by the World Health Organization(6) albeit without the community perspective included in that framework.

However, PCC it is often described as a complex intervention; an intervention containing several interacting components,(7,8) through including elements tailored to each participant, activities achieved in different ways, and with multiple and sometimes varying outcomes and goals for different participants. Thus, research into its use and effects needs to handle the complexity involved,(7) including a thorough understanding of how the intervention is produced through e.g., process evaluation of the intervention created.(9) In a conceptual model for such implementation research, Procter and colleagues elaborated on the outcomes evaluation, including intervention outcomes, service outcomes and client outcomes.(10)

This study is, as far as we know one of few measuring the cost of implementing person-centred care. Several studies suggests cost-saving as one reason to implement person-centred care,(2,11) but the cost to implement the method is seldom mentioned. Even though, person-centred care has an extensive impact on the heath-care industry. In Sweden legislators and health care providers has introduced person-centred care on the agenda of development and management-control efforts is more and more about implementing person-centred care. In this study we measure the cost of such an implementation.

Aims

The aims of this project was to map the resources used in implementing PCC, in the healthcare unit as well as on a regional level. A secondary aim was to explore how this implementation affected the budget, from the healthcare provider's perspective.

Ursprunglig formulering:

- *Att kartlägga resursförbrukning för att införa PCV på lokal nivå respektive på regional/landstingsnivå.*
- *Att undersöka hur landstingets budget påverkas av att införa PCV.*

Methods

Contextual factors

Region Dalarna decided in 2016 to promote a more person-centred care approach throughout the public healthcare system, as part of their work towards efficient healthcare practices (in parallel with other ongoing projects within the so called “Struktur- och förändringsarbetet”, including projects related to priority setting and resource allocation in local health systems). As part of this implementation process, changes were made on micro, meso, and macro level in the region. The *Implementing person centred care: Process evaluation of strategies, leadership and health economy* (IMPROVE) project was established to follow this implementation process. In the project, six healthcare units were followed to explore the strategies used to implement a more person-centred care and the associated outcomes, as well as the policies and leadership related aspects of this process. Data was collected through logbooks, questionnaires, interviews and review of medical records, to give a comprehensive overview of how the process evolved over time.

The implementation process studied in the IMPROVE project was introduced and implemented by Region Dalarna. The process evaluation was conducted in parallel by researchers from collaborating universities partnering with staff in the healthcare units.

Data collection

Six healthcare units were included in the process evaluation, selected based on being early adapters to the implementation of PCC. Included healthcare units were followed from six months before to 18 months after the introduction of PCC. The units included were specialized in nephrology, geriatric rehabilitation, psychiatry and primary care, respectively.

Staff involved in introducing PCC were asked to complete logbooks of their activities and hours spent. Information from the logbooks was thus used to identify both which activities were perceived by personnel in different segments of the healthcare organization to be related to implementing PCC, and to estimate the time used for this implementation. Logbooks were also collected from operational support personnel involved in supporting care units during the implementation. Costs for used resources was calculated based on the time used for each type of activity (categorized inductively after data collection) times the average cost per hour for the relevant staff category, retrieved from the healthcare provider.

In addition, included units were asked to provide their own suggestions for evaluating the economic impact of PCC on their practices. Relevant data was collected from each unit retrospectively, and the units were asked to comment on any potential time trends visible in the data.

A third data source was administrative data collected from the region, including economic results, patient quantities, mean length of admission, and hours worked. This information was used to provide a background to the data collected from logbooks and included healthcare units.

Analyses

Descriptive statistics of time used and associated costs, as reported in the logbooks, will be reported by types of activities associated with the implementation of PCC. A bootstrap methodology will be used to calculate 95% confidence intervals, to account for expected skewed distributions of costs.

The unit specific outcomes will be analyzed longitudinally and described separately for each unit. Unit specific measures included:

Nephrology/dialysis unit	Distribution between haemodalysis, assisted peritoneal dialysis and unassisted peritoneal dialysis

	Work hours (adjusted for number of patients)
Primary care	Number of patients listed<br>Number of visits per patient
Geriatric rehabilitation	Unit 1: Length of stay<br>Unit 2: Work hours, number of patients, work environment follow-up
Psychiatrics	Unit 1: Length of stay (taking overcrowding into consideration)<br>Unit 2: Length of stay and readmissions (taking overcrowding into consideration)<br>These two units were combined during a part of the study, this will also need to be considered.

Regional administrative data will be reported descriptively for each year the study was ongoing.

Suggested tables and figures

Table 1: Overview of respondents to the logbooks, including an overview of the activities reported

Table 2: Hours and associated costs for each type of activity reported in the logbooks

Table 3: Results for the unit specific measures [*may become divided into one per unit or so*]

References

1. Sharma T, Bamford M, Dodman D. Person-centred care: an overview of reviews. ContempNurse. 2015;51(2–3):107–20.

2. Hansson E, Ekman I, Swedberg K, Wolf A, Dudas K, Ehlers L, et al. Person-centred care for patients with chronic heart failure - a cost-utility analysis. EurJCardiovascNurs. 2015;(Journal Article).

3. Mead N, Bower P. Patient-centredness: a conceptual framework and review of the empirical literature. SocSciMed. 2000;51(7):1087–110.

4. Ekman I, Swedberg K, Taft C, Lindseth A, Norberg A, Brink E, et al. Person-centered care--ready for prime time. EurJCardiovascNurs. 2011;10(4):248–51.

5. Harding E, Wait S, Scrutton J. The state of play in person-centred care: A pragmatic review of how person-centred care is defined, applied and measured [Internet]. The Health Policy Partnership Ltd; 2015 p. 1–140. Available from: http://www.healthpolicypartnership.com/person-centred-care/

6. World Health Organization. Framework on integrated people-centred health services. WHO; 2016 p. 1–12.

7. Craig P, Dieppe P, Macintyre M, Michie S, Nazareth I, Petticrew M. Developing and evaluating complex interventions: new guidance [Internet]. Medical Research Council; 2006 p. 1–39. Available from: https://www.mrc.ac.uk/documents/pdf/complex-interventions-guidance/

8. Craig P, Dieppe P, Macintyre S, Michie S, Nazareth I, Petticrew M, et al. Developing and evaluating complex interventions: the new Medical Research Council guidance. BMJ. 2008;337(Journal Article):a1655.

9. Moore GF, Audrey S, Barker M, Bond L, Bonell C, Hardeman W, et al. Process evaluation of complex interventions: Medical Research Council guidance. BMJ. 2015;350(Journal Article):h1258.

10. Proctor EK, Landsverk J, Aarons G, Chambers D, Glisson C, Mittman B. Implementation research in mental health services: an emerging science with conceptual, methodological, and training challenges. AdmPolicy MentHealth. 2009;36(1):24–34.

11. Sahlen KG, Boman K, Brännstrom M. A cost-effectiveness study of person-centered integrated heart failure and palliative home care: Based on a randomized controlled trial. PalliatMed. 2016;30(3):296–302.
