# Supplementary material for: Analysis on personnel costs and working time for implementing a more person-centred care approach: a case study with embedded units in a Swedish region
Source: BMJ Open. 2023 Oct 11;13(10):e073829. doi: 10.1136/bmjopen-2023-073829 (PMC10582865; doi:10.1136/bmjopen-2023-073829)
Supplement: Supplementary data [file bmjopen-2023-073829supp003.pdf]

Online-Only Supplements

An analysis on personnel costs and working time for implementing a more person-centred care approach: A case study with embedded units in a Swedish region

Authors

Hanna Gyllensten[1,2] Associate professor

Malin Tistad[3,4] Associate professor

Helena Fridberg[3] PhD

Lars Wallin[1,2,3] Professor

Author affiliations

[1] Institute of Health and Care Sciences, University of Gothenburg, Box 457, SE-405 30 Gothenburg, Sweden.

[2] University of Gothenburg Centre for Person-Centred Care (GPCC), Sahlgrenska Academy, University of Gothenburg, Sweden.

[3] School of Health and Welfare, Dalarna University, Falun, Sweden.

[4] Department of Neurobiology, Care Sciences and Society, Karolinska Institutet, Stockholm, Sweden.

Content

eTable 1: Resource use reported in logbooks by type of activity and organisational units. 2

eFigure 1: Length of stay at the geriatric unit (unit 1) and trend over time. 3

eFigure 2: Length of stay at the psychiatric units (combined) and trend over time. 4

eFigure 3: Time to readmission at the psychiatric units (combined) and trend over time. 5

eTable 1: Resource use reported in logbooks by type of activity and organisational units.

Activities by cost categories[a]	Organisational unit							
	Geriatric unit 1 working days[b]	Geriatric unit 2 working days[b]	Nephrology unit working days[b]	Primary care unit working days[b]	Psychiatric unit 1 working days[b]	Psychiatric unit 2 working days[b]	DD working days[b]	Total working days[b]
Implementation strategies								
Documentation	0	0	0	1	0	0	0	1
Educational activities	0	0	0	0	0	0	46	46
External communication	0	0	0	0	0	0	1	1
Internal communication	0	0	0	0	0	0	8	8
Learning seminars	18	25	57	42	15	19	92	268
Preparatory work	1	70	11	2	0	0	88	171
Workplace meetings	0	0	1	0	0	0	0	1
Service delivery								
Administration	0	0	0	0	0	0	2	2
Care development	113	41	32	35	53	74	0	348
Documentation	0	0	0	1	0	0	0	1
Educational activities	26	4	37	105	0	0	0	172
IT support	0	0	0	0	0	0	0	0
Reporting	0	0	0	0	0	0	4	4
Team development	23	22	5	0	183	0	0	233
Workplace meetings	0	0	0	71	0	0	0	71
Research/development								
External communication	0	0	11	11	10	0	15	48
Preparatory work	0	0	0	1	0	0	0	1
Research	5	4	9	7	2	2	13	42
Total	185	167	162	275	263	95	267	1414

[a] Developed from previously reported cost categories in implementation programmes: 1) costs for executing implementation strategies, 2) excess costs for service delivery as implementation/service changes, 3) opportunity costs to providers and patients, and 4) research/development costs (35). No account was taken for changes in costs for direct patient care (category 3).

[b] Assuming 8-hour working days.

Abbreviations: DD = Department for development.

Gyllensten H, *et al. BMJ Open* 2024; 13:e073829. doi: 10.1136/bmjopen-2023-073829

eFigure 1: Length of stay at the geriatric unit (unit 1) and trend over time.

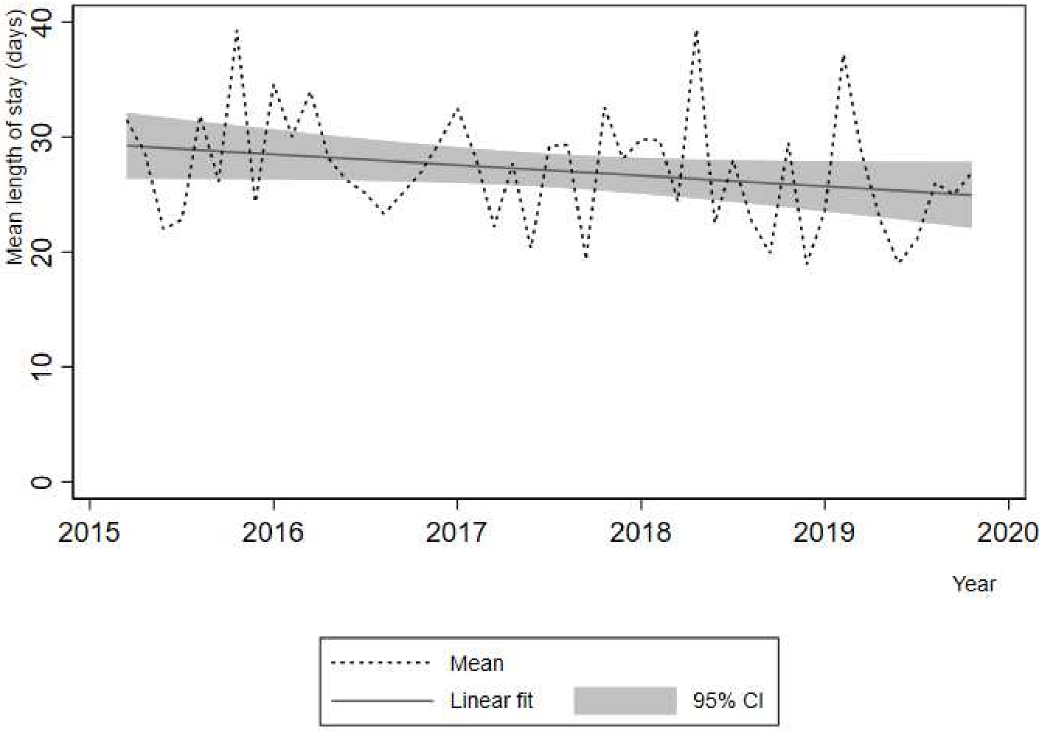

eFigure 2: Length of stay at the psychiatric units (combined) and trend over time.

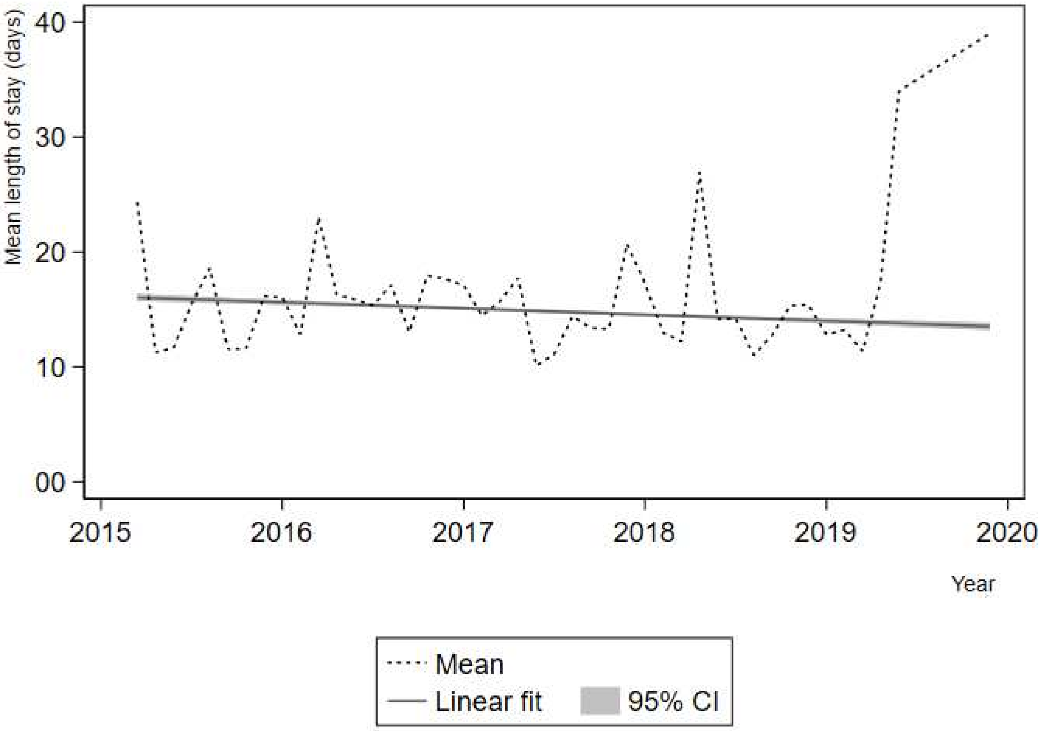

Observe that the 95% confidence interval is not visible (overlaid by the linear equation line) due to large sample size.

eFigure 3: Time to readmission at the psychiatric units (combined) and trend over time.

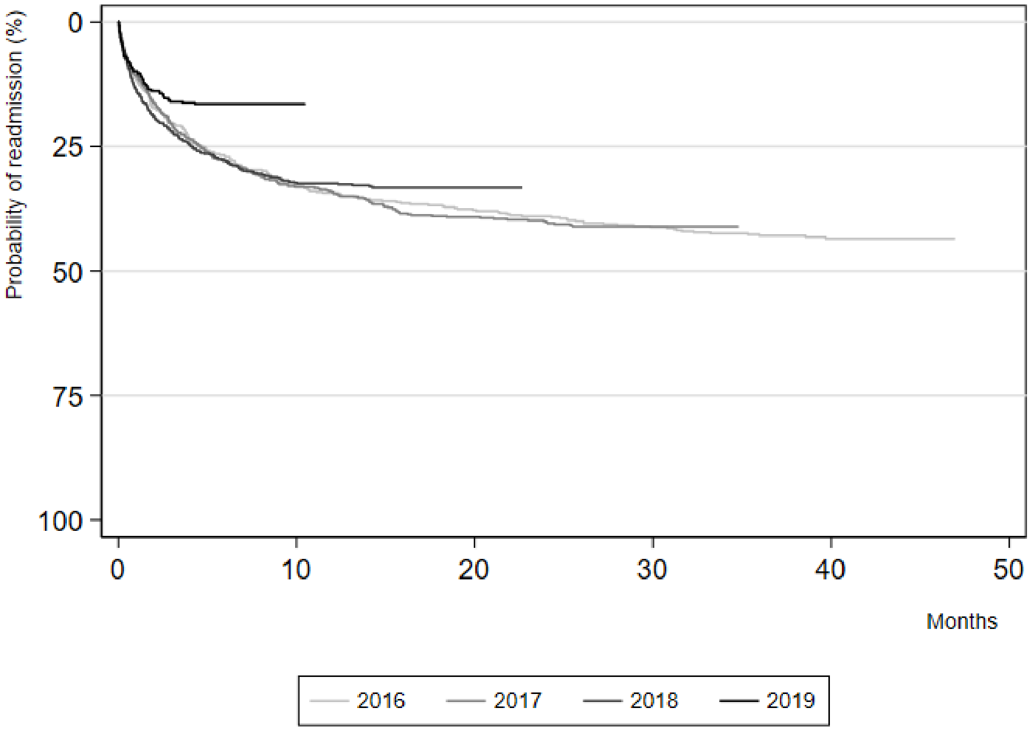

Gyllensten H, *et al. BMJ Open* 2024; 13:e073829. doi: 10.1136/bmjopen-2023-073829
