# Supplementary material for: Analysis on personnel costs and working time for implementing a more person-centred care approach: a case study with embedded units in a Swedish region
Source: BMJ Open. 2023 Oct 11;13(10):e073829. doi: 10.1136/bmjopen-2023-073829 (PMC10582865; doi:10.1136/bmjopen-2023-073829)
Supplement: Author's manuscript [file bmjopen-2023-073829.draft_revisions.pdf]

BMJ Open is committed to open peer review. As part of this commitment we make the peer review history of every article we publish publicly available.

When an article is published we post the peer reviewers' comments and the authors' responses online. We also post the versions of the paper that were used during peer review. These are the versions that the peer review comments apply to.

The versions of the paper that follow are the versions that were submitted during the peer review process. They are not the versions of record or the final published versions. They should not be cited or distributed as the published version of this manuscript.

BMJ Open is an open access journal and the full, final, typeset and author-corrected version of record of the manuscript is available on our site with no access controls, subscription charges or pay-per-view fees (http://bmjopen.bmj.com).

If you have any questions on BMJ Open's open peer review process please email info.bmjopen@bmj.com

BMJ Open

Health economics of implementing a more person-centred care approach: the IMPROVE project

Journal:	*BMJ Open*
Manuscript ID	bmjopen-2023-073829
Article Type:	Original research
Date Submitted by the Author:	22-Mar-2023

Complete List of Authors:	Gyllensten, Hanna; University of Gothenburg, Institute of Health and Care Sciences; University of Gothenburg Centre for Person-Centred Care<br>Tistad, Malin; Dalarna University, School of Health and Welfare; Karolinska Institute, Department of Neurobiology, Care Sciences and Society<br>Fridberg, Helena; Dalarna University, School of Health and Welfare<br>Wallin, Lars; Dalarna University, School of health and welfare; University of Gothenburg, Institute of Health and Care Sciences

Keywords:	HEALTH ECONOMICS, Health economics < HEALTH SERVICES ADMINISTRATION & MANAGEMENT, Organisational development < HEALTH SERVICES ADMINISTRATION & MANAGEMENT, Patient-Centered Care

SCHOLARONE™
Manuscripts

I, the Submitting Author has the right to grant and does grant on behalf of all authors of the Work (as defined in the below author licence), an exclusive licence and/or a non-exclusive licence for contributions from authors who are: i) UK Crown employees; ii) where BMJ has agreed a CC-BY licence shall apply, and/or iii) in accordance with the terms applicable for US Federal Government officers or employees acting as part of their official duties; on a worldwide, perpetual, irrevocable, royalty-free basis to BMJ Publishing Group Ltd ("BMJ") its licensees and where the relevant Journal is co-owned by BMJ to the co-owners of the Journal, to publish the Work in this journal and any other BMJ products and to exploit all rights, as set out in our licence.

The Submitting Author accepts and understands that any supply made under these terms is made by BMJ to the Submitting Author unless you are acting as an employee on behalf of your employer or a postgraduate student of an affiliated institution which is paying any applicable article publishing charge ("APC") for Open Access articles. Where the Submitting Author wishes to make the Work available on an Open Access basis (and intends to pay the relevant APC), the terms of reuse of such Open Access shall be governed by a Creative Commons licence – details of these licences and which Creative Commons licence will apply to this Work are set out in our licence referred to above.

Other than as permitted in any relevant BMJ Author's Self Archiving Policies, I confirm this Work has not been accepted for publication elsewhere, is not being considered for publication elsewhere and does not duplicate material already published. I confirm all authors consent to publication of this Work and authorise the granting of this licence.

For peer review only

Health economics of implementing a more person-centred care approach: the IMPROVE project

Authors

Hanna Gyllensten[1,2] Associate professor

Malin Tistad[3,4] Associate professor

Helena Fridberg[3] PhD

Lars Wallin[1,2,3] Professor

Author affiliations

[1] Institute of Health and Care Sciences, University of Gothenburg, Box 457, SE-405 30 Gothenburg, Sweden.

[2] University of Gothenburg Centre for Person-Centred Care (GPCC), Sahlgrenska Academy, University of Gothenburg, Sweden.

[3] School of Health and Welfare, Dalarna University, Falun, Sweden.

[4] Department of Neurobiology, Care Sciences and Society, Karolinska Institutet, Stockholm, Sweden.

Corresponding author

Hanna Gyllensten, Institute of Health and Care Sciences, Sahlgrenska Academy, University of Gothenburg, Postal Address: Box 457, SE-405 30 Gothenburg, Sweden, E-mail: hanna.gyllensten@gu.se. Phone: +46-(0)70-748 24 12

Key words

Person-centred care; Person-centered care; Costs and cost analysis; Implementation science.

Word count (excluding title page, abstract, references, figures and tables): 4686

For peer review only - http://bmjopen.bmj.com/site/about/guidelines.xhtml

For peer review only

For peer review only - http://bmjopen.bmj.com/site/about/guidelines.xhtml

Abstract

Objectives: Our aim was to describe the time and costs used during the implementation of a more person-centred care (PCC) approach as part of ordinary practice.

Design: A case study with embedded units.

Setting: Region Dalarna, Sweden.

Participants: The Department for Development (DD) staff who provided a central support function in the implementation and six healthcare units: nephrology, two geriatric care and rehabilitation units, two psychiatry units, and primary care.

Interventions: More PCC.

Primary and secondary outcome measures: Working days and related salary costs reported by categories indicating costs for implementation strategies, service delivery, and research/development costs.

Results: The health care units logged on average 5.5 working days per staff member. In the healthcare units, 5-57% of the time reported was used for implementation strategies, 40-90% for service delivery, and 3-12% for research/development. Of the time reported by the DD, 88% was assigned to implementation strategies. Costs associated with reported time indicated 23% of costs for this implementation occurred in the DD. Using the budgeted cost, this proportion increased to 48%. The budget for the DD corresponded to SEK 2.30 per citizen per year and 0.009 % of the total health care budget of the region.

Conclusions: The study found that a large part of resources used for this implementation of more PCC occurred in the DD, although at least half of the costs occurred in the healthcare units. Moreover, the cost of providing a central support function corresponds to a tiny proportion of the total health budget.

For peer review only - http://bmjopen.bmj.com/site/about/guidelines.xhtml

Strengths and limitations of this study

- The study was conducted by an independent research body that included researchers from different disciplines.
- The included healthcare units were given multiple opportunities to clarify and correct their records.
- The staff had no time set off for the logbooks and thus often filled in the logbooks retroactively, potentially resulting in recall bias.
- Most of the observed changes were due to other factors than the implementation of a more PCC, due to parallel changes in these units.

For peer review only

For peer review only - http://bmjopen.bmj.com/site/about/guidelines.xhtml

Introduction

Healthcare systems in many countries experience increasing economic demands, both through the development of new technologies and treatments and through a changing age distribution in the population resulting in more people with multiple chronic conditions. As in many countries (1,2), legislators and health care organisations in Sweden have put person-centred care (PCC) high on the development agenda (3), and management-control efforts are increasingly aimed at implementing PCC.

PCC acknowledges and endorses every person´s resources, interests, and needs, comprising shared responsibility and power, as well as coordinated care and treatment (4–6). PCC strives towards a meaningful life, which differs from similar concepts (e.g., patient-centred care) that focus on functional life (7). It is related to the integrated people-centred health services promoted by the World Health Organisation (8), albeit without the community perspective embedded in the framework. PCC has been promoted to address patient dissatisfaction with health care access and delivery (9) and as a potentially cost-saving or cost containing measure through more effective use of resources (10). Several studies suggest cost-saving as one argument for PCC (10,11). Thus, PCC is expected to both improve care quality and contain costs (12).

However, the knowledge about costs associated with introducing a more PCC is limited and scattered (13). Some studies have included training costs in intervention costs (14,15) or indicated costs of transferring staff between organizational units (16), but to our knowledge its implementation costs have not been reported. Regardless of the little knowledge of costs associated with its implementation, PCC has a growing impact on the health care industry in many countries (1,2). Overlooking such costs, however, implies that the time added for this implementation is minimal and can be ignored (17), an assumption that to some extent contradicts previous findings that implementation of PCC was associated with increased job strain (18).

For peer review only - http://bmjopen.bmj.com/site/about/guidelines.xhtml

Thus, this study aimed to describe the time and costs used during the implementation of a more PCC approach as part of ordinary practice. As far as we know, the current study is one of a few reporting health economic aspects of a more PCC approach in ordinary practice, i.e., not as part of an intervention study.

Materials and Methods

Region Dalarna decided in 2015 to promote a more PCC approach throughout the health system. The implementation process was initiated and managed by the healthcare organisation Region Dalarna as an essential part of continuous quality improvement. The *Implementing PCC: Process evaluation of strategies, leadership, and health economy* (IMPROVE) project was conducted in parallel by university researchers. The process evaluation focused on the implementation process rather than PCC (the innovation being implemented). This study thus reports on the resources used for implementing a more PCC approach in one Swedish region, using data from a case study with seven embedded units (*study protocol available as Supporting information*). Previous publications from the IMPROVE project have investigated how patients' perception of person-centred care can be measured (19), how the concept of person-centred care was perceived by health care staff (20), how the concept of PCC was operationalized by the units (21), the used implementation strategies (22), and congruence of managers' perceptions and understanding of PCC across organisational levels (23).

Setting

Region Dalarna is situated in the middle of Sweden. The region covers 6% of the Swedish land area and contains 3% of its population. Four hospitals and 25 healthcare clinics provides public healthcare for the region's population (24). The Swedish health system provides universal coverage for all legal residents, and on some premises, also to visitors from other parts of the European Union, asylum seekers, and undocumented immigrants. The health system is divided based on geographic area into 21 regions providing health care services and 290 municipalities providing care for the elderly and

For peer review only

disabled. Approximately 84% of all health expenditures were tax-based in 2016, with care prioritised based on an ethical platform including the principles of human dignity, needs and solidarity, and cost-effectiveness, in that order (25).

The innovation

The implementation of more PCC was conducted as part of the region's work towards efficient health care practices, in parallel with their "structure and change work" that included projects related to priority setting and resource allocation in the regional healthcare system. The vision was to put equal emphasis on the patient and professional perspective throughout the care process. The approach chosen was based on the Gothenburg model of PCC (5,26), which has been shown in clinical trials to be cost-effective for several care settings and patient groups (10,27–29). The main feature of the model is its focus on the partnership between the patient and the health care provider built during the co-creation of a written health plan (5). It has previously been reported that, although some ambiguity remained in their description, core practices related to all three cornerstones of PCC – creating, safeguarding and documenting the partnership – were identified in all the embedded units (20). Important practices introduced in the units were routines to elucidate the patient narrative during admission or throughout the care pathway, and use of communication techniques such as motivational interviewing.

For peer review only

The implementation process

As part of the implementation process, changes were made to the region's micro, meso, and macro levels. These changes included the commission of 80% of full-time staff in the Department for Development engaged as operational support provided centrally in the region (hereafter called the DD), as well as a budget for expenses associated with the implementation. One of the DD tasks was to organise learning seminars and support the staff at the health care units during the implementation process. The participating health care units chose their representatives to participate in the learning seminars. Thereafter, each health care unit was expected to manage its implementation process. There was no joint implementation support programme other than the

For peer review only - http://bmjopen.bmj.com/site/about/guidelines.xhtml

learning seminars, but the DD could provide support on-demand. The implementation strategies used have previously been described to mainly fall in two clusters, i.e., Train and educate stakeholder, as well as Develop stakeholder interrelationships (22).

The process evaluation

Interventions or programmes directed to changing health care provision, such as the implementation of PCC, are often described as complex interventions (30). Complex interventions are interventions that contain several interacting components. This interaction can include elements tailored to each participant (e.g., patients or health care staff), sometimes with varying outcomes and goals. In this study complexity included differences in the training of participating staff, differences in organisational structures among units, and each unit defining on their own how an increase in PCC would be interpreted and implemented. It has been recommended that research into the use and effects of complex interventions address the complexity involved (31). This can be done through process evaluations that provide an understanding of how the innovation (in this case PCC) is implemented (32). In a conceptual model for implementation research Proctor and colleagues elaborated on a number of implementation outcomes relevant for such an evaluation, including economic aspects of implementation (33).

The process evaluation was conducted six health care units that participated in the first round of learning seminars and the DD. All units consented to participate in the evaluation. The health care units included were specialised in nephrology, geriatric care and rehabilitation, psychiatry, and primary care.

Costs related to the implementation included staff and the DD's costs, the training of staff, and any support provided locally in the health care units. The study examines resources spent on making this change in health care but does not include any control units. Therefore, the study approach is in line with an observational study of a natural experiment in a real-world setting; i.e., the research team

For peer review only - http://bmjopen.bmj.com/site/about/guidelines.xhtml

has no control over the intervention, there is no control condition, and the knowledge and availability of data for evaluation are partial (34).

Data collection

Data for this study were collected through logbooks, including date and type of activity, how much time each activity took (recorded as either minutes, hours, or workdays) and for how many people, as well as information about who was involved. Each unit selected one person in a leading position in the implementation to complete the logbooks, either as each activity was conducted or retrospectively. Information from the logbooks was used to identify which activities were perceived by personnel units to be related to implementing a more PCC approach and estimations of the time used for this implementation. Only time was reported in the logbooks, no equipment or other expenses were tracked.

In addition, units were asked to provide suggestions (hereafter called unit-specific measures) to evaluate the economic impact of implementing a more PCC approach (Table 1), that leaders in these units viewed as important for understanding the changes in practice induced by PCC. Relevant data on these proposed outcomes were collected from each unit retrospectively, and the units were asked to comment on any potential time trends in the data.

Data were collected for a three-year study period (June 2016-May 2019), although logbook data continued into autumn of 2019 for the psychiatric units and the DD due to previous delays in activities. Activities conducted before the study started (ethics approval in 2017) were filled in retrospectively.

For peer review only

Table 1: Pre-planned unit-specific measures of resource use (Supplementary file S1).

Unit	Planned reporting	
Nephrology /dialysis unit[a]		Distribution between haemodialysis, assisted peritoneal dialysis and unassisted peritoneal dialysis; work hours (adjusted for the number of patients)
Primary care[a]		Number of patients listed; number of visits per patient
Geriatric care and rehabilitation	Unit 1:	Length of stay
	Unit 2:	Work hours[a]; number of patients; work environment follow-up[a]
Psychiatrics[b]	Unit 1:	Length of stay (considering overcrowding)
	Unit 2:	Length of stay and readmissions (considering overcrowding)

[a] Not reported (deviation from planned analyses).

[b] These two units were merged during a large part of the study, and unit-specific measures were thus reported as a combined unit.

Resource use and cost estimation

In analysing implementation programmes, four categories of costs have been suggested: 1) costs for executing implementation strategies, 2) excess costs for service delivery as it changes, 3) opportunity costs to providers and patients, and 4) research/development costs (35). In this study, costs for executing implementation strategies are mainly directed towards centrally organised processes (e.g., seminars). In contrast, the costs for service delivery included activities the units used in operationalising PCC in ordinary practice. This study did not reflect foregone opportunity costs in the care of patients because data collection was not designed for patient-level follow-up. However, it needs to be acknowledged that all resource use in the health system can potentially have an opportunity cost related to an alternative use of existing resources. Activities reported in logbooks were first categorised inductively to identify less aggregated types of resource use and, after that,

For peer review only

deductively according to the above categories. Conversions were made assuming 8-hour workdays and 46 full weeks of work each year.

Costs for used resources were calculated based on the time used for each activity reported in the logbooks. The corresponding costs to the health system in 2019 values were calculated by multiplying the time spent by wage and the related mandatory and negotiated social insurance contribution (37.14% (36)). The mean wage for a nurse employed by a Swedish Region was SEK 34,100 (37), which was used as an approximation for the wage of staff in the health care units as we seldom knew the distribution of staff categories in logbook recordings (i.e., SEK 2,440 per day working, after adjusting for holidays and social insurance contributions, calculated as SEK 34,100*1.3714*12 months divided by 46 weeks working and 5 days per week). The approximate wage for staff employed in the DD was SEK 40-45,000 reported by the development leaders, which in the analysis was approximated to SEK 43,000 (resulting in SEK 3,077 per day working). Costs for the DD were also calculated using information from the regional budget documentation for this function, including a set budget for each year during the study. The set budget, for instance, was to cover 80% of full-time employment. The budget was further contextualised using the total population of Region Dalarna (approximately 281,000 inhabitants) and the total health care spending during the study period. Year-end reports from the region reported costs of SEK 8,985-11,106 million (38) during the years 2016-2018. Costs were analysed from the health systems perspective, including all costs to the care organization, and expressed in 2019 value. No discounting was deemed necessary because costs were only reported descriptively.

Statistical analyses

Time reported in the logbooks was reported descriptively by types of activity associated with implementing a more PCC approach (Supplementary file S2). The unit-specific outcomes were reported separately for each unit, using graphs to illustrate trends over time. The linear equation was used to indicate trends in the length of stay while survival analysis was used to examine time to

For peer review only - http://bmjopen.bmj.com/site/about/guidelines.xhtml

readmission. Where applicable, analyses were adjusted for overcrowding during the initial hospitalization. All analyses were conducted using Stata Statistical Software: Release 17.0. College Station, TX: StataCorp LLC.

Patient and Public Involvement

This project did not include patient or public involvement in developing the research questions, design, conduct, choice of outcome measures, or recruitment.

Results

The time reported in logbooks was between 3 and 13 working days per staff member in the participating units (Table 2), although the time spent was not equally distributed among the teams in each unit. In total, time reported in logbooks from the DD corresponded to 267 full days of work (account for work done by the development unit overall, including the time used by two assigned development leaders) and 77-281 full days of work per unit for the health care units. These figures corresponded to approximately 3-13 working days per staff member in each health care unit over the three-year study period (mean 5.5 working days; median 4 working days). The cumulative distribution of time reported in logbooks indicate that some units reported an equal workload associated with the implementation between years while others reported a more varied pattern of workload (Fig 1). Moreover, activities interpreted as associated with the implementation strategies were in most units corresponding to the majority of time reported throughout the study period.

For peer review only

Table 2: Resource use reported in logbooks by categories of costs and organisational units.

Organisational unit (approx. employees)	Total	Cost categories[a]		
		Implementation strategies	Service delivery	Research/development
	working days[b] (per employee)	working days[b] (per cent)	working days[b] (per cent)	working days[b] (per cent)
Geriatric unit 1 (50)	185 (4)	19 (10)	161 (87)	5 (3)
Geriatric unit 2 (50)	167 (3)	95 (57)	67 (40)	5 (3)
Nephrology unit (40)	162 (4)	68 (42)	74 (46)	20 (12)
Primary care unit (70)	275 (4)	44 (16)	212 (77)	19 (7)
Psychiatric unit 1 (20)	263 (13)	15 (6)	236 (90)	12 (5)

For peer review only - http://bmjopen.bmj.com/site/about/guidelines.xhtml

Psychiatric unit 2 (20)	95 (5)	19 (20)	74 (78)	2 (2)
DD (18)	267 (-)[c]	234 (88)	5 (2)	28 (10)

Numbers are rounded.

[a] Developed from previously reported cost categories in implementation programmes: 1) costs for executing implementation strategies, 2) excess costs for service delivery as implementation/service changes, 3) opportunity costs to providers and patients, and 4) research/development costs (35). No account was taken for changes in costs for direct patient care (category 3).

[b] Assuming 8-hour working days.

[c] Time per employee was not calculated for the Department of Development as the time used was mainly spent by the two persons engaged in providing operational support.

Abbreviations: DD = Department for development.

Fig 1: Accumulation of working days for the Department for Development and the care units by categories of costs in implementation programmes.

Figure legend: * Resource use analysed by year (other graphs are based on actual dates).

Activities categorised as being related to the implementation strategies included planning and preparatory work for the learning seminars, as well as conducting/participating in those seminars. For the DD, service delivery was interpreted to include participation in regional decision making, administrative work, reporting to the region and collaboration with other organisations (e.g., unions). For the units, it included IT solutions, reporting, educational activities, development of teams, and care development. Research and development costs comprised interactions with the research team and external collaboration with other regions. A more detailed list of the activities reported by the units and the distribution of reported time between activities are reported in STable 1 (Supplementary file S3). Training of staff was a major part of this implementation programme. For some employees, the training included up to three seminars (7-8 hours/seminar, difference based on transportation). In most units additional training sessions were added (for a selected group or all employees) to facilitate the implementation of a more PCC approach, such as training in communication techniques.

For peer review only

Estimated salary costs are listed in Table 3. The DD included both personnel (corresponding to 80% full-time over the whole period, corresponding to 552 working days[1]) and resources for training (seminars and workshops), development work and IT resources, as well as internal and external communication. For 2016, 2017, 2018, and 2019, the annual budget was SEK 500,000, SEK 600,000, SEK 600,000, and SEK 880,000, respectively, for the work organised by the DD during the period 2016-2019 (i.e., total budget: SEK 2,580,000, of which approximately SEK 1,698,342 in salary costs for the 80% employment to support the units, as detailed in the action plan). Based on the target population of Region Dalarna, the total cost of the DD (salaries and other expenses) corresponds to SEK 2.30 per citizen per year. Thus, the DD budget corresponded to 0.009 % of the total health care budget over the studied period (June 2016-May 2019). The approximate exchange rate is SEK 10 ≈ EUR 1 (exchange rate in 2019 was mean SEK 10.5892 [range SEK 10.1874-10.9056] per EUR 1 (39)), which means the total budget for the DD was approximately EUR 258,000 during 2016-2019.

Table 3: Costs for time reported in logbooks by categories of costs and organisational units. Costs include wages and social insurance contributions but excludes overhead costs (e.g., facilities).

Organisational unit (approx. employees)	Total	Cost categories[a]		
		Implementation strategies	Service delivery	Research/development
	SEK (percent of total cost)	SEK	SEK	SEK
Geriatric unit 1 (50)	451,673 (12)	46,358	393,535	11,779
Geriatric unit 2 (50)	406,930 (11)	231,638	163,778	11,514
Nephrology unit (40)	394,811 (11)	165,761	179,638	49,413
Primary care unit (70)	669,922 (19)	107,661	516,039	46,223
Psychiatric unit 1 (20)	642,001 (18)	36,598	575,740	29,662
Psychiatric unit 2 (20)	231,582 (6)	46,358	181,493	3,731
DD (18)	822,633 (23)	720,334	15,384	86,916
Total cost	3,619,552 (100)	1,354,708	2,025,607	239,238

Numbers are rounded.
[a] Developed from previously reported cost categories in implementation programmes: 1) costs for executing implementation strategies, 2) excess costs for service delivery as implementation/service changes, 3) opportunity costs to providers and patients, and 4) research/development costs (35). No account was taken for changes in costs for direct patient care (category 3).
Abbreviations: DD = Department for development.

[1] Calculated as 80% of full-time over 3 years (with full time being 46 weeks per year).

For peer review only - http://bmjopen.bmj.com/site/about/guidelines.xhtml
For peer review only

Salary costs associated with time reported in logbooks indicated 23% of costs for this implementation occurred in the DD. Including the total budgeted funding for the DD (SEK 2,580,000), their proportion increased to 48% of the total cost.

Unit specific measures

The first geriatric unit (SFigure 1) and the psychiatric units (combined in SFigure 2) reported a decrease in the average length of stay among their patient populations during the study period. Further examination of data from the psychiatric units showed that this trend was not explained by overcrowding (results not shown). Time to readmission within the first 10 months after discharge was similar between years in psychiatric units (combined in SFigure 3), although last year data were affected by data availability.

The second geriatric unit reported a similar number of discharges across years (range 675-710) but refrained from providing further unit-specific data due to significant changes in the organisation (textbox 1). The nephrology and primary care units also refrained from delivering the planned outcome data due to other major changes in their work processes conducted during the same period as the implementation of a more person-centred care approach. Consequently, these data are only slightly related to the change under study. The primary care unit had approximately 14,000 listed patients throughout the study period.

For peer review only

Textbox 1: Staff turnover and changing methods for prioritising patients: an example from Geriatric care and rehabilitation (Geriatric unit 2).

Initial contacts to discuss the evaluation of resource use were able to identify several aspects that could be relevant to follow during the development towards a more PCC approach, including work hours, number of patients, work environment follow-up, and information that should be accessible

For peer review only - http://bmjopen.bmj.com/site/about/guidelines.xhtml

through the administrative registers. These aspects were considered especially important due to the shortage of registered nurses.

When the project was nearing its end, new contacts were made. None (or very few) of the people involved in the project's launch remained in the organisation in 2019 due to changing roles or retirement. Concerns were expressed that they had not had time to actively work on the person-centeredness due to staff shortages and related downsizing of patient beds. The reduction in patient beds was described as having a budget for 18 patients but only staff enough to admit 10. They had handled the lack of nurses by changing from a registered nurse and an assistant nurse working in pairs to each nurse working with two assistants and transferring tasks to the physicians. Daily discussions were held to ensure that those in most need of the services provided by the geriatrics unit were cared for at the unit, and not moved to other sections of the hospital due to overcrowding.

Thus, it was concluded that it would not be relevant to evaluate any of the initial planned unit-specific outcomes given that the implementation process of a more PCC approach had been given a secondary role compared to other changes in the unit. In table 2 it can be seen that this unit used among the lowest number of working days per person of all included units and had the highest percentage (57%) of that work distributed to the initial planning and participation in learning seminars (cost category implementation strategies). Combined, the perceived secondary role of the implementation process and the discrepant time distribution compared to other included units can indicate the implementation process was not complete.

Discussion

The health care units logged on average 5.5 working days per staff member for implementing a more PCC approach, but the number of days varied largely between units (range 3-13 working days). In the healthcare units, 5-57% of the time reported in logbooks was assessed as being used for implementation strategies, 40-90% for service delivery, and 3-12% for research/development. As

For peer review only - http://bmjopen.bmj.com/site/about/guidelines.xhtml

expected, the distribution of time used by the DD staff differed considerably from that of the other participating units, with most of the logged time (88%) assigned to implementation strategies. While the time spent and salary costs associated with the implementation process were considerable, usually corresponding to at least 0.5-1 year of full-time employment per unit, the total cost was small compared to the entire health care budget. Although budgeting for this implementation was only available for the DD, at least half of the costs occurred in the other healthcare units. Unit-specific outcomes from three of the units showed no clear effect of the implementation, and in general, the healthcare units reported that other factors had affected their throughput more during this study period than the implementation of a more PCC.

To our knowledge, this is the first study investigating the different components contributing to the time and costs spent on implementing a more PCC approach. Several studies have showcased how implementation costs should be measured (35,40–42), but there is a shortage of studies measuring the costs from an implementation standpoint (43,44). The strength of the study is that it was conducted by an independent research body that included researchers from different disciplines relevant to the interpretation of the results. Another strength was that the included units were given multiple opportunities to clarify and correct any oversights in their records (such as not writing the number of participants during a specific action) and potential misunderstandings regarding interpretation. Although the independence of the researchers was a strength, it also contributed to the main limitation of the study, the data collection process. The staff had no time set off for the logbooks and were not provided with extra time or staff to conduct the implementation process. Some reporters filled in large parts of the logbooks retroactively (not only 2016 data), potentially resulting in recall bias, and in some cases, one of our research group members (MT or HF) took part in this work to record previous activities. It should be noted that for one of the units with the highest working day estimate in relation to their staff, one of the researchers supported the staff member in filling out the logbooks which could indicate the staff member was reminded of more tasks having been conducted than would otherwise have been the case. However, the higher estimate could also

For peer review only

For peer review only - http://bmjopen.bmj.com/site/about/guidelines.xhtml

be the result of this being one of two units that were merged during part of the study, thus making the division of time spent for each of these units difficult to distinguish.

While all unit-specific outcomes had been identified by each healthcare unit as important aspects to follow during this change process, most units chose in the end to not providing the data due to being more affected by other changes in the workplaces. For the units providing these data there is still an assumption that most of the observed changes were due to other factors than the implementation of a more PCC. The reason for initiating the collection of unit-specific outcomes was to make the evaluation more relevant to the participating healthcare units and similar units elsewhere, and to ensure that the implementation of a more PCC approach was at least not associated with any large negative impact on patient throughput. However, due to other parallel changes in these units it did not provide any conclusive results. The commitment shown by staff in the participating units is exemplified by them participating in the change process and providing materials and responses to questions during analysis, regardless of the lack of time provided for their participation. This commitment can also be discussed regarding the planned observational approach of the study. Here, it can be argued that by engaging in research and data collection, the staff involved in the implementation may have been affected (i.e., through social desirability bias or being reminded of the implementation process by the researchers) and thus to a larger extent interacting with it. While initially intended, we did not have logbooks from leaders (Chief executives) of this implementation process at the main organisational level in the region, but that should have implied a small cost compared to that of changing care practice. It should also be acknowledged that the division of costs by categories is not self-evident. Costs for changes in service delivery could also to some extent be seen as costs for implementation strategies. For example, the initial learning seminars were assumed to be part of the implementation strategies. However, if the health care unit later decided that in their work to change practice, the staff needed special training (e.g., in interprofessional rounds or motivational interviewing), we interpreted this as part of changing how service was delivered. An alternative interpretation would have been to see this training as part of an iterative process of

For peer review only - http://bmjopen.bmj.com/site/about/guidelines.xhtml

implementation strategies conducted at different levels of the organisation, i.e., the study distinguishes between centrally planned implementation strategies and strategies conducted by the health care delivery organisation (45). When considering the limitations of the study, our estimated working days and costs should be interpreted with caution. These findings are only part of a picture that needs to be further developed in future studies and frameworks to assess the economic impact of implementation.

Using the cost components suggested by Wagner et al. (40) as a basis, additional costs would include overhead costs (e.g., facilities). Moreover, the inclusion of research and development costs should be broken down into what would be sunk costs (i.e., one-time investments) versus costs for development and scaling up (such as communications within the region to support others) versus costs that are solely for research purposes (i.e., the research interviews). However, it has been argued that the costs of research should be reported (46), not to inform clinical practice but to assess the costs associated with evaluation. Here, we are only reporting on the costs for the region to participate in the research study. We hope our approach and findings might help others design similar studies to follow implementation processes more in depth.

Changes in health care can aim to either improve effectiveness, i.e., save money while producing at least as much health as before, or increase care quality, or a combination of the two (47). Today, there is a growing body of evidence of improved patient outcomes and possibilities for cost savings by shifting to PCC from randomised controlled trials and quasi-experimental studies in Sweden and internationally (10,28,29,48,49). In comparison, little is known about the “hidden” costs of preparatory work, training, and monitoring outcomes during implementation (44), costs that are investigated in this study. In a recent systematic review of the literature, only six studies were identified that specified such costs. It has been argued that any implementation effort should be preceded by ex-ante modelling to compare the expected returns of implementing the intended

For peer review only - http://bmjopen.bmj.com/site/about/guidelines.xhtml

change to the predicted costs of implementing the change (35,43,50). However, this is not always the case, or the results are at least not available to the research community.

One crucial aspect of the studied change process was that it was made clear that health care units would not be provided with extra time or other resources for the implementation. This was reported by both the central regional organisation and the management in each health care unit, with staff shortage being the main reason. However, we fund that units implementing more PCC used a considerable amount of time for this implementation process. Thus, the time reported in logbooks could be interpreted to refer to the time that otherwise would have been used for other work in the health care units. Had the implementation of more PCC not been made, the reported time would still have been used in the units. A hypothetical comparator, an alternative intervention, could have used the same amount of time to implement some other change in practice, such as developing and implementing clinical guidelines or care paths (which thus would suggest the opportunity cost of this implementation process). Because several units had assigned quality developers or specialist nurse students with tasks associated with the implementation process, which equalled several working days spent per staff member, the used time likely offset other tasks otherwise conducted by the staff. In addition, staff in some settings expressed that it was time-consuming to provide PCC in the immediate time frame but it could potentially be beneficial later in the care process (20). Together with recent reporting that increased person-centredness was associated with higher job strain (18), it is likely that additional resources during the initial period had resulted in improved uptake.

It should also be noted that the possibility to influence resource use is probably influenced by the patient groups in each health care unit and to what extent work with these patients is already streamlined. Considering a patient group for which clinical guidelines determines the frequency of follow-up, there is less opportunity to change the number of visits and thus costs will be similar even if the care changes. If there is instead a patient group experiencing unmet needs and much acute

For peer review only - http://bmjopen.bmj.com/site/about/guidelines.xhtml

unplanned care, it can be assumed that changing how patients experience their health care can change how many visits are needed.

While several of the health care units expressed that they had not completed the implementation within the study period, assessing its success need to be based on still ongoing studies of patients' experiences. However, the findings clearly demonstrate that there is a non-zero cost of implementing a more PCC approach; costs that should be acknowledged in future research and implementation processes. The study also points towards potential improvements in how to study implementation costs, through e.g., recurrent questionnaires instead of logbooks that are collected at the end of the study period. Furthermore, due to the reported high staff turnover, the costs for changes in service delivery may to some extent continue in training of new staff. Considering our findings in light of recent updates on the use of economic evaluation in implementation science to guide decision-makers (51,52), future studies should thus distinguish all costs associated with implementation science, including implementation costs, intervention costs, and downstream costs (17) of a more PCC approach, as well as for other healthcare programmes.

Conclusions

The study found that a large part of resources used for this implementation of more PCC occurred in the DD, although at least half of the costs occurred in the healthcare units. Our findings suggest that the main costs associated with implementing a more PCC approach in ordinary practice resulted from implementing strategies and service delivery. In contrast, research and development costs were small by comparison. Moreover, the cost of providing a central support function corresponds to a tiny proportion of the total health budget. While there are limitations in how the study was conducted, it clearly demonstrates a non-zero cost of implementing a more PCC approach, thus implicating that future research should capture costs. Not accounting for the added strain on healthcare units can result in delay or inability to implement the new care model.

For peer review only - http://bmjopen.bmj.com/site/about/guidelines.xhtml

Supporting information captions

Supplementary file S1: Study protocol

Supplementary file S2: Analysis dataset

Supplementary file S3: Supplementary tables and figures

Funding statement

The study was financed by the University of Gothenburg Centre for Person-Centred Care (GPCC) and Dalarna University. The funding body had no role in the design of the study, in collection, analysis, and interpretation of data, or in writing the manuscript.

Competing interest’s statement

The authors declare that they have no competing interests.

Author's contribution

HG, MT, HF, and LW contributed to the design of the work; HG, MT and HF contributed to the acquisition, analysis, and interpretation of the data; HG drafted the manuscript while MT, HF, and LW substantively contributed to the final manuscript; HG, MT, HF, and LW all have approved the submitted version and agree both to be personally accountable for the author's contributions and to ensure that questions related to the accuracy or integrity of any part of the work, even ones in which the author was not personally involved, are appropriately investigated, resolved, and the resolution documented in the literature.

Acknowledgments

We thank all contributing health care units. Thanks also to Dr Leslie Shaps for language editing.

For peer review only

For peer review only - http://bmjopen.bmj.com/site/about/guidelines.xhtml

Data sharing statement

All relevant data are within the manuscript and its Supporting Information files (Supplementary file S2).

Ethics approval statement

The study was approved by the regional ethics committee in Uppsala (approval number: 2017/195). Logbooks were filled in by managers/project leaders in each health care unit who had consented to this registration (verbally consented at the start of the study, and later through providing the information for analysis). Unit specific data were obtained from administrative systems without information on individual patients or staff, by the same consenting managers/project leaders. There was thus no data collection (nor informed consent) from patients or individual staff. Results for each unit were communicated to provide an opportunity for the units to question and correct misunderstandings. All methods were performed in accordance with relevant guidelines and regulations, and in accordance with the ethical approval for this study.

References

1. Nolte E, Merkur S, Anell A, European Observatory on Health Systems and Policies, editors. Achieving person-centred health systems: evidence, strategies and challenges. Cambridge; New York, NY: Cambridge University Press; 2020. (European Observatory on Health Systems and Policies).

2. Rosengren K, Brannefors P, Carlstrom E. Adoption of the concept of person-centred care into discourse in Europe: a systematic literature review. J Health Organ Manag. 2021 Sep 13;35(9):265–80.

3. Sverige Utredningen Samordnad utveckling för god och nära vård. God och nära vård: en primärvårdsreform [Good quality, local health care – A primary care reform] [Internet]. Stockholm; Stockholm: Norstedts Juridik; Elanders Sverige AB; 2018. (Statens offentliga utredningar : SOU). Report No.: 9789138248065. Available from: https://www.regeringen.se/rattsliga-dokument/statens-offentliga-utredningar/2018/06/sou-201839/

4. Mead N, Bower P. Patient-centredness: a conceptual framework and review of the empirical literature. SocSciMed. 2000;51(7):1087–110.

5. Ekman I, Swedberg K, Taft C, Lindseth A, Norberg A, Brink E, et al. Person-centered care--ready for prime time. EurJCardiovascNurs. 2011;10(4):248–51.

For peer review only

6. Harding E, Wait S, Scrutton J. The state of play in person-centred care: A pragmatic review of how person-centred care is defined, applied and measured [Internet]. The Health Policy Partnership Ltd; 2015 p. 1–140. Available from: http://www.healthpolicypartnership.com/person-centred-care/

7. Håkansson Eklund J, Holmström IK, Kumlin T, Kaminsky E, Skoglund K, Höglander J, et al. “Same same or different?” A review of reviews of person-centered and patient-centered care. Patient Educ Couns. 2019;102(1):3–11.

8. World Health Organization. Framework on integrated people-centred health services. WHO; 2016 p. 1–12.

9. Sharma T, Bamford M, Dodman D. Person-centred care: an overview of reviews. ContempNurse. 2015;51(2–3):107–20.

10. Hansson E, Ekman I, Swedberg K, Wolf A, Dudas K, Ehlers L, et al. Person-centred care for patients with chronic heart failure - a cost-utility analysis. EurJCardiovascNurs. 2015;15(4):276–84.

11. Sahlen KG, Boman K, Brännstrom M. A cost-effectiveness study of person-centered integrated heart failure and palliative home care: Based on a randomized controlled trial. PalliatMed. 2016;30(3):296–302.

12. Swedberg K, Cawley D, Ekman I, Rogers HL, Antonic D, Behmane D, et al. Testing cost containment of future healthcare with maintained or improved quality-The COSTCARES project. Health Sci Rep. 2021 Jun;4(2):e309.

13. Nkhoma KB, Cook A, Giusti A, Farrant L, Petrus R, Petersen I, et al. A systematic review of impact of person-centred interventions for serious physical illness in terms of outcomes and costs. BMJ Open. 2022 Jul 13;12(7):e054386.

14. Chenoweth L, King MT, Jeon YH, Brodaty H, Stein-Parbury J, Norman R, et al. Caring for Aged Dementia Care Resident Study (CADRES) of person-centred care, dementia-care mapping, and usual care in dementia: a cluster-randomised trial. Lancet Neurol. 2009 Apr;8(4):317–25.

15. Chenoweth L, Forbes I, Fleming R, King MT, Stein-Parbury J, Luscombe G, et al. PerCEN: a cluster randomized controlled trial of person-centered residential care and environment for people with dementia. Int Psychogeriatr. 2014 Jul;26(7):1147–60.

16. Jones RA, Dougherty M, Martin S. Program evaluation of a unit reengineered for patient-focused care. Holist Nurs Pract. 1997 Apr;11(3):31–46.

17. Gold HT, McDermott C, Hoomans T, Wagner TH. Cost data in implementation science: categories and approaches to costing. Implement Sci. 2022 Jan 28;17(1):11.

18. Van Diepen C, Fors A, Ekman I, Bertilsson M, Hensing G. Associations between person-centred care and job strain, stress of conscience, and intent to leave among hospital personnel. J Clin Nurs. 2022 Mar;31(5–6):612–22.

19. Fridberg H, Wallin L, Wallengren C, Kottorp A, Forsman H, Tistad M. Development and evaluation of the measurement properties of a generic questionnaire measuring patient perceptions of person-centred care. BMC Health Serv Res. 2020 Oct 20;20(1):960.

For peer review only

For peer review only - http://bmjopen.bmj.com/site/about/guidelines.xhtml

20. Fridberg H, Wallin L, Tistad M. [The innovation characteristics of person-centred care as perceived by healthcare professionals: an interview study employing a deductive-inductive content analysis guided by the consolidated framework for implementation research. BMC Health Services Research. 2021 Sep 3;21(1):904.

21. Fridberg H, Wallin L, Tistad M. Operationalisation of person-centred care in a real-world setting: a case study with six embedded units. BMC Health Serv Res. 2022 Sep 14;22(1):1160.

22. Fridberg H, Wallin L, Tistad M. Tracking, naming, specifying, and comparing implementation strategies for person-centred care in a real-world setting: a case study with seven embedded units. BMC Health Serv Res. 2022 Nov 24;22(1):1409.

23. Tistad M, Wallin L, Carlström E. A comparison of three organisational levels in one health care region in Sweden implementing person-centred care: Coupled, decoupled or recoupled in a complex organization. BMC Health Serv Res. 2022 Feb 14;22(1):196.

24. Other languages - Region Dalarna [Internet]. Region Dalarna. [cited 2022 Jun 29]. Available from: https://www.regiondalarna.se/ovrigt/om-webbplatsen/other-languages/

25. Glenngård AH. The Swedish Health Care System. In: International Profiles of Health Care Systems 2020 [Internet]. The Commonwealth Fund; 2020. p. 1–228. Available from: https://www.commonwealthfund.org/international-health-policy-center/countries

26. Britten N, Ekman I, Naldemirci Ö, Javinger M, Hedman H, Wolf A. Learning from Gothenburg model of person centred healthcare. BMJ. 2020 01;370:m2738.

27. Olsson LE, Karlsson J, Ekman I. The integrated care pathway reduced the number of hospital days by half: a prospective comparative study of patients with acute hip fracture. JOrthopSurgRes. 2006;1(Journal Article):3-799X-1–3.

28. Gyllensten H, Koinberg I, Carlstrom E, Olsson LE, Hansson Olofsson E. Economic evaluation of a person-centred care intervention in head and neck oncology: results from a randomized controlled trial. SupportCare Cancer. 2019;27(5):1825–34.

29. Pirhonen L, Bolin K, Olofsson EH, Fors A, Ekman I, Swedberg K, et al. Person-Centred Care in Patients with Acute Coronary Syndrome: Cost-Effectiveness Analysis Alongside a Randomised Controlled Trial. Pharmacoecon Open. 2019;3(4):495–504.

30. Skivington K, Matthews L, Simpson SA, Craig P, Baird J, Blazeby JM, et al. A new framework for developing and evaluating complex interventions: update of Medical Research Council guidance. BMJ. 2021 Sep 30;374:n2061.

31. Craig P, Dieppe P, Macintyre M, Michie S, Nazareth I, Petticrew M. Developing and evaluating complex interventions: new guidance [Internet]. Medical Research Council; 2006 p. 1–39. Available from: https://www.mrc.ac.uk/documents/pdf/complex-interventions-guidance/

32. Moore GF, Audrey S, Barker M, Bond L, Bonell C, Hardeman W, et al. Process evaluation of complex interventions: Medical Research Council guidance. BMJ. 2015;350:h1258.

33. Proctor EK, Landsverk J, Aarons G, Chambers D, Glisson C, Mittman B. Implementation research in mental health services: an emerging science with conceptual, methodological, and training challenges. AdmPolicy MentHealth. 2009;36(1):24–34.

For peer review only - http://bmjopen.bmj.com/site/about/guidelines.xhtml

34. Dawson A, Sim J. The nature and ethics of natural experiments. J Med Ethics. 2015 Oct;41(10):848–53.

35. Hoomans T, Severens JL. Economic evaluation of implementation strategies in health care. Implement Sci. 2014 Dec 18;9:168.

36. Arbetsgivaravgifter [General payroll tax] [Internet]. Sveriges Kommuner och Regioner [Swedish Association of Local Authorities and Regions]. [cited 2020 Apr 29]. Available from: https://skr.se/ekonomijuridikstatistik/ekonomi/budgetochplanering/arbetsgivaravgifter.1290.html

37. Lönesök – Hur mycket tjänar...? [Wage search - How much do ... earn?] [Internet]. Sverige i siffror. [cited 2020 Apr 29]. Available from: http://www.scb.se/hitta-statistik/sverige-i-siffror/lonesok/

38. Årsredovisning - Region Dalarna [Year-end reports - Region Dalarna] [Internet]. [cited 2021 Sep 2]. Available from: https://www.regiondalarna.se/om-regionen/arsredovisning/

39. Årsgenomsnitt valutakurser (ackumulerat) 2019 [Annual average exchange rates (accumulated) 2019] [Internet]. [cited 2022 Jun 30]. Available from: https://www.riksbank.se/sv/statistik/sok-rantor--valutakurser/arsgenomsnitt-valutakurser/

40. Wagner TH, Yoon J, Jacobs JC, So A, Kilbourne AM, Yu W, et al. Estimating Costs of an Implementation Intervention. Med Decis Making. 2020 Nov;40(8):959–67.

41. Krebs E, Nosyk B. Cost-Effectiveness Analysis in Implementation Science: a Research Agenda and Call for Wider Application. Curr HIV/AIDS Rep. 2021 Jun;18(3):176–85.

42. Powell BJ, Fernandez ME, Williams NJ, Aarons GA, Beidas RS, Lewis CC, et al. Enhancing the Impact of Implementation Strategies in Healthcare: A Research Agenda. Front Public Health. 2019;7:3.

43. Reeves P, Edmunds K, Searles A, Wiggers J. Economic evaluations of public health implementation-interventions: a systematic review and guideline for practice. Public Health. 2019 Apr;169:101–13.

44. Roberts SLE, Healey A, Sevdalis N. Use of health economic evaluation in the implementation and improvement science fields—a systematic literature review. Implementation Science. 2019 Jul 15;14(1):72.

45. Leeman J, Birken SA, Powell BJ, Rohweder C, Shea CM. Beyond “implementation strategies”: classifying the full range of strategies used in implementation science and practice. Implement Sci. 2017 Nov 3;12(1):125.

46. Visser BJ, Buijink AWG, Grobusch MP. Reporting of medical research costs. Improving transparency and reproducibility of medical research. Methods Inf Med. 2014;53(4):329–31.

47. Chisholm D, Evans DB. Economic evaluation in health: saving money or improving care? Journal of Medical Economics. 2007 Jan 1;10(3):325–37.

48. Pirhonen L, Gyllensten H, Fors A, Bolin K. Modelling the cost-effectiveness of person-centred care for patients with acute coronary syndrome. Eur J Health Econ. 2020 Sep 7;21(9):1317–27.

For peer review only

For peer review only - http://bmjopen.bmj.com/site/about/guidelines.xhtml

49. Pirhonen L, Gyllensten H, Olofsson EH, Fors A, Ali L, Ekman I, et al. The cost-effectiveness of person-centred care provided to patients with chronic heart failure and/or chronic obstructive pulmonary disease. Health Policy OPEN. 2020 Dec 1;1:100005.

50. Sutton M, Garfield-Birkbeck S, Martin G, Meacock R, Morris S, Sculpher M, et al. Economic analysis of service and delivery interventions in health care [Internet]. Southampton (UK): NIHR Journals Library; 2018 [cited 2020 Mar 20]. (Health Services and Delivery Research). Available from: http://www.ncbi.nlm.nih.gov/books/NBK481909/

51. Saldana L, Ritzwoller DP, Campbell M, Block EP. Using economic evaluations in implementation science to increase transparency in costs and outcomes for organizational decision-makers. Implement Sci Commun. 2022 Apr 11;3(1):40.

52. O'Leary MC, Hassmiller Lich K, Frerichs L, Leeman J, Reuland DS, Wheeler SB. Extending analytic methods for economic evaluation in implementation science. Implement Sci. 2022 Apr 15;17(1):27.

For peer review only

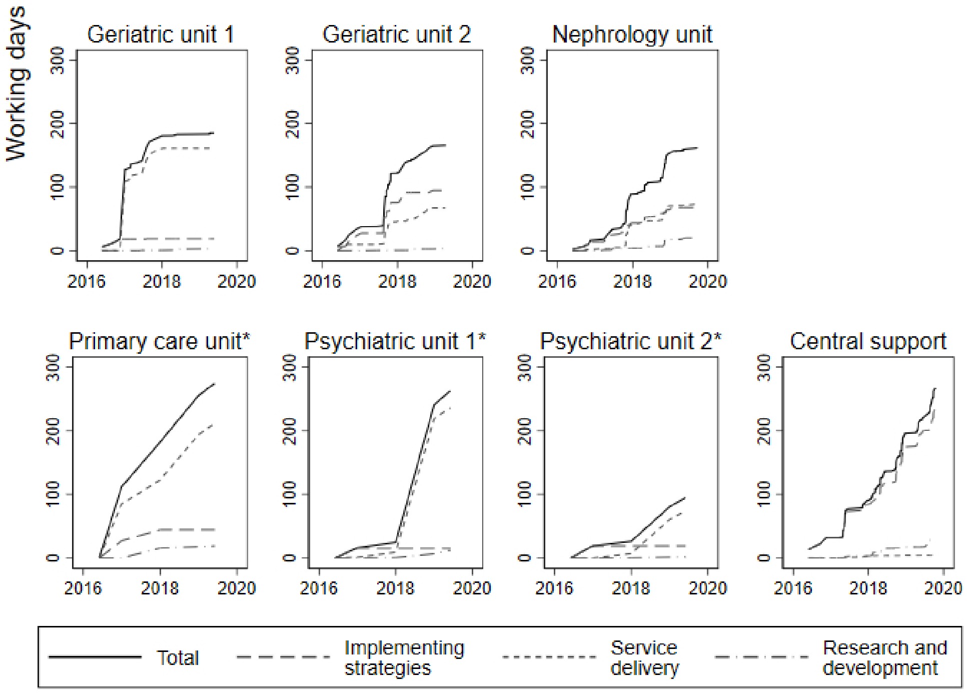

Accumulation of working days for the Department for Development and the care units by categories of costs in implementation programmes.
Figure legend: * Resource use analysed by year (other graphs are based on actual dates).

246x178mm (72 x 72 DPI)

Health economics of implementing person-centred care: the IMPROVE project

Suggested authors (order to be decided)

Hanna Gyllensten, Malin Tistad, Helena Fridberg, Eric Carlström, Lars Wallin

Background

Healthcare systems in many countries experiences increasing economic demands, both through the development of new technologies and treatments, and through a changing age distribution in the population resulting in more people with multiple chronic conditions.

Person-centred care (PCC) has been promoted both as a means to handle dissatisfaction with healthcare access and delivery,(1) and as potentially cost-saving or cost containing through more effective use of resources.(2) PCC acknowledges and endorses every person´s resources, interests, and needs, comprising shared responsibility and power, as well as a coordinated care and treatment.(3–5) It is related to the so called integrated people-centred health services promoted by the World Health Organization(6) albeit without the community perspective included in that framework.

However, PCC it is often described as a complex intervention; an intervention containing several interacting components,(7,8) through including elements tailored to each participant, activities achieved in different ways, and with multiple and sometimes varying outcomes and goals for different participants. Thus, research into its use and effects needs to handle the complexity involved,(7) including a thorough understanding of how the intervention is produced through e.g., process evaluation of the intervention created.(9) In a conceptual model for such implementation research, Procter and colleagues elaborated on the outcomes evaluation, including intervention outcomes, service outcomes and client outcomes.(10)

This study is, as far as we know one of few measuring the cost of implementing person-centred care. Several studies suggests cost-saving as one reason to implement person-centred care,(2,11) but the cost to implement the method is seldom mentioned. Even though, person-centred care has an extensive impact on the heath-care industry. In Sweden legislators and health care providers has introduced person-centred care on the agenda of development and management-control efforts is more and more about implementing person-centred care. In this study we measure the cost of such an implementation.

Aims

The aims of this project was to map the resources used in implementing PCC, in the healthcare unit as well as on a regional level. A secondary aim was to explore how this implementation affected the budget, from the healthcare provider's perspective.

Ursprunglig formulering:

• Att kartlägga resursförbrukning för att införa PCV på lokal nivå respektive på regional/landstingsnivå.

• Att undersöka hur landstingets budget påverkas av att införa PCV.

For peer review only - http://bmjopen.bmj.com/site/about/guidelines.xhtml

Methods

Contextual factors

Region Dalarna decided in 2016 to promote a more person-centred care approach throughout the public healthcare system, as part of their work towards efficient healthcare practices (in parallel with other ongoing projects within the so called "Struktur- och förändringsarbetet", including projects related to priority setting and resource allocation in local health systems). As part of this implementation process, changes were made on micro, meso, and macro level in the region. The *Implementing person centred care: Process evaluation of strategies, leadership and health economy* (IMPROVE) project was established to follow this implementation process. In the project, six healthcare units were followed to explore the strategies used to implement a more person-centred care and the associated outcomes, as well as the policies and leadership related aspects of this process. Data was collected through logbooks, questionnaires, interviews and review of medical records, to give a comprehensive overview of how the process evolved over time.

The implementation process studied in the IMPROVE project was introduced and implemented by Region Dalarna. The process evaluation was conducted in parallel by researchers from collaborating universities partnering with staff in the healthcare units.

Data collection

Six healthcare units were included in the process evaluation, selected based on being early adapters to the implementation of PCC. Included healthcare units were followed from six months before to 18 months after the introduction of PCC. The units included were specialized in nephrology, geriatric rehabilitation, psychiatry and primary care, respectively.

Staff involved in introducing PCC were asked to complete logbooks of their activities and hours spent. Information from the logbooks was thus used to identify both which activities were perceived by personnel in different segments of the healthcare organization to be related to implementing PCC, and to estimate the time used for this implementation. Logbooks were also collected from operational support personnel involved in supporting care units during the implementation. Costs for used resources was calculated based on the time used for each type of activity (categorized inductively after data collection) times the average cost per hour for the relevant staff category, retrieved from the healthcare provider.

In addition, included units were asked to provide their own suggestions for evaluating the economic impact of PCC on their practices. Relevant data was collected from each unit retrospectively, and the units were asked to comment on any potential time trends visible in the data.

A third data source was administrative data collected from the region, including economic results, patient quantities, mean length of admission, and hours worked. This information was used to provide a background to the data collected from logbooks and included healthcare units.

Analyses

Descriptive statistics of time used and associated costs, as reported in the logbooks, will be reported by types of activities associated with the implementation of PCC. A bootstrap methodology will be used to calculate 95% confidence intervals, to account for expected skewed distributions of costs.

The unit specific outcomes will be analyzed longitudinally and described separately for each unit. Unit specific measures included:

Nephrology/dialysis unit	Distribution between haemodalysis, assisted peritoneal dialysis and unassisted peritoneal dialysis

For peer review only - http://bmjopen.bmj.com/site/about/guidelines.xhtml

	Work hours (adjusted for number of patients)
Primary care	Number of patients listed<br>Number of visits per patient
Geriatric rehabilitation	Unit 1: Length of stay<br>Unit 2: Work hours, number of patients, work environment follow-up
Psychiatrics	Unit 1: Length of stay (taking overcrowding into consideration)<br>Unit 2: Length of stay and readmissions (taking overcrowding into consideration)<br>These two units were combined during a part of the study, this will also need to be considered.

Regional administrative data will be reported descriptively for each year the study was ongoing.

Suggested tables and figures

Table 1: Overview of respondents to the logbooks, including an overview of the activities reported

Table 2: Hours and associated costs for each type of activity reported in the logbooks

Table 3: Results for the unit specific measures [*may become divided into one per unit or so*]

References

1. Sharma T, Bamford M, Dodman D. Person-centred care: an overview of reviews. ContempNurse. 2015;51(2–3):107–20.

2. Hansson E, Ekman I, Swedberg K, Wolf A, Dudas K, Ehlers L, et al. Person-centred care for patients with chronic heart failure - a cost-utility analysis. EurJCardiovascNurs. 2015;(Journal Article).

3. Mead N, Bower P. Patient-centredness: a conceptual framework and review of the empirical literature. SocSciMed. 2000;51(7):1087–110.

4. Ekman I, Swedberg K, Taft C, Lindseth A, Norberg A, Brink E, et al. Person-centered care--ready for prime time. EurJCardiovascNurs. 2011;10(4):248–51.

5. Harding E, Wait S, Scrutton J. The state of play in person-centred care: A pragmatic review of how person-centred care is defined, applied and measured [Internet]. The Health Policy Partnership Ltd; 2015 p. 1–140. Available from: http://www.healthpolicypartnership.com/person-centred-care/

6. World Health Organization. Framework on integrated people-centred health services. WHO; 2016 p. 1–12.

7. Craig P, Dieppe P, Macintyre M, Michie S, Nazareth I, Petticrew M. Developing and evaluating complex interventions: new guidance [Internet]. Medical Research Council; 2006 p. 1–39. Available from: https://www.mrc.ac.uk/documents/pdf/complex-interventions-guidance/

8. Craig P, Dieppe P, Macintyre S, Michie S, Nazareth I, Petticrew M, et al. Developing and evaluating complex interventions: the new Medical Research Council guidance. BMJ. 2008;337(Journal Article):a1655.

9. Moore GF, Audrey S, Barker M, Bond L, Bonell C, Hardeman W, et al. Process evaluation of complex interventions: Medical Research Council guidance. BMJ. 2015;350(Journal Article):h1258.

For peer review only - http://bmjopen.bmj.com/site/about/guidelines.xhtml

10. Proctor EK, Landsverk J, Aarons G, Chambers D, Glisson C, Mittman B. Implementation research in mental health services: an emerging science with conceptual, methodological, and training challenges. AdmPolicy MentHealth. 2009;36(1):24–34.

11. Sahlen KG, Boman K, Brännstrom M. A cost-effectiveness study of person-centered integrated heart failure and palliative home care: Based on a randomized controlled trial. PalliatMed. 2016;30(3):296–302.

For peer review only

Variable	Value	Label
Row		Row number for sorting
UNIT	1	Geriatric unit 1
	2	Geriatric unit 2
	3	Nephrology unit
	4	Primary care unit
	5	Psychiatric unit 1
	6	Psychiatric unit 2
	7	Central support function
Activity_cat		administration
		care_development
		documentation
		educational_activities
		external_communication
		internal_communication
		it_support
		learning_seminars
		preparatory_work
		reporting
		research
		team_development
		workplace_meetings
COST_CATEGORY	1	costs for executing implementation strategies
	2	excess costs for service delivery as implementation/service changes
	4	research/development costs
Participants		Number of participants
Time_minutes		Total time used by the participants (in minutes)
Staff_cat		Manager
		HCP = health care professionals

Row	UNIT	Activity_cat	COST_CATEGORY	Participants
1	1	learning_seminars	1	7
2	1	learning_seminars	1	7
3	1	learning_seminars	1	7
4	1	care_development	2	1
5	1	educational_activities	2	4
6	1	team_development	2	40
7	1	educational_activities	2	40
8	1	care_development	2	50
9	1	educational_activities	2	5
10	1	team_development	2	20
11	1	educational_activities	2	6
12	1	educational_activities	2	10
13	1	preparatory_work	1	5
14	1	care_development	2	30
15	1	research	4	3
16	1	research	4	4
17	1	research	4	5
18	1	research	4	5
19	1	research	4	3
20	1	research	4	5
21	1	research	4	5
22	1	research	4	1
23	1	research	4	1
24	1	research	4	1
25	1	research	4	1
26	1	research	4	2
27	1	research	4	2
28	1	research	4	2
29	1	research	4	2
30	1	research	4	1
31	1	research	4	1
32	2	learning_seminars	1	8
33	2	care_development	2	1
34	2	learning_seminars	1	9
35	2	learning_seminars	1	9
36	2	preparatory_work	1	10
37	2	care_development	2	1
38	2	preparatory_work	1	25
39	2	care_development	2	10
40	2	care_development	2	1
41	2	preparatory_work	1	12
42	2	preparatory_work	1	7
43	2	preparatory_work	1	1
44	2	learning_seminars	1	2
45	2	educational_activities	2	2
46	2	preparatory_work	1	12
47	2	care_development	2	40
48	2	care_development	2	2
49	2	team_development	2	4

For peer review only

50	2	team_development	2	9
51	2	preparatory_work	1	6
52	2	preparatory_work	1	4
53	2	care_development	2	9
54	2	external_communication	4	2
55	2	team_development	2	5
56	2	team_development	2	10
57	2	care_development	2	5
58	2	preparatory_work	1	25
59	2	research	4	6
60	2	research	4	4
61	2	research	4	4
62	2	research	4	7
63	2	research	4	6
64	2	research	4	4
65	2	research	4	1
66	2	research	4	1
67	2	research	4	1
68	2	research	4	1
69	2	research	4	2
70	2	research	4	2
71	2	research	4	1
72	2	research	4	1
73	3	learning_seminars	1	3
74	3	learning_seminars	1	5
75	3	preparatory_work	1	6
76	3	team_development	2	8
77	3	workplace_meetings	1	25
78	3	team_development	2	4
79	3	learning_seminars	1	7
80	3	team_development	2	7
81	3	research	4	5
82	3	learning_seminars	1	8
83	3	learning_seminars	1	4
84	3	team_development	2	2
85	3	team_development	2	8
86	3	preparatory_work	1	2
87	3	preparatory_work	1	2
88	3	research	4	5
89	3	research	4	5
90	3	preparatory_work	1	1
91	3	research	4	1
92	3	preparatory_work	1	8
93	3	preparatory_work	1	6
94	3	preparatory_work	1	1
95	3	educational_activities	2	2
96	3	learning_seminars	1	4
97	3	preparatory_work	1	5
98	3	preparatory_work	1	3
99	3	preparatory_work	1	3

For peer review only

100	3	preparatory_work	1	3
101	3	educational_activities	2	2
102	3	preparatory_work	1	5
103	3	preparatory_work	1	2
104	3	educational_activities	2	40
105	3	preparatory_work	1	4
106	3	external_communication	4	6
107	3	care_development	2	40
108	3	learning_seminars	1	5
109	3	learning_seminars	1	6
110	3	educational_activities	2	1
111	3	educational_activities	2	1
112	3	documentation	2	1
113	3	care_development	2	12
114	3	preparatory_work	1	5
115	3	preparatory_work	1	2
116	3	educational_activities	2	1
117	3	care_development	2	4
118	3	care_development	2	1
119	3	care_development	2	1
120	3	preparatory_work	1	3
121	3	care_development	2	4
122	3	learning_seminars	1	9
123	3	preparatory_work	1	4
124	3	it_support	2	1
125	3	care_development	2	2
126	3	educational_activities	2	1
127	3	preparatory_work	1	5
128	3	preparatory_work	1	6
129	3	care_development	2	5
130	3	learning_seminars	1	5
131	3	care_development	2	4
132	3	preparatory_work	1	5
133	3	care_development	2	30
134	3	external_communication	4	6
135	3	external_communication	4	6
136	3	preparatory_work	1	6
137	3	educational_activities	2	4
138	3	educational_activities	2	4
139	3	care_development	2	4
140	3	learning_seminars	1	5
141	3	educational_activities	2	4
142	3	learning_seminars	1	4
143	3	care_development	2	4
144	3	care_development	2	5
145	3	educational_activities	2	2
146	3	research	4	3
147	3	research	4	5
148	3	research	4	8
149	3	research	4	5

For peer review only

150	3	research	4	5
151	3	research	4	5
152	3	research	4	4
153	3	research	4	1
154	3	research	4	1
155	3	research	4	1
156	3	research	4	1
157	3	research	4	1
158	3	research	4	1
159	3	research	4	1
160	3	research	4	5
161	3	research	4	5
162	3	research	4	6
163	3	research	4	1
164	3	research	4	1
165	4	learning_seminars	1	9
166	4	learning_seminars	1	9
167	4	preparatory_work	1	1
168	4	preparatory_work	1	1
169	4	external_communication	1	2
170	4	preparatory_work	1	2
171	4	preparatory_work	1	1
172	4	educational_activities	2	35
173	4	educational_activities	2	35
174	4	learning_seminars	1	7
175	4	documentation	1	1
176	4	care_development	2	7
177	4	workplace_meetings	2	70
178	4	external_communication	4	1
179	4	documentation	1	1
180	4	care_development	2	1
181	4	care_development	2	11
182	4	care_development	2	6
183	4	care_development	2	11
184	4	workplace_meetings	2	60
185	4	care_development	2	11
186	4	preparatory_work	4	1
187	4	preparatory_work	4	2
188	4	research	4	6
189	4	external_communication	4	11
190	4	research	4	6
191	4	external_communication	4	6
192	4	learning_seminars	1	9
193	4	learning_seminars	1	8
194	4	educational_activities	2	8
195	4	educational_activities	2	9
196	4	educational_activities	2	9
197	4	educational_activities	2	9
198	4	workplace_meetings	2	70
199	4	documentation	2	1

For peer review only

200	4	care_development	2	70
201	4	care_development	2	8
202	4	care_development	2	8
203	4	care_development	2	2
204	4	care_development	2	2
205	4	research	4	5
206	4	research	4	4
207	4	research	4	4
208	4	research	4	5
209	4	research	4	1
210	4	research	4	1
211	4	research	4	1
212	4	research	4	1
213	4	research	4	1
214	4	research	4	1
215	4	research	4	1
216	4	research	4	1
217	4	research	4	1
218	4	research	4	1
219	4	research	4	2
220	4	research	4	6
221	4	research	4	4
222	4	research	4	1
223	4	research	4	1
224	4	research	4	1
225	4	research	4	1
226	4	research	4	1
227	5	learning_seminars	1	4
228	5	learning_seminars	1	6
229	5	learning_seminars	1	5
230	5	care_development	2	1
231	5	team_development	2	10
232	5	team_development	2	10
233	5	team_development	2	1
234	5	team_development	2	1
235	5	care_development	2	25
236	5	team_development	2	25
237	5	care_development	2	25
238	5	care_development	2	8
239	5	care_development	2	8
240	5	care_development	2	8
241	5	care_development	2	8
242	5	care_development	2	8
243	5	care_development	2	8
244	5	care_development	2	25
245	5	care_development	2	1
246	5	care_development	2	25
247	5	care_development	2	4
248	5	external_communication	4	7
249	5	external_communication	4	2

For peer review only

250	5	external_communication	4	1
251	5	external_communication	4	3
252	5	care_development	2	7
253	5	care_development	2	20
254	5	care_development	2	20
255	5	care_development	2	20
256	5	care_development		
257	5	care_development		
258	5	care_development		
259	5	care_development		
260	5	care_development		
261	5	care_development		
262	5	care_development		
263	5	care_development		
264	5	care_development		
265	5	care_development		
266	5	care_development		
267	5	care_development		
268	5	care_development		
269	5	care_development		
270	5	research	4	3
271	5	research	4	3
272	5	research	4	3
273	5	research	4	1
274	5	research	4	1
275	5	research	4	1
276	5	research	4	1
277	5	research	4	1
278	5	research	4	1
279	5	research	4	1
280	5	research	4	1
281	5	research	4	1
282	5	research	4	4
283	5	research	4	1
284	6	learning_seminars	1	9
285	6	learning_seminars	1	9
286	6	learning_seminars	1	1
287	6	care_development	2	8
288	6	care_development	2	14
289	6	care_development	2	7
290	6	care_development	2	2
291	6	care_development	2	8
292	6	care_development	2	8
293	6	care_development	2	8
294	6	care_development	2	10
295	6	care_development	2	10
296	6	care_development	2	10
297	6	care_development	2	10
298	6	care_development	2	20
299	6	care_development	2	2

For peer review only

300	6	care_development	2	2
301	6	care_development	2	2
302	6	care_development	2	2
303	6	care_development	2	2
304	6	care_development	2	2
305	6	care_development	2	2
306	6	care_development	2	2
307	6	care_development	2	2
308	6	care_development	2	2
309	6	care_development	2	2
310	6	care_development	2	2
311	6	care_development	2	2
312	6	care_development	2	2
313	6	research	4	3
314	6	research	4	2
315	6	research	4	1
316	6	research	4	1
317	6	research	4	1
318	6	research	4	1
319	6	research	4	1
320	6	research	4	1
321	6	research	4	2
322	6	research	4	1
323	6	research	4	3
324	6	research	4	1
325	6	research	4	1
326	6	care_development	2	7
327	7	learning_seminars	1	15
328	7	learning_seminars	1	10
329	7	learning_seminars	1	11
330	7	preparatory_work	1	2
331	7	learning_seminars	1	10
332	7	preparatory_work	1	1
333	7	preparatory_work	1	2
334	7	research	4	5
335	7	internal_communication	1	15
336	7	preparatory_work	1	3
337	7	educational_activities	1	10
338	7	preparatory_work	1	2
339	7	external_communication	4	2
340	7	preparatory_work	1	5
341	7	educational_activities	1	10
342	7	educational_activities	1	10
343	7	external_communication	1	3
344	7	external_communication	1	2
345	7	learning_seminars	1	10
346	7	educational_activities	1	10
347	7	preparatory_work	1	5
348	7	preparatory_work	1	3
349	7	internal_communication	1	3

For peer review only

350	7 research	4	4
351	7 internal_communication	1	3
352	7 internal_communication	1	7
353	7 preparatory_work	1	4
354	7 administration	2	2
355	7 external_communication	4	2
356	7 preparatory_work	1	4
357	7 preparatory_work	1	8
358	7 preparatory_work	1	5
359	7 reporting	2	1
360	7 reporting	2	1
361	7 preparatory_work	1	2
362	7 preparatory_work	1	2
363	7 preparatory_work	1	5
364	7 external_communication	4	1
365	7 preparatory_work	1	3
366	7 internal_communication	1	1
367	7 reporting	2	3
368	7 preparatory_work	1	3
369	7 reporting	2	1
370	7 preparatory_work	1	1
371	7 reporting	2	1
372	7 reporting	2	1
373	7 preparatory_work	1	2
374	7 preparatory_work	1	2
375	7 external_communication	4	1
376	7 reporting	2	1
377	7 preparatory_work	1	3
378	7 preparatory_work	1	2
379	7 preparatory_work	1	3
380	7 preparatory_work	1	2
381	7 research	4	4
382	7 educational_activities	1	2
383	7 preparatory_work	1	3
384	7 external_communication	4	2
385	7 external_communication	4	2
386	7 preparatory_work	1	2
387	7 preparatory_work	1	4
388	7 preparatory_work	1	2
389	7 preparatory_work	1	2
390	7 external_communication	4	1
391	7 internal_communication	1	2
392	7 preparatory_work	1	1
393	7 preparatory_work	1	4
394	7 research	4	1
395	7 preparatory_work	1	3
396	7 preparatory_work	1	5
397	7 preparatory_work	1	3
398	7 preparatory_work	1	4
399	7 reporting	2	2

For peer review only

400	7	preparatory_work	1	2
401	7	preparatory_work	1	2
402	7	preparatory_work	1	2
403	7	preparatory_work	1	1
404	7	preparatory_work	1	1
405	7	preparatory_work	1	12
406	7	research	4	4
407	7	preparatory_work	1	1
408	7	internal_communication	1	3
409	7	preparatory_work	1	3
410	7	reporting	2	2
411	7	internal_communication	1	2
412	7	preparatory_work	1	3
413	7	preparatory_work	1	2
414	7	internal_communication	1	2
415	7	preparatory_work	1	3
416	7	preparatory_work	1	2
417	7	preparatory_work	1	1
418	7	preparatory_work	1	2
419	7	preparatory_work	1	1
420	7	preparatory_work	1	1
421	7	preparatory_work	1	2
422	7	learning_seminars	1	10
423	7	preparatory_work	1	4
424	7	research	4	4
425	7	preparatory_work	1	1
426	7	preparatory_work	1	3
427	7	research	4	4
428	7	preparatory_work	1	1
429	7	preparatory_work	1	3
430	7	preparatory_work	1	10
431	7	educational_activities	1	3
432	7	preparatory_work	1	1
433	7	internal_communication	1	12
434	7	external_communication	4	2
435	7	external_communication	4	2
436	7	preparatory_work	1	3
437	7	preparatory_work	1	3
438	7	preparatory_work	1	1
439	7	preparatory_work	1	2
440	7	preparatory_work	1	4
441	7	research	4	4
442	7	preparatory_work	1	1
443	7	preparatory_work	1	1
444	7	internal_communication	1	3
445	7	preparatory_work	1	2
446	7	preparatory_work	1	1
447	7	learning_seminars	1	10
448	7	preparatory_work	1	3
449	7	educational_activities	1	3

For peer review only

450	7	preparatory_work	1	1
451	7	preparatory_work	1	4
452	7	preparatory_work	1	2
453	7	preparatory_work	1	1
454	7	preparatory_work	1	2
455	7	preparatory_work	1	2
456	7	preparatory_work	1	2
457	7	preparatory_work	1	1
458	7	preparatory_work	1	1
459	7	preparatory_work	1	3
460	7	preparatory_work	1	4
461	7	educational_activities	1	2
462	7	preparatory_work	1	3
463	7	educational_activities	1	2
464	7	preparatory_work	1	2
465	7	preparatory_work	1	3
466	7	preparatory_work	1	1
467	7	preparatory_work	1	3
468	7	learning_seminars	1	10
469	7	preparatory_work	1	1
470	7	educational_activities	1	10
471	7	research	4	3
472	7	preparatory_work	1	4
473	7	preparatory_work	1	5
474	7	preparatory_work	1	5
475	7	preparatory_work	1	4
476	7	preparatory_work	1	1
477	7	administration	2	1
478	7	preparatory_work	1	2
479	7	preparatory_work	1	3
480	7	educational_activities	1	3
481	7	preparatory_work	1	2
482	7	preparatory_work	1	5
483	7	preparatory_work	1	5
484	7	preparatory_work	1	3
485	7	educational_activities	1	10
486	7	educational_activities	1	15
487	7	educational_activities	1	3
488	7	preparatory_work	1	5
489	7	external_communication	4	1
490	7	preparatory_work	1	3
491	7	educational_activities	1	3
492	7	preparatory_work	1	6
493	7	preparatory_work	1	5
494	7	preparatory_work	1	3
495	7	preparatory_work	1	3
496	7	external_communication	4	5
497	7	preparatory_work	1	2
498	7	preparatory_work	1	1
499	7	preparatory_work	1	3

500	7	research	4	5
501	7	preparatory_work	1	2
502	7	preparatory_work	1	1
503	7	preparatory_work	1	6
504	7	preparatory_work	1	3
505	7	preparatory_work	1	1
506	7	preparatory_work	1	5
507	7	preparatory_work	1	3
508	7	internal_communication	1	7
509	7	preparatory_work	1	4
510	7	preparatory_work	1	4
511	7	preparatory_work	1	3
512	7	preparatory_work	1	2
513	7	preparatory_work	1	2
514	7	educational_activities	1	3
515	7	preparatory_work	1	5
516	7	reporting	2	6
517	7	preparatory_work	1	2
518	7	preparatory_work	1	2
519	7	educational_activities	1	7
520	7	preparatory_work	1	2
521	7	preparatory_work	1	2
522	7	preparatory_work	1	1
523	7	research	4	2
524	7	learning_seminars	1	10
525	7	preparatory_work	1	3
526	7	preparatory_work	1	3
527	7	internal_communication	1	1
528	7	preparatory_work	1	2
529	7	preparatory_work	1	3
530	7	preparatory_work	1	3
531	7	preparatory_work	1	3
532	7	research	4	2
533	7	research	4	2
534	7	research	4	1

For peer review only

Time_minutes	Staff_cat
2940	HCP
2940	HCP
2940	HCP
60	managers
960	HCP
9600	HCP
2400	HCP
50000	managers
2400	HCP
1200	HCP
1800	HCP
4800	HCP
300	HCP
4200	HCP
144.55	HCP
201.866666666667	HCP
262.583333333333	HCP
300.833333333333	HCP
143.7	HCP
245.083333333333	HCP
261.583333333333	HCP
45.1	HCP
51.1	HCP
32.9666666666667	HCP
43.6666666666667	HCP
89.5666666666667	HCP
115.233333333333	HCP
193.933333333333	HCP
120.766666666667	HCP
31.0833333333333	managers
33.7166666666667	managers
3360	HCP
4800	HCP
3780	HCP
3780	HCP
2400	managers
600	managers
12000	HCP
10000	HCP
30	managers
3600	HCP
210	HCP
600	managers
960	HCP
1920	HCP
5760	HCP
400	HCP
240	managers
1920	HCP

For peer review only

2430	managers
180	HCP
7440	managers
1080	managers
120	managers
300	HCP
6000	HCP
2500	HCP
1500	HCP
302.1	HCP
232.466666666667	HCP
210.666666666667	HCP
380.333333333333	HCP
268.6	HCP
206.8	HCP
33.4666666666667	HCP
41.5333333333333	HCP
31.5666666666667	HCP
76.8166666666667	HCP
170.266666666667	HCP
126.533333333333	HCP
33.3833333333333	managers
30.5833333333333	managers
1260	HCP
2100	HCP
270	HCP
720	HCP
250	HCP
240	HCP
2940	HCP
630	HCP
450	HCP
3360	HCP
1680	HCP
120	HCP
720	HCP
120	change_agents
50	change_agents
300	HCP
500	change_agents
45	change_agents
60	managers
720	HCP
540	HCP
30	change_agents
840	HCP
1680	HCP
300	HCP
90	change_agents
135	change_agents

For peer review only

90	change_agents
840	HCP
300	change_agents
120	change_agents
8400	HCP
360	HCP
270	HCP
7200	HCP
2100	HCP
2520	HCP
420	HCP
420	HCP
45	change_agents
360	HCP
200	HCP
40	change_agents
420	HCP
240	HCP
120	change_agents
45	change_agents
90	HCP
120	HCP
3780	HCP
240	HCP
30	change_agents
300	change_agents
420	HCP
300	HCP
360	HCP
450	HCP
2100	HCP
240	managers
300	HCP
5400	HCP
2520	HCP
2520	HCP
360	HCP
1680	HCP
1680	HCP
180	managers
2100	HCP
1680	HCP
1680	HCP
240	managers
300	managers
840	HCP
135.2	HCP
249.083333333333	HCP
328.533333333333	HCP
335	HCP

For peer review only

274.916666666667	HCP
225.833333333333	HCP
223	HCP
47.2333333333333	HCP
69.5	HCP
38.4666666666667	HCP
35.5833333333333	HCP
42.5833333333333	HCP
29.9	HCP
44.8666666666667	HCP
191.916666666667	HCP
374.5	HCP
389	HCP
31.1166666666667	managers
34.7833333333333	managers
4320	HCP
4320	HCP
120	HCP
240	HCP
60	HCP
240	HCP
120	HCP
16800	HCP
16800	HCP
3360	HCP
120	HCP
840	HCP
6300	HCP
60	HCP
120	HCP
60	HCP
1320	HCP
720	HCP
660	HCP
10800	HCP
660	HCP
120	HCP
240	HCP
360	managers
4620	HCP
600	HCP
720	HCP
4320	HCP
3840	HCP
3840	HCP
4320	HCP
4320	HCP
4320	HCP
16800	HCP
360	HCP

For peer review only

4200	HCP
3360	HCP
3360	HCP
840	HCP
840	HCP
293.25	HCP
198.4	HCP
229.866666666667	HCP
225.916666666667	HCP
47.2666666666667	HCP
41.2833333333333	HCP
33.0666666666667	HCP
36.2	HCP
41.95	HCP
51.9166666666667	HCP
43.9166666666667	HCP
37.9	HCP
42.0166666666667	HCP
39.05	HCP
45.6	HCP
590.6	HCP
238.333333333333	HCP
24.2333333333333	managers
26.3	managers
37.8	managers
20.65	managers
27.8166666666667	HCP
1920	HCP
2880	HCP
2400	HCP
2400	managers
51000	HCP
25500	HCP
4680	managers
4680	managers
1500	HCP
2100	HCP
1500	HCP
480	HCP
720	HCP
720	HCP
720	HCP
720	HCP
480	HCP
1500	HCP
2400	change_agents
4500	HCP
3360	HCP
2400	HCP
960	HCP

For peer review only

	720	managers
	720	HCP
	105	HCP
	1200	HCP
	1800	HCP
	1200	HCP
		HCP
		HCP
		HCP
		HCP
		HCP
		HCP
		HCP
		HCP
		HCP
		HCP
		HCP
		HCP
		HCP
		HCP
122.9		HCP
171.5		HCP
157.85		HCP
26.2166666666667		HCP
48.8166666666667		HCP
39.6666666666667		HCP
25.9833333333333		HCP
38.9166666666667		HCP
52.9		HCP
26.9833333333333		HCP
32.7		HCP
33.8166666666667		HCP
218.8		HCP
38.4		managers
	4320	HCP
	4320	HCP
	480	HCP
	1260	HCP
	840	HCP
	4200	HCP
	1920	HCP
	960	HCP
	480	HCP
	480	HCP
	600	HCP
	600	HCP
	800	HCP
	600	HCP
	6000	HCP
	3360	HCP

For peer review only

480	HCP
240	HCP
480	HCP
60	HCP
480	HCP
480	HCP
240	HCP
240	HCP
120	HCP
120	HCP
8640	HCP
960	HCP
960	HCP
147	HCP
76.7666666666667	HCP
40.6166666666667	HCP
37.4666666666667	HCP
51.7333333333333	HCP
45.3333333333333	HCP
19.8833333333333	HCP
21.8	HCP
63.3	HCP
33.8166666666667	HCP
121.9	HCP
46.1	managers
28.2833333333333	managers
105	HCP
6300	change_agents
4200	change_agents
4620	change_agents
480	change_agents
4800	change_agents
180	change_agents
180	change_agents
300	change_agents
450	change_agents
135	change_agents
2400	change_agents
60	change_agents
60	change_agents
300	change_agents
1200	managers
1500	managers
180	change_agents
120	change_agents
5400	managers
600	change_agents
150	change_agents
360	change_agents
180	managers

For peer review only

720	change_agents
180	change_agents
840	change_agents
120	change_agents
480	change_agents
80	change_agents
240	change_agents
720	change_agents
2250	change_agents
240	change_agents
240	change_agents
120	managers
360	managers
900	change_agents
120	change_agents
360	change_agents
60	change_agents
360	change_agents
360	change_agents
60	change_agents
120	change_agents
60	change_agents
120	change_agents
120	change_agents
120	change_agents
60	change_agents
120	change_agents
360	change_agents
240	change_agents
540	change_agents
120	change_agents
480	change_agents
240	change_agents
360	change_agents
960	change_agents
960	change_agents
120	change_agents
480	change_agents
180	change_agents
180	change_agents
30	change_agents
180	change_agents
15	change_agents
240	change_agents
240	change_agents
360	change_agents
600	change_agents
360	change_agents
480	managers
60	change_agents

For peer review only

60	managers
30	managers
60	change_agents
150	change_agents
150	change_agents
360	change_agents
360	change_agents
90	change_agents
180	change_agents
180	change_agents
60	change_agents
120	change_agents
270	change_agents
120	change_agents
60	change_agents
180	change_agents
240	change_agents
30	change_agents
240	change_agents
60	change_agents
300	change_agents
120	change_agents
4800	change_agents
240	managers
240	change_agents
180	change_agents
180	change_agents
360	change_agents
90	change_agents
180	change_agents
600	change_agents
540	change_agents
180	change_agents
360	change_agents
960	change_agents
960	change_agents
180	change_agents
180	change_agents
120	change_agents
180	change_agents
720	change_agents
360	change_agents
60	change_agents
60	change_agents
360	change_agents
480	change_agents
240	change_agents
4800	change_agents
360	change_agents
1440	change_agents

For peer review only

60	change_agents
960	change_agents
60	change_agents
60	change_agents
240	change_agents
60	change_agents
480	change_agents
90	change_agents
60	change_agents
180	change_agents
480	change_agents
960	managers
1080	change_agents
960	managers
360	change_agents
180	change_agents
60	change_agents
360	change_agents
4800	change_agents
180	change_agents
4200	change_agents
540	change_agents
480	change_agents
600	change_agents
600	change_agents
480	change_agents
360	change_agents
240	change_agents
180	change_agents
540	change_agents
900	change_agents
240	change_agents
600	change_agents
600	change_agents
300	change_agents
2100	change_agents
2700	change_agents
900	managers
300	change_agents
60	change_agents
540	change_agents
270	change_agents
720	change_agents
300	change_agents
720	change_agents
810	change_agents
3000	change_agents
480	change_agents
60	change_agents
540	change_agents

For peer review only

2250	change_agents
120	change_agents
150	change_agents
1620	managers
180	managers
120	change_agents
450	change_agents
720	change_agents
630	change_agents
120	change_agents
120	change_agents
360	change_agents
180	change_agents
120	change_agents
540	managers
750	change_agents
360	managers
240	change_agents
600	change_agents
840	managers
30	change_agents
120	change_agents
120	change_agents
120	change_agents
4200	managers
360	change_agents
720	change_agents
120	change_agents
240	change_agents
360	change_agents
270	change_agents
540	change_agents
198.866666666667	change_agents
104.966666666667	change_agents
35.95	change_agents

For peer review only

Geriatric rehabilitation

Unit 1

Year	Month	Length of stay, mean
2016	January	31.5
2016	February	28.6
2016	March	22.0
2016	April	22.8
2016	May	31.9
2016	June	26.1
2016	July	39.3
2016	August	24.3
2016	September	34.6
2016	October	30.0
2016	November	34.0
2016	December	28.1
2017	January	26.2
2017	February	25.1
2017	March	23.3
2017	April	25.1
2017	May	26.9
2017	June	29.5
2017	July	32.5
2017	August	28.2
2017	September	22.2
2017	October	27.7
2017	November	20.3
2017	December	29.2
2018	January	29.3
2018	February	19.3
2018	March	32.6
2018	April	28.1
2018	May	29.8
2018	June	29.7
2018	July	24.4
2018	August	39.4
2018	September	22.5
2018	October	28.0
2018	November	22.7
2018	December	19.9
2019	January	29.5
2019	February	18.9
2019	March	23.6
2019	April	37.2
2019	May	28.5
2019	June	22.8
2019	July	19.0
2019	August	21.1
2019	September	26.0
2019	October	25.0
2019	November	26.9

For peer review only

Unit 2

Year	Number of discharges
2016	691
2017	706
2018	675
2019	710

Psychiatrics

Unit 1 and unit 2, combined (aggregated data)

Year	Length of stay, mean ±SD	Time to readmissions, mean ±SD
2016	15.5 ±16.4	219.3 ±277.6
2017	15.2 ±15.0	160.2 ±180.8
2018	14.6 ±23.7	79.7 ±88.3
2019	13.9 ±14.1	28.1 ±29.8

For peer review only

For peer review only

±

For peer review only

Online-Only Supplements

Health economics of implementing a more person-centred care approach: the IMPROVE project

Authors

Hanna Gyllensten[1,2] Associate professor

Malin Tistad[3,4] Associate professor

Helena Fridberg[3] PhD

Lars Wallin[1,2,3] Professor

Author affiliations

[1] Institute of Health and Care Sciences, University of Gothenburg, Box 457, SE-405 30 Gothenburg, Sweden.

[2] University of Gothenburg Centre for Person-Centred Care (GPCC), Sahlgrenska Academy, University of Gothenburg, Sweden.

[3] School of Health and Welfare, Dalarna University, Falun, Sweden.

[4] Department of Neurobiology, Care Sciences and Society, Karolinska Institutet, Stockholm, Sweden.

Content

Authors 1

Author affiliations 1

STable 1: Resource use reported in logbooks by type of activity and organisational units. 2

SFigure 1: Length of stay at the geriatric unit (unit 1) and trend over time. 3

SFigure 2: Length of stay at the psychiatric units (combined) and trend over time. 4

SFigure 3: Time to readmission at the psychiatric units (combined) and trend over time. 5

For peer review only

STable 1: Resource use reported in logbooks by type of activity and organisational units.

Activities by cost categories[a]	Organisational unit							
	Geriatric unit 1 working days[b]	Geriatric unit 2 working days[b]	Nephrology unit working days[b]	Primary care unit working days[b]	Psychiatric unit 1 working days[b]	Psychiatric unit 2 working days[b]	DD working days[b]	Total working days[b]
Implementation strategies								
Documentation	0	0	0	1	0	0	0	1
Educational activities	0	0	0	0	0	0	46	46
External communication	0	0	0	0	0	0	1	1
Internal communication	0	0	0	0	0	0	8	8
Learning seminars	18	25	57	42	15	19	92	268
Preparatory work	1	70	11	2	0	0	88	171
Workplace meetings	0	0	1	0	0	0	0	1
Service delivery								
Administration	0	0	0	0	0	0	2	2
Care development	113	41	32	35	53	74	0	348
Documentation	0	0	0	1	0	0	0	1
Educational activities	26	4	37	105	0	0	0	172
IT support	0	0	0	0	0	0	0	0
Reporting	0	0	0	0	0	0	4	4
Team development	23	22	5	0	183	0	0	233
Workplace meetings	0	0	0	71	0	0	0	71
Research/development								
External communication	0	0	11	11	10	0	15	48
Preparatory work	0	0	0	1	0	0	0	1
Research	5	4	9	7	2	2	13	42
Total	185	167	162	275	263	95	267	1414

[a] Developed from previously reported cost categories in implementation programmes: 1) costs for executing implementation strategies, 2) excess costs for service delivery as implementation/service changes, 3) opportunity costs to providers and patients, and 4) research/development costs (35). No account was taken for changes in costs for direct patient care (category 3).

[b] Assuming 8-hour working days.

Abbreviations: DD = Department for development.

For peer review only

SFigure 1: Length of stay at the geriatric unit (unit 1) and trend over time.

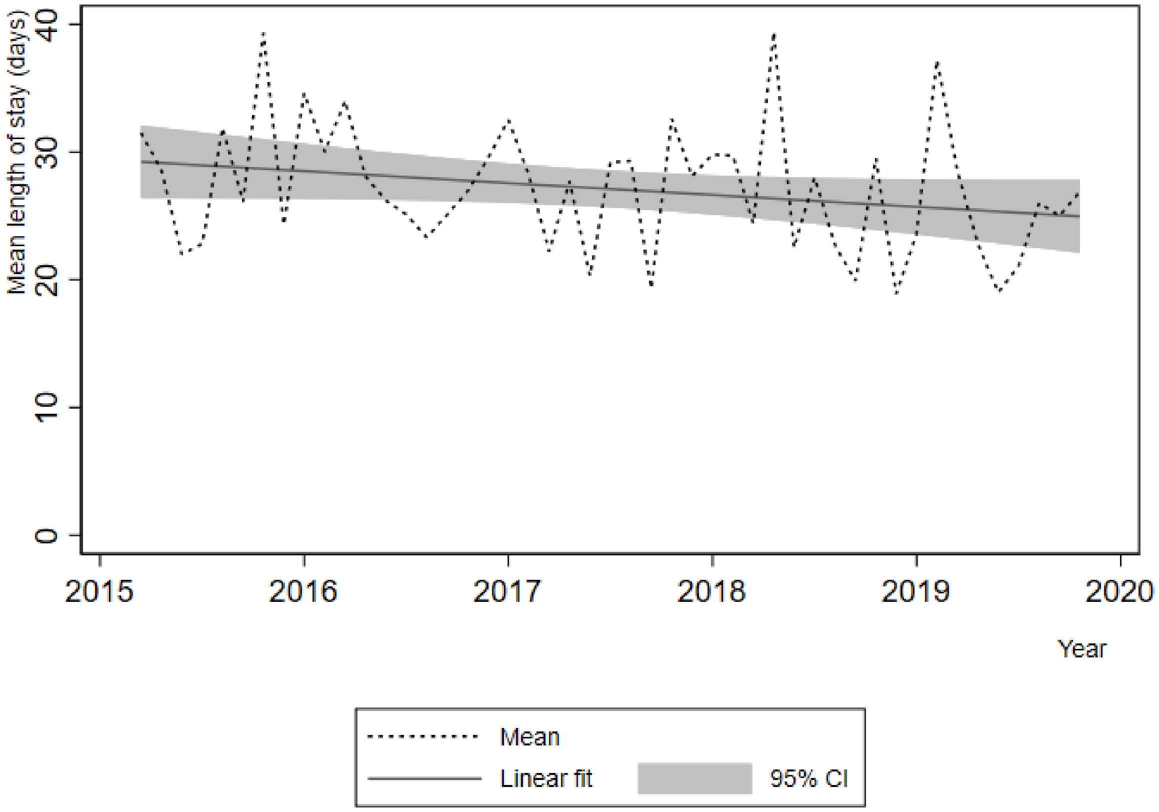

SFigure 2: Length of stay at the psychiatric units (combined) and trend over time.

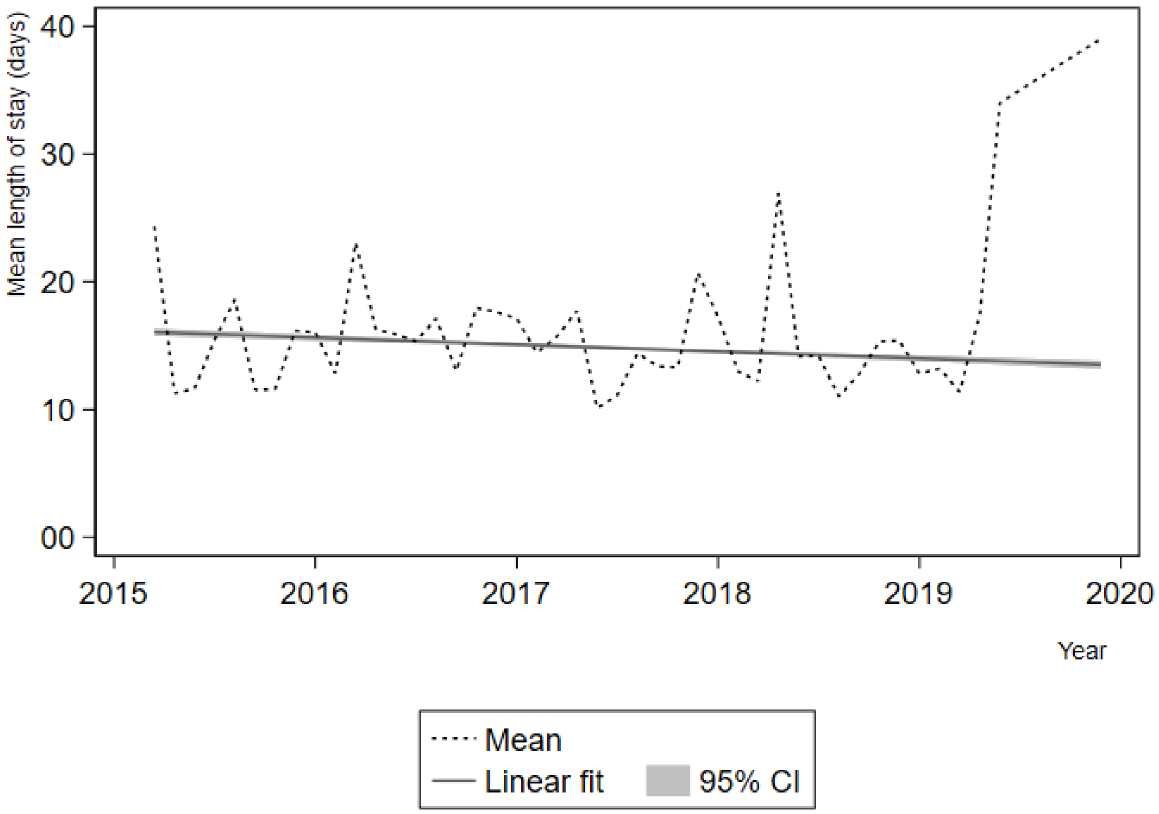

Observe that the 95% confidence interval is not visible (overlaid by the linear equation line) due to large sample size.

review only

SFigure 3: Time to readmission at the psychiatric units (combined) and trend over time.

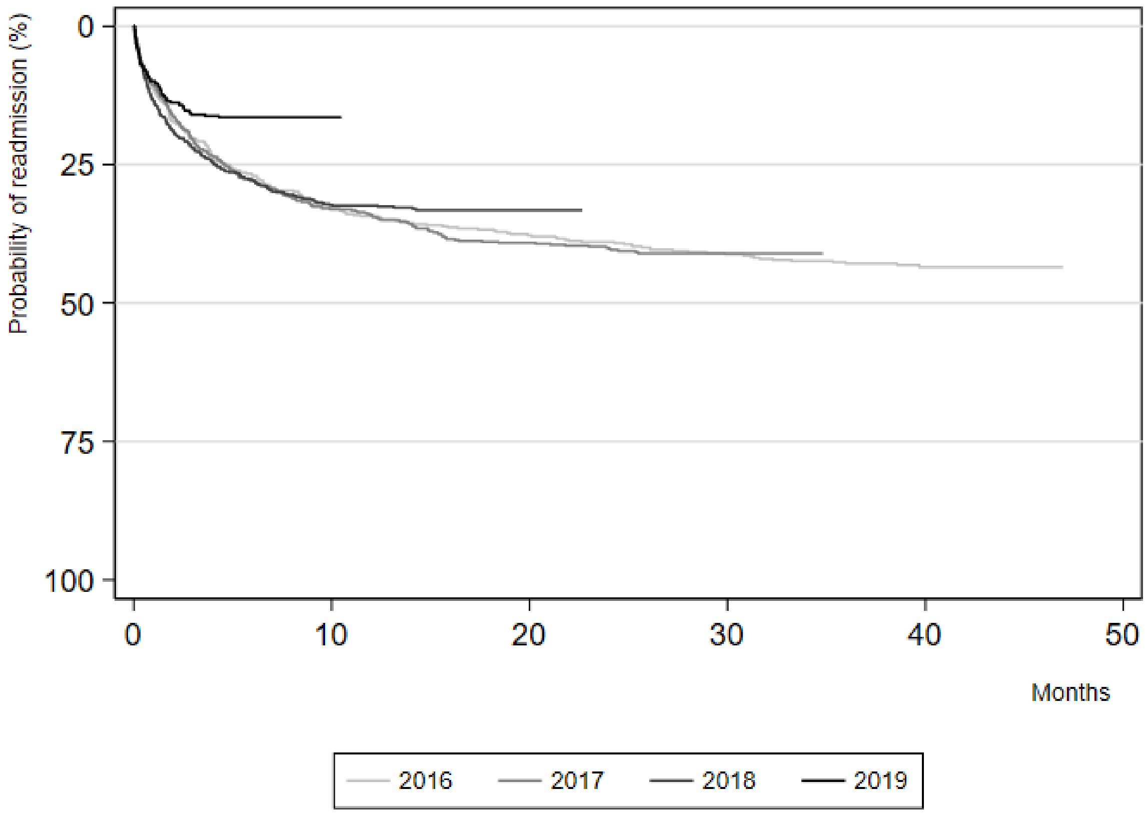

review only

CHEERS 2022 Checklist

Topic	No.	Item	Location where item is reported
**Title**			
	1	Identify the study as an economic evaluation and specify the interventions being compared.	Page 1
**Abstract**			
	2	Provide a structured summary that highlights context, key methods, results, and alternative analyses.	Page 2
**Introduction**			
**Background and objectives**	3	Give the context for the study, the study question, and its practical relevance for decision making in policy or practice.	Page 4
**Methods**			
**Health economic analysis plan**	4	Indicate whether a health economic analysis plan was developed and where available.	Supplement S1
**Study population**	5	Describe characteristics of the study population (such as age range, demographics, socioeconomic, or clinical characteristics).	N/A (staff only)
**Setting and location**	6	Provide relevant contextual information that may influence findings.	Pages 5-6
**Comparators**	7	Describe the interventions or strategies being compared and why chosen.	Page 6
**Perspective**	8	State the perspective(s) adopted by the study and why chosen.	Pages 9-10
**Time horizon**	9	State the time horizon for the study and why appropriate.	N/A
**Discount rate**	10	Report the discount rate(s) and reason chosen.	N/A
**Selection of outcomes**	11	Describe what outcomes were used as the measure(s) of benefit(s) and harm(s).	N/A
**Measurement of outcomes**	12	Describe how outcomes used to capture benefit(s) and harm(s) were measured.	N/A

Topic	No.	Item	Location where item is reported
**Valuation of outcomes**	13	Describe the population and methods used to measure and value outcomes.	N/A
**Measurement and valuation of resources and costs**	14	Describe how costs were valued.	Pages 9-10
**Currency, price date, and conversion**	15	Report the dates of the estimated resource quantities and unit costs, plus the currency and year of conversion.	Page 10
**Rationale and description of model**	16	If modelling is used, describe in detail and why used. Report if the model is publicly available and where it can be accessed.	N/A
**Analytics and assumptions**	17	Describe any methods for analysing or statistically transforming data, any extrapolation methods, and approaches for validating any model used.	Page 10
**Characterising heterogeneity**	18	Describe any methods used for estimating how the results of the study vary for subgroups.	N/A
**Characterising distributional effects**	19	Describe how impacts are distributed across different individuals or adjustments made to reflect priority populations.	N/A
**Characterising uncertainty**	20	Describe methods to characterise any sources of uncertainty in the analysis.	N/A
**Approach to engagement with patients and others affected by the study**	21	Describe any approaches to engage patients or service recipients, the general public, communities, or stakeholders (such as clinicians or payers) in the design of the study.	Pages 8 and 16 (staff)<br>No patient/public
**Results**			
**Study parameters**	22	Report all analytic inputs (such as values, ranges, references) including uncertainty or distributional assumptions.	Table 3
**Summary of main results**	23	Report the mean values for the main categories of costs and outcomes of interest and summarise them in the most appropriate overall measure.	Table 2
**Effect of uncertainty**	24	Describe how uncertainty about analytic judgments, inputs, or projections affect findings. Report the effect of choice of discount rate and time horizon, if applicable.	N/A

For peer review only - http://bmjopen.bmj.com/site/about/guidelines.xhtml

Topic	No.	Item	Location where item is reported
**Effect of engagement with patients and others affected by the study**	25	Report on any difference patient/service recipient, general public, community, or stakeholder involvement made to the approach or findings of the study	N/A
**Discussion**			
**Study findings, limitations, generalisability, and current knowledge**	26	Report key findings, limitations, ethical or equity considerations not captured, and how these could affect patients, policy, or practice.	Page 16
**Other relevant information**			
**Source of funding**	27	Describe how the study was funded and any role of the funder in the identification, design, conduct, and reporting of the analysis	Page 21
**Conflicts of interest**	28	Report authors conflicts of interest according to journal or International Committee of Medical Journal Editors requirements.	Page 21

From: Husereau D, Drummond M, Augustovski F, et al. Consolidated Health Economic Evaluation Reporting Standards 2022 (CHEERS 2022) Explanation and Elaboration: A Report of the ISPOR CHEERS II Good Practices Task Force. Value Health 2022;25. doi:10.1016/j.jval.2021.10.008

For peer review only

BMJ Open

An analysis on personnel costs and working time for implementing a more person-centred care approach: A case study with embedded units in a Swedish region

Journal:	*BMJ Open*
Manuscript ID	bmjopen-2023-073829.R1
Article Type:	Original research
Date Submitted by the Author:	18-Aug-2023
Complete List of Authors:	Gyllensten, Hanna; University of Gothenburg, Institute of Health and Care Sciences; University of Gothenburg Centre for Person-Centred Care<br>Tistad, Malin; Dalarna University, School of Health and Welfare; Karolinska Institute, Department of Neurobiology, Care Sciences and Society<br>Fridberg, Helena; Dalarna University, School of Health and Welfare<br>Wallin, Lars; Dalarna University, School of health and welfare; University of Gothenburg, Institute of Health and Care Sciences
**Primary Subject Heading**:	Health economics
Secondary Subject Heading:	Health services research, Nursing, Patient-centred medicine
Keywords:	HEALTH ECONOMICS, Health economics < HEALTH SERVICES ADMINISTRATION & MANAGEMENT, Organisational development < HEALTH SERVICES ADMINISTRATION & MANAGEMENT, Patient-Centered Care

SCHOLARONE™
Manuscripts

I, the Submitting Author has the right to grant and does grant on behalf of all authors of the Work (as defined in the below author licence), an exclusive licence and/or a non-exclusive licence for contributions from authors who are: i) UK Crown employees; ii) where BMJ has agreed a CC-BY licence shall apply, and/or iii) in accordance with the terms applicable for US Federal Government officers or employees acting as part of their official duties; on a worldwide, perpetual, irrevocable, royalty-free basis to BMJ Publishing Group Ltd ("BMJ") its licensees and where the relevant Journal is co-owned by BMJ to the co-owners of the Journal, to publish the Work in this journal and any other BMJ products and to exploit all rights, as set out in our licence.

The Submitting Author accepts and understands that any supply made under these terms is made by BMJ to the Submitting Author unless you are acting as an employee on behalf of your employer or a postgraduate student of an affiliated institution which is paying any applicable article publishing charge ("APC") for Open Access articles. Where the Submitting Author wishes to make the Work available on an Open Access basis (and intends to pay the relevant APC), the terms of reuse of such Open Access shall be governed by a Creative Commons licence – details of these licences and which Creative Commons licence will apply to this Work are set out in our licence referred to above.

Other than as permitted in any relevant BMJ Author's Self Archiving Policies, I confirm this Work has not been accepted for publication elsewhere, is not being considered for publication elsewhere and does not duplicate material already published. I confirm all authors consent to publication of this Work and authorise the granting of this licence.

For peer review only

An analysis on personnel costs and working time for implementing a more person-centred care approach: A case study with embedded units in a Swedish region

Authors

Hanna Gyllensten[1,2] Associate professor

Malin Tistad[3,4] Associate professor

Helena Fridberg[3] PhD

Lars Wallin[1,2,3] Professor

Author affiliations

[1] Institute of Health and Care Sciences, University of Gothenburg, Box 457, SE-405 30 Gothenburg, Sweden.

[2] University of Gothenburg Centre for Person-Centred Care (GPCC), Sahlgrenska Academy, University of Gothenburg, Sweden.

[3] School of Health and Welfare, Dalarna University, Falun, Sweden.

[4] Department of Neurobiology, Care Sciences and Society, Karolinska Institutet, Stockholm, Sweden.

Corresponding author

Hanna Gyllensten, Institute of Health and Care Sciences, Sahlgrenska Academy, University of Gothenburg, Postal Address: Box 457, SE-405 30 Gothenburg, Sweden, E-mail: hanna.gyllensten@gu.se. Phone: +46-(0)70-748 24 12

Key words

Person-centred care; Person-centered care; Costs and cost analysis; Implementation science.

For peer review only

Word count (excluding title page, abstract, references, figures and tables): 5080

For peer review only - http://bmjopen.bmj.com/site/about/guidelines.xhtml

Abstract

Objectives: Our aim was to describe the time and costs used during the implementation of a more person-centred care (PCC) approach as part of ordinary practice.

Design: A case study with embedded units.

Setting: Region Dalarna, Sweden.

Participants: The Department for Development (DD) staff who provided a central support function in the implementation and six healthcare units: nephrology, two geriatric care and rehabilitation units, two psychiatry units, and primary care.

Interventions: More PCC.

Primary and secondary outcome measures: Working days and related salary costs reported by categories indicating costs for implementation strategies, service delivery, and research/development costs.

Results: The health care units logged on average 5.5 working days per staff member. In the healthcare units, 6-57% of the time reported was used for implementation strategies, 40-90% for service delivery, and 2-12% for research/development. Of the time reported by the DD, 88% was assigned to implementation strategies. Costs associated with reported time indicated 23% of costs for this implementation occurred in the DD. Using the budgeted cost, this proportion increased to 48%. The budget for the DD corresponded to SEK 2.30 per citizen per year and 0.009 % of the total health care budget of the region.

Conclusions: The study found that a large part of resources used for this implementation of more PCC occurred in the DD, although at least half of the costs occurred in the healthcare units. Moreover, the cost of providing a central support function corresponds to a tiny proportion of the total health budget.

For peer review only - http://bmjopen.bmj.com/site/about/guidelines.xhtml

For peer review only

Strengths and limitations of this study

- The study was conducted by an independent research body that included researchers from different disciplines.
- The included healthcare units were given multiple opportunities to clarify and correct their records.
- The staff had no time set off for the logbooks and thus often filled in the logbooks retroactively, potentially resulting in recall bias.
- Most of the observed changes were due to other factors than the implementation of a more PCC, due to parallel changes in these units.

For peer review only

For peer review only - http://bmjopen.bmj.com/site/about/guidelines.xhtml

Introduction

Healthcare systems in many countries experience increasing economic demands, both through the development of new technologies and treatments and through a changing age distribution in the population resulting in more people with multiple chronic conditions. As in many countries (1,2), legislators and health care organisations in Sweden have put person-centred care (PCC) high on the development agenda (3), and management-control efforts are increasingly aimed at implementing PCC.

PCC acknowledges and endorses every person´s resources, interests, and needs, comprising shared responsibility and power, as well as coordinated care and treatment (4–6). PCC strives towards a meaningful life, which differs from similar concepts (e.g., patient-centred care) that focus on functional life (7). It is related to the integrated people-centred health services promoted by the World Health Organisation (8), albeit without the community perspective embedded in the framework. PCC has been promoted to address patient dissatisfaction with health care access and delivery (9) and as a potentially cost-saving or cost containing measure through more effective use of resources (10). Several studies suggest cost-saving as one argument for PCC (10,11). Thus, PCC is expected to both improve care quality and contain costs (12).

However, the knowledge about costs associated with introducing a more PCC is limited and scattered (13). Some studies have included training costs in intervention costs (14,15) or indicated costs of transferring staff between organizational units (16), but to our knowledge its implementation costs have not been reported. Regardless of the little knowledge of costs associated with its implementation, PCC has a growing impact on the health care industry in many countries (1,2). Overlooking such costs, however, implies that the time added for this implementation is minimal and can be ignored (17), an assumption that to some extent contradicts previous findings that implementation of PCC was associated with increased job strain (18).

For peer review only - http://bmjopen.bmj.com/site/about/guidelines.xhtml

Thus, this study aimed to describe the time and costs used during the implementation of a more PCC approach as part of ordinary practice. As far as we know, the current study is one of a few reporting health economic aspects of a more PCC approach in ordinary practice, i.e., not as part of an intervention study.

Materials and Methods

Region Dalarna decided in 2015 to promote a more PCC approach throughout the health system. The implementation process was initiated and managed by the healthcare organisation Region Dalarna as an essential part of continuous quality improvement. The *Implementing PCC: Process evaluation of strategies, leadership, and health economy* (IMPROVE) project was conducted in parallel by university researchers. The process evaluation focused on the implementation process rather than PCC (the innovation being implemented). This study thus reports on the resources used for implementing a more PCC approach in one Swedish region, using data from a case study with seven embedded units (*study protocol available as Supporting information*). Previous publications from the IMPROVE project have investigated how patients' perception of person-centred care can be measured (19), how the concept of person-centred care was perceived by health care staff (20), how the concept of PCC was operationalized by the units (21), the used implementation strategies (22), and congruence of managers' perceptions and understanding of PCC across organisational levels (23).

Setting

Region Dalarna is situated in the middle of Sweden. The region covers 6% of the Swedish land area and contains 3% of its population. Four hospitals and 25 healthcare clinics provides public healthcare for the region's population (24). The Swedish health system provides universal coverage for all legal residents, and on some premises, also to visitors from other parts of the European Union, asylum seekers, and undocumented immigrants. The health system is divided based on geographic area into 21 regions providing health care services and 290 municipalities providing care for the elderly and

For peer review only - http://bmjopen.bmj.com/site/about/guidelines.xhtml

disabled. Approximately 84% of all health expenditures were tax-based in 2016, with care prioritised based on an ethical platform including the principles of human dignity, needs and solidarity, and cost-effectiveness, in that order (25).

The innovation

The implementation of more PCC was conducted as part of the region's work towards efficient health care practices, in parallel with their "structure and change work" that included projects related to priority setting and resource allocation in the regional healthcare system. The vision was to put equal emphasis on the patient and professional perspective throughout the care process. The approach chosen was based on the Gothenburg model of PCC (5,26), which has been shown in clinical trials to be cost-effective for several care settings and patient groups (10,27–29). The main feature of the model is its focus on the partnership between the patient and the health care provider built during the co-creation of a written health plan (5). It has previously been reported that, although some ambiguity remained in their description, core practices related to all three cornerstones of PCC – creating, safeguarding and documenting the partnership – were identified in all the embedded units (20). Important practices introduced in the units were routines to elucidate the patient narrative during admission or throughout the care pathway, and use of communication techniques such as motivational interviewing.

The implementation process

As part of the implementation process, changes were made to the region's micro, meso, and macro levels. These changes included the commission of staff in the regions Department for Development (time committed to the project corresponding to 80% of a full-time employment), as well as a budget for expenses associated with the implementation. The Department for Development staff (hereafter called the DD) assigned to lead this process were engaged as operational support provided centrally from the region to the included health care units. Among the DD tasks were to organise learning seminars and support the staff at the health care units during the implementation process. The participating health care units chose their representatives to participate in the learning

For peer review only - http://bmjopen.bmj.com/site/about/guidelines.xhtml

seminars. Thereafter, each health care unit was expected to manage its implementation process. There was no joint implementation support programme other than the learning seminars, but the DD could provide support on-demand. The implementation strategies used have previously been described to mainly fall in two clusters, i.e., Train and educate stakeholder, as well as Develop stakeholder interrelationships (22).

The process evaluation

Interventions or programmes directed to changing health care provision, such as the implementation of PCC, are often described as complex interventions (30). Complex interventions are interventions that contain several interacting components. This interaction can include elements tailored to each participant (e.g., patients or health care staff), sometimes with varying outcomes and goals. In this study complexity included differences in the training of participating staff, differences in organisational structures among units, and each unit defining on their own how an increase in PCC would be interpreted and implemented. It has been recommended that research into the use and effects of complex interventions address the complexity involved (31). This can be done through process evaluations that provide an understanding of how the innovation (in this case PCC) is implemented (32). In a conceptual model for implementation research Proctor and colleagues elaborated on a number of implementation outcomes relevant for such an evaluation, including economic aspects of implementation (33).

The process evaluation was conducted six health care units that participated in the first round of learning seminars and the DD. All units consented to participate in the evaluation. The health care units included were specialised in nephrology, geriatric care and rehabilitation, psychiatry, and primary care.

Costs related to the implementation included staff and the DD's costs, the training of staff, and any support provided locally in the health care units. The study examines resources spent on making this change in health care but does not include any control units. Therefore, the study approach is in line

For peer review only - http://bmjopen.bmj.com/site/about/guidelines.xhtml

with an observational study of a natural experiment in a real-world setting; i.e., the research team has no control over the intervention, there is no control condition, and the knowledge and availability of data for evaluation are partial (34).

Data collection

Data for this study were collected through logbooks, including date and type of activity, how much time each activity took (recorded as either minutes, hours, or workdays) and for how many people, as well as information about who was involved. Each unit selected one person in a leading position in the implementation to complete the logbooks, either as each activity was conducted or retrospectively. These persons received the same instruction in how to use the logbooks. However, in order not to influence the choice of strategies we did not give any guidance regarding taxonomies that could have been used to choose or describe the activities carried out to support the implementation. Reporters were encouraged to report short and often reoccurring activities (e.g., discussions about implementation between colleagues during the workday) as weekly estimates. The persons responsible for the logbooks were encouraged to report on a weekly to monthly basis but some had difficulties adhering to this recommendation due to a high workload and were instead encouraged to use their calendars on a half year basis to track their activities. In some instances, representatives from the research group met with the person responsible for the logbook and assisted to fill out the logs.

For peer review only

Information from the logbooks was used to identify which activities were perceived by personnel units to be related to implementing a more PCC approach and estimations of the time used for this implementation. Only time was reported in the logbooks, no equipment or other expenses were tracked.

In addition, units were asked to provide suggestions (hereafter called unit-specific measures) to evaluate the economic impact of implementing a more PCC approach (Table 1), that leaders in these units viewed as important for understanding the changes in practice induced by PCC. Relevant data

For peer review only - http://bmjopen.bmj.com/site/about/guidelines.xhtml

on these proposed outcomes were collected from each unit retrospectively, and the units were asked to comment on any potential time trends in the data.

Data collection was planned to cover a three-year study period (June 2016-May 2019), but logbook data continued into autumn of 2019 for the psychiatric units and the DD due to delays in previously planned activities. Activities conducted before the ethics approval (in 2017) were filled in retrospectively.

For peer review only

For peer review only - http://bmjopen.bmj.com/site/about/guidelines.xhtml

Table 1: Pre-planned unit-specific measures of resource use (Supplementary file S1).

Unit	Planned reporting	
Nephrology /dialysis unit[a]		Distribution between haemodialysis, assisted peritoneal dialysis and unassisted peritoneal dialysis; work hours (adjusted for the number of patients)
Primary care[a]		Number of patients listed; number of visits per patient
Geriatric care and rehabilitation	Unit 1:	Length of stay
	Unit 2:	Work hours[a]; number of patients; work environment follow-up[a]
Psychiatrics[b]	Unit 1:	Length of stay (considering overcrowding)
	Unit 2:	Length of stay and readmissions (considering overcrowding)

[a] Not reported (deviation from planned analyses).

[b] These two units were merged during a large part of the study, and unit-specific measures were thus reported as a combined unit.

Resource use and cost estimation

In analysing implementation programmes, four categories of costs have previously been suggested in the literature (35): 1) costs for executing implementation strategies, 2) excess costs for service delivery as it changes, 3) opportunity costs to providers and patients, and 4) research/development costs. These categories were used retrospectively by the research group to categorize activities reported in the logbooks. In this study, costs for executing implementation strategies are mainly directed towards centrally organised processes (e.g., seminars). In contrast, the costs for service delivery included activities the units used in operationalising PCC in ordinary practice. This study did not reflect foregone opportunity costs in the care of patients because data collection was not designed for patient-level follow-up. However, it needs to be acknowledged that all resource use in the health system can potentially have an opportunity cost related to an alternative use of existing resources. Activities reported in logbooks were first categorised inductively to identify less

For peer review only - http://bmjopen.bmj.com/site/about/guidelines.xhtml

For peer review only

aggregated types of resource use and, after that, deductively according to the above categories. Conversions were made assuming 8-hour workdays and 46 full weeks of work each year.

Costs for used resources were calculated based on the time used for each activity reported in the logbooks. The corresponding costs to the health system in 2019 values were calculated by multiplying the time spent by wage and the related mandatory and negotiated social insurance contribution (37.14% (36)). The mean wage for a nurse employed by a Swedish Region was SEK 34,100 (37), which was used as an approximation for the wage of staff in the health care units as we seldom knew the distribution of staff categories in logbook recordings (i.e., SEK 2,440 per day working, after adjusting for holidays and social insurance contributions, calculated as SEK 34,100*1.3714*12 months divided by 46 weeks working and 5 days per week). The approximate wage for staff employed in the DD was SEK 40-45,000 reported by the development leaders, which in the analysis was approximated to SEK 43,000 (resulting in SEK 3,077 per day working). Costs for the DD were also calculated using information from the regional budget documentation for this function, including a set budget for each year during the study. The set budget, for instance, was to cover 80% of one full-time employee. The budget was further contextualised using the total population of Region Dalarna (approximately 281,000 inhabitants) and the total health care spending during the study period. Year-end reports from the region reported costs of SEK 8,985-11,106 million (38) during the years 2016-2018. Costs were analysed from the health systems perspective, including all costs to the care organization, and expressed in 2019 value. No discounting was deemed necessary because costs were only reported descriptively.

Statistical analyses

Time reported in the logbooks was reported descriptively by types of activity associated with implementing a more PCC approach (Supplementary file S2). The unit-specific outcomes were reported separately for each unit, using graphs to illustrate trends over time. The linear equation was used to indicate trends in the length of stay while survival analysis was used to examine time to

For peer review only - http://bmjopen.bmj.com/site/about/guidelines.xhtml

readmission. Where applicable, analyses were adjusted for overcrowding during the initial hospitalization. All analyses were conducted using Stata Statistical Software: Release 17.0. College Station, TX: StataCorp LLC.

Patient and Public Involvement

This project did not include patient or public involvement in developing the research questions, design, conduct, choice of outcome measures, or recruitment.

Results

The time reported in logbooks was between 3 and 13 working days per staff member in the participating units (Table 2), although the time spent was not equally distributed among the teams in each unit. In total, time reported in logbooks from the DD corresponded to 267 full days of work (account for work done by the development unit overall, including the time used by two assigned development leaders) and 95-275 full days of work per unit for the health care units. This corresponded to approximately 3-13 working days per staff member in each health care unit over the three-year study period (mean 5.5 working days; median 4 working days). The cumulative distribution of time reported in logbooks indicate that some units reported an equal workload associated with the implementation between years while others reported a more varied pattern of workload (Fig 1).

Table 2: Resource use reported in logbooks by categories of costs and organisational units.

Organisational unit (approx. employees)	Total	Cost categories[a]		
	working days[b] (per employee)	Implementation strategies working days[b] (% of Total)	Service delivery working days[b] (% of Total)	Research/development working days[b] (% of Total)
Geriatric unit 1 (50)	185 (4)	19 (10)	161 (87)	5 (3)
Geriatric unit 2 (50)	167 (3)	95 (57)	67 (40)	5 (3)
Nephrology unit (40)	162 (4)	68 (42)	74 (46)	20 (12)
Primary care unit (70)	275 (4)	44 (16)	212 (77)	19 (7)
Psychiatric unit 1 (20)	263 (13)	15 (6)	236 (90)	12 (5)
Psychiatric unit 2 (20)	95 (5)	19 (20)	74 (78)	2 (2)
DD (18)	267 (-)[c]	234 (88)	5 (2)	28 (10)

Numbers are rounded.

For peer review only - http://bmjopen.bmj.com/site/about/guidelines.xhtml

For peer review only

[a] Developed from previously reported cost categories in implementation programmes: 1) costs for executing implementation strategies, 2) excess costs for service delivery as implementation/service changes, 3) opportunity costs to providers and patients, and 4) research/development costs (35). No account was taken for changes in costs for direct patient care (category 3).
[b] Assuming 8-hour working days.
[c] Time per employee was not calculated for the Department of Development as the time used was mainly spent by the two persons engaged in providing operational support.
Abbreviations: DD = Department for development.

Fig 1: Accumulation of working days for the Department for Development and the care units by categories of costs in implementation programmes.

Figure legend: * Resource use analysed by year (other graphs are based on actual dates).

Activities categorised as being related to the implementation strategies (88% of time reported by te DD, 6-57% for the other units) included planning and preparatory work for the learning seminars, as well as conducting/participating in those seminars. For the DD, service delivery (2% of their total reported time) was interpreted to include participation in regional decision making, administrative work, reporting to the region and collaboration with other organisations (e.g., unions). For the units, it included IT solutions, reporting, educational activities, development of teams, and care development (corresponding to 40-90% of their reported time). Research and development costs comprised interactions with the research team and external collaboration with other regions (10% of the time reported by the DD, 2-12% for the other units). A more detailed list of the activities reported by the units and the distribution of reported time between activities are reported in eTable 1 (Supplementary file S3). Training of staff was a major part of this implementation programme. For some employees, the training included up to three learning seminars (7-8 hours/seminar, difference based on transportation), i.e., implementation strategies. In most units additional training sessions were added (for a selected group or all employees) to facilitate the implementation of a more PCC approach, such as training in communication techniques, i.e., service delivery categorized under either care development or team development depending on the type of training chosen (eTable 1).

For peer review only - http://bmjopen.bmj.com/site/about/guidelines.xhtml

In Table 3, only salary costs for the healthcare units are listed, estimated based on an approximate wage per day of a nurse or DD staff, respectively. For the healthcare units, salary costs ranged from SEK 231,582 to SEK 669,922. For DD, the salary costs for activities reported in the logbooks were calculated to SEK 822,633 However, the DD included both personnel (budgeted as 80% of one full-time employment over the whole period, corresponding to 552 working days[1]) and resources for training (seminars and workshops), development work and IT resources, as well as internal and external communication. For 2016, 2017, 2018, and 2019, the annual budget was SEK 500,000, SEK 600,000, SEK 600,000, and SEK 880,000, respectively. Thus, for the work organised by the DD during the period 2016-2019 the total budget was SEK 2,580,000, of which approximately SEK 1,698,342 were salary costs for the 80% employment to support the units, making it clear that the data collection through logbooks, SEK 822,633, did not capture all activities conducted by the DD (i.e., the 80% of a full-time employment over the whole study period; SEK 1,698,342).

Table 3: Costs for time reported in logbooks by categories of costs and organisational units. Costs include wages and social insurance contributions but excludes overhead costs (e.g., facilities).

Organisational unit (approx. employees)	Total	Cost categories[a]		
		Implementation strategies	Service delivery	Research/development
	SEK (% of Total cost for all units combined)	SEK	SEK	SEK
Geriatric unit 1 (50)	451,673 (12)	46,358	393,535	11,779
Geriatric unit 2 (50)	406,930 (11)	231,638	163,778	11,514
Nephrology unit (40)	394,811 (11)	165,761	179,638	49,413
Primary care unit (70)	669,922 (19)	107,661	516,039	46,223
Psychiatric unit 1 (20)	642,001 (18)	36,598	575,740	29,662
Psychiatric unit 2 (20)	231,582 (6)	46,358	181,493	3,731
DD (18)	822,633 (23)	720,334	15,384	86,916
Total cost	3,619,552 (100)	1,354,708	2,025,607	239,238

Numbers are rounded.
[a] Developed from previously reported cost categories in implementation programmes: 1) costs for executing implementation strategies, 2) excess costs for service delivery as implementation/service changes, 3) opportunity costs to providers and patients, and 4) research/development costs (35). No account was taken for changes in costs for direct patient care (category 3).
Abbreviations: DD = Department for development.

[1] Calculated as 80% of full-time over 3 years (with full time being 46 weeks per year).

For peer review only

For peer review only - http://bmjopen.bmj.com/site/about/guidelines.xhtml

Based on the target population of Region Dalarna, the total cost of the DD (salaries and other expenses) corresponds to SEK 2.30 per citizen per year. Thus, the DD budget corresponded to 0.009 % of the total health care budget over the studied period (June 2016-May 2019). The approximate exchange rate is SEK 10 ≈ EUR 1 (exchange rate in 2019 was mean SEK 10.5892 [range SEK 10.1874-10.9056] per EUR 1 (39)), which means the total budget for the DD was approximately EUR 258,000 during 2016-2019.

Salary costs associated with time reported in logbooks indicated 23% of costs (SEK 822,633 of a total SEK 3,619,552, Table 3) for this implementation occurred in the DD. Including the total budgeted funding for the DD (SEK 2,580,000), their proportion increased to 48% of the total cost.

Unit specific measures

The first geriatric unit (eFigure 1) and the psychiatric units (combined in eFigure 2) reported a decrease in the average length of stay among their patient populations during the study period. Further examination of data from the psychiatric units showed that this trend was not explained by overcrowding (results not shown). Time to readmission within the first 10 months after discharge was similar between years in psychiatric units (combined in eFigure 3). The apparent increase in length of stay in the psychiatric units (eFigures 2 and 3) is affected by data availability and how the graph was created, with longer hospitalizations started before the end date of the data collection continuing into the second half of 2019 while no shorter stays are added.

The second geriatric unit reported a similar number of discharges across years (range 675-710) but refrained from providing further unit-specific data due to significant changes in the organisation (textbox 1). The nephrology and primary care units also refrained from delivering the planned outcome data due to other major changes in their work processes conducted during the same period as the implementation of a more person-centred care approach. Consequently, these data

For peer review only - http://bmjopen.bmj.com/site/about/guidelines.xhtml

are only slightly related to the change under study. The primary care unit had approximately 14,000 listed patients throughout the study period.

Textbox 1: Staff turnover and changing methods for prioritising patients: an example from Geriatric care and rehabilitation (Geriatric unit 2).

Initial contacts to discuss the evaluation of resource use were able to identify several aspects that could be relevant to follow during the development towards a more PCC approach, including work hours, number of patients, work environment follow-up, and information that should be accessible through the administrative registers. These aspects were considered especially important due to the shortage of registered nurses.

When the project was nearing its end, new contacts were made. None (or very few) of the people involved in the project's launch remained in the organisation in 2019 due to changing roles or retirement. Concerns were expressed that they had not had time to actively work on the person-centeredness due to staff shortages and related downsizing of patient beds. The reduction in patient beds was described as having a budget for 18 patients but only staff enough to admit 10. They had handled the lack of nurses by changing from a registered nurse and an assistant nurse working in pairs to each nurse working with two assistants and transferring tasks to the physicians. Daily discussions were held to ensure that those in most need of the services provided by the geriatrics unit were cared for at the unit, and not moved to other sections of the hospital due to overcrowding.

Thus, it was concluded that it would not be relevant to evaluate any of the initial planned unit-specific outcomes given that the implementation process of a more PCC approach had been given a secondary role compared to other changes in the unit. In Table 2 it can be seen that this unit used among the lowest number of working days per person of all included units and had the highest

For peer review only - http://bmjopen.bmj.com/site/about/guidelines.xhtml

percentage (57%) of that work distributed to the initial planning and participation in learning seminars (cost category implementation strategies). Combined, the perceived secondary role of the implementation process and the discrepant time distribution compared to other included units can indicate the implementation process was not complete.

Discussion

The health care units logged on average 5.5 working days per staff member for implementing a more PCC approach, but the number of days varied largely between units (range 3-13 working days). In the healthcare units, 6-57% of the time reported in logbooks was assessed as being used for implementation strategies, 40-90% for service delivery, and 2-12% for research/development. As expected, the distribution of time used by the DD staff differed considerably from that of the other participating units, with most of the logged time (88%) assigned to implementation strategies. While the time spent and salary costs associated with the implementation process were considerable, usually corresponding to at least 0.5-1 year of full-time employment per unit, the total cost was small compared to the entire health care budget. Although budgeting for this implementation was only available for the DD, at least half of the costs occurred in the other healthcare units. Unit-specific outcomes from three of the units showed no clear effect of the implementation, and in general, the healthcare units reported that other factors had affected their throughput more during this study period than the implementation of a more PCC.

To our knowledge, this is the first study investigating the different components contributing to the time and costs spent on implementing a more PCC approach. Several studies have showcased how implementation costs should be measured (35,40–42), but there is a shortage of studies measuring the costs from an implementation standpoint (43,44). The strength of the study is that it was conducted by an independent research body that included researchers from different disciplines relevant to the interpretation of the results. Another strength was that the included units were given multiple opportunities to clarify and correct any oversights in their records (such as not writing the

For peer review only - http://bmjopen.bmj.com/site/about/guidelines.xhtml

number of participants during a specific action) and potential misunderstandings regarding interpretation. Although the independence of the researchers was a strength, it also contributed to the main limitation of the study, the data collection process. The staff had no time set off for the logbooks and were not provided with extra time or staff to conduct the implementation process. Some reporters filled in large parts of the logbooks retroactively (not only 2016 data), potentially resulting in recall bias, and in some cases, one of our research group members (MT or HF) took part in this work to record previous activities. It should be noted that for one of the units with the highest working day estimate in relation to their staff, one of the researchers supported the staff member in filling out the logbooks which could indicate the staff member was reminded of more tasks having been conducted than would otherwise have been the case. However, the higher estimate could also be the result of this being one of two units that were merged during part of the study, thus making the division of time spent for each of these units difficult to distinguish.

While all unit-specific outcomes had been identified by each healthcare unit as important aspects to follow during this change process, most units chose in the end to not providing the data due to being more affected by other changes in the workplaces. For the units providing these data there is still an assumption that most of the observed changes were due to other factors than the implementation of a more PCC. The reason for initiating the collection of unit-specific outcomes was to make the evaluation more relevant to the participating healthcare units and similar units elsewhere, and to ensure that the implementation of a more PCC approach was at least not associated with any large negative impact on patient throughput. However, due to other parallel changes in these units it did not provide any conclusive results. The commitment shown by staff in the participating units is exemplified by them participating in the change process and providing materials and responses to questions during analysis, regardless of the lack of time provided for their participation. This commitment can also be discussed regarding the planned observational approach of the study. Here, it can be argued that by engaging in research and data collection, the staff involved in the implementation may have been affected (i.e., through social desirability bias or being reminded of

For peer review only

For peer review only - http://bmjopen.bmj.com/site/about/guidelines.xhtml

the implementation process by the researchers) and thus to a larger extent interacting with it. While initially intended, we did not have logbooks from leaders (Chief executives) of this implementation process at the main organisational level in the region, but that should have implied a small cost compared to that of changing care practice. It should also be acknowledged that the division of costs by categories is not self-evident. Costs for changes in service delivery could also to some extent be seen as costs for implementation strategies. For example, the initial learning seminars were assumed to be part of the implementation strategies. However, if the health care unit later decided that in their work to change practice, the staff needed special training (e.g., in interprofessional rounds or motivational interviewing), we interpreted this as part of changing how service was delivered. An alternative interpretation would have been to see this training as part of an iterative process of implementation strategies conducted at different levels of the organisation, i.e., the study distinguishes between centrally planned implementation strategies and strategies conducted by the health care delivery organisation (45). When considering the limitations of the study, our estimated working days and costs should be interpreted with caution. These findings are only part of a picture that needs to be further developed in future studies and frameworks to assess the economic impact of implementation.

Using the cost components suggested by Wagner et al. (40) as a basis, additional costs would include overhead costs (e.g., facilities). Moreover, the inclusion of research and development costs should be broken down into what would be sunk costs (i.e., one-time investments) versus costs for development and scaling up (such as communications within the region to support others) versus costs that are solely for research purposes (i.e., the research interviews). However, it has been argued that the costs of research should be reported (46), not to inform clinical practice but to assess the costs associated with evaluation. Here, we are only reporting on the costs for the region to participate in the research study. We hope our approach and findings might help others design similar studies to follow implementation processes more in depth.

For peer review only - http://bmjopen.bmj.com/site/about/guidelines.xhtml

Changes in health care can aim to either improve effectiveness, i.e., save money while producing at least as much health as before, or increase care quality, or a combination of the two (47). Today, there is a growing body of evidence of improved patient outcomes and possibilities for cost savings by shifting to PCC from randomised controlled trials and quasi-experimental studies in Sweden and internationally (10,28,29,48,49). In comparison, little is known about the “hidden” costs of preparatory work, training, and monitoring outcomes during implementation (44), costs that are investigated in this study. In a recent systematic review of the literature, only six studies were identified that specified such costs. It has been argued that any implementation effort should be preceded by ex-ante modelling to compare the expected returns of implementing the intended change to the predicted costs of implementing the change (35,43,50). However, this is not always the case, or the results are at least not available to the research community.

One crucial aspect of the studied change process was that it was made clear that health care units would not be provided with extra time or other resources for the implementation. This was reported by both the central regional organisation and the management in each health care unit, with staff shortage being the main reason. However, we fund that units implementing more PCC used a considerable amount of time for this implementation process. Thus, the time reported in logbooks could be interpreted to refer to the time that otherwise would have been used for other work in the health care units. Had the implementation of more PCC not been made, the reported time would still have been used in the units. A hypothetical comparator, an alternative intervention, could have used the same amount of time to implement some other change in practice, such as developing and implementing clinical guidelines or care paths (which thus would suggest the opportunity cost of this implementation process). Because several units had assigned quality developers or specialist nurse students with tasks associated with the implementation process, which equalled several working days spent per staff member, the used time likely offset other tasks otherwise conducted by the staff. In addition, staff in some settings expressed that it was time-consuming to provide PCC in the immediate time frame but it could potentially be beneficial later in the care process (20). Together

For peer review only

For peer review only - http://bmjopen.bmj.com/site/about/guidelines.xhtml

with recent reporting that increased person-centredness was associated with higher job strain (18), it is likely that additional resources during the initial period had resulted in improved uptake.

It should also be noted that the possibility to influence resource use is probably influenced by the patient groups in each health care unit and to what extent work with these patients is already streamlined. Considering a patient group for which clinical guidelines determines the frequency of follow-up, there is less opportunity to change the number of visits and thus costs will be similar even if the care changes. If there is instead a patient group experiencing unmet needs and much acute unplanned care, it can be assumed that changing how patients experience their health care can change how many visits are needed.

While several of the health care units expressed that they had not completed the implementation within the study period, assessing its success need to be based on still ongoing studies of patients' experiences. However, the findings clearly demonstrate that there is a non-zero cost of implementing a more PCC approach; costs that should be acknowledged in future research and implementation processes. The study also points towards potential improvements in how to study implementation costs, through e.g., recurrent questionnaires instead of logbooks that are collected at the end of the study period. Furthermore, due to the reported high staff turnover, the costs for changes in service delivery may to some extent continue in training of new staff. Considering our findings in light of recent updates on the use of economic evaluation in implementation science to guide decision-makers (51,52), future studies should thus distinguish all costs associated with implementation science, including implementation costs, intervention costs, and downstream costs (17) of a more PCC approach, as well as for other healthcare programmes.

Conclusions

The study found that a large part of resources used for this implementation of more PCC occurred in the DD, although at least half of the costs occurred in the healthcare units. Our findings suggest that the main costs associated with implementing a more PCC approach in ordinary practice resulted

For peer review only - http://bmjopen.bmj.com/site/about/guidelines.xhtml

from implementing strategies and service delivery. In contrast, research and development costs were small by comparison. Moreover, the cost of providing a central support function corresponds to a tiny proportion of the total health budget. While there are limitations in how the study was conducted, it clearly demonstrates a non-zero cost of implementing a more PCC approach, thus implicating that future research should capture costs. Not accounting for the added strain on healthcare units can result in delay or inability to implement the new care model.

Supporting information captions

Supplementary file S1: Study protocol

Supplementary file S2: Analysis dataset

Supplementary file S3: Supplementary tables and figures

Funding statement

The study was financed by the University of Gothenburg Centre for Person-Centred Care (GPCC) and Dalarna University. The funding body had no role in the design of the study, in collection, analysis, and interpretation of data, or in writing the manuscript.

Competing interest's statement

The authors declare that they have no competing interests.

Author's contribution

HG, MT, HF, and LW contributed to the design of the work; HG, MT and HF contributed to the acquisition, analysis, and interpretation of the data; HG drafted the manuscript while MT, HF, and LW substantively contributed to the final manuscript; HG, MT, HF, and LW all have approved the submitted version and agree both to be personally accountable for the author's contributions and to ensure that questions related to the accuracy or integrity of any part of the work, even ones in which

For peer review only

For peer review only - http://bmjopen.bmj.com/site/about/guidelines.xhtml

the author was not personally involved, are appropriately investigated, resolved, and the resolution documented in the literature.

Acknowledgments

We thank all contributing health care units. Thanks also to Dr Leslie Shaps for language editing.

Data sharing statement

All relevant data are within the manuscript and its Supporting Information files (Supplementary file S2).

Ethics approval statement

The study was approved by the regional ethics committee in Uppsala (approval number: 2017/195). Logbooks were filled in by managers/project leaders in each health care unit who had consented to this registration (verbally consented at the start of the study, and later through providing the information for analysis). Unit specific data were obtained from administrative systems without information on individual patients or staff, by the same consenting managers/project leaders. There was thus no data collection (nor informed consent) from patients or individual staff. Results for each unit were communicated to provide an opportunity for the units to question and correct misunderstandings. All methods were performed in accordance with relevant guidelines and regulations, and in accordance with the ethical approval for this study.

For peer review only

References

1. Nolte E, Merkur S, Anell A, European Observatory on Health Systems and Policies, redaktörer. Achieving person-centred health systems: evidence, strategies and challenges. Cambridge; New York, NY: Cambridge University Press; 2020. (European Observatory on Health Systems and Policies).

2. Rosengren K, Brannefors P, Carlstrom E. Adoption of the concept of person-centred care into discourse in Europe: a systematic literature review. J Health Organ Manag. 13 september 2021;35(9):265–80.

3. Sverige Utredningen Samordnad utveckling för god och nära vård. God och nära vård: en primärvårdsreform [Good quality, local health care – A primary care reform] [Internet].

For peer review only - http://bmjopen.bmj.com/site/about/guidelines.xhtml

Stockholm; Stockholm: Norstedts Juridik; Elanders Sverige AB; 2018. (Statens offentliga utredningar : SOU). Report No.: 9789138248065. Tillgänglig vid: https://www.regeringen.se/rattsliga-dokument/statens-offentliga-utredningar/2018/06/sou-201839/

4. Mead N, Bower P. Patient-centredness: a conceptual framework and review of the empirical literature. Soc Sci Med 1982. 2000;51(7):1087–110.

5. Ekman I, Swedberg K, Taft C, Lindseth A, Norberg A, Brink E, m.fl. Person-centered care--ready for prime time. Eur J Cardiovasc Nurs. 2011;10(4):248–51.

6. Harding E, Wait S, Scrutton J. The state of play in person-centred care: A pragmatic review of how person-centred care is defined, applied and measured [Internet]. The Health Policy Partnership Ltd; 2015 s. 1–140. Tillgänglig vid: http://www.healthpolicypartnership.com/person-centred-care/

7. Håkansson Eklund J, Holmström IK, Kumlin T, Kaminsky E, Skoglund K, Höglander J, m.fl. ”Same same or different?” A review of reviews of person-centered and patient-centered care. Patient Educ Couns. 2019;102(1):3–11.

8. World Health Organization. Framework on integrated people-centred health services. WHO; 2016 s. 1–12.

9. Sharma T, Bamford M, Dodman D. Person-centred care: an overview of reviews. Contemp Nurse. 2015;51(2–3):107–20.

10. Hansson E, Ekman I, Swedberg K, Wolf A, Dudas K, Ehlers L, m.fl. Person-centred care for patients with chronic heart failure - a cost-utility analysis. Eur J Cardiovasc Nurs J Work Group Cardiovasc Nurs Eur Soc Cardiol. 2015;15(4):276–84.

11. Sahlen KG, Boman K, Brännstrom M. A cost-effectiveness study of person-centered integrated heart failure and palliative home care: Based on a randomized controlled trial. Palliat Med. 2016;30(3):296–302.

12. Swedberg K, Cawley D, Ekman I, Rogers HL, Antonic D, Behmane D, m.fl. Testing cost containment of future healthcare with maintained or improved quality-The COSTCARES project. Health Sci Rep. juni 2021;4(2):e309.

13. Nkhoma KB, Cook A, Giusti A, Farrant L, Petrus R, Petersen I, m.fl. A systematic review of impact of person-centred interventions for serious physical illness in terms of outcomes and costs. BMJ Open. 13 juli 2022;12(7):e054386.

14. Chenoweth L, King MT, Jeon YH, Brodaty H, Stein-Parbury J, Norman R, m.fl. Caring for Aged Dementia Care Resident Study (CADRES) of person-centred care, dementia-care mapping, and usual care in dementia: a cluster-randomised trial. Lancet Neurol. april 2009;8(4):317–25.

15. Chenoweth L, Forbes I, Fleming R, King MT, Stein-Parbury J, Luscombe G, m.fl. PerCEN: a cluster randomized controlled trial of person-centered residential care and environment for people with dementia. Int Psychogeriatr. juli 2014;26(7):1147–60.

16. Jones RA, Dougherty M, Martin S. Program evaluation of a unit reengineered for patient-focused care. Holist Nurs Pract. april 1997;11(3):31–46.

For peer review only

For peer review only - http://bmjopen.bmj.com/site/about/guidelines.xhtml

17. Gold HT, McDermott C, Hoomans T, Wagner TH. Cost data in implementation science: categories and approaches to costing. Implement Sci IS. 28 januari 2022;17(1):11.

18. Van Diepen C, Fors A, Ekman I, Bertilsson M, Hensing G. Associations between person-centred care and job strain, stress of conscience, and intent to leave among hospital personnel. J Clin Nurs. mars 2022;31(5–6):612–22.

19. Fridberg H, Wallin L, Wallengren C, Kottorp A, Forsman H, Tistad M. Development and evaluation of the measurement properties of a generic questionnaire measuring patient perceptions of person-centred care. BMC Health Serv Res. 20 oktober 2020;20(1):960.

20. Fridberg H, Wallin L, Tistad M. [The innovation characteristics of person-centred care as perceived by healthcare professionals: an interview study employing a deductive-inductive content analysis guided by the consolidated framework for implementation research. BMC Health Serv Res. 03 september 2021;21(1):904.

21. Fridberg H, Wallin L, Tistad M. Operationalisation of person-centred care in a real-world setting: a case study with six embedded units. BMC Health Serv Res. 14 september 2022;22(1):1160.

22. Fridberg H, Wallin L, Tistad M. Tracking, naming, specifying, and comparing implementation strategies for person-centred care in a real-world setting: a case study with seven embedded units. BMC Health Serv Res. 24 november 2022;22(1):1409.

23. Tistad M, Wallin L, Carlström E. A comparison of three organisational levels in one health care region in Sweden implementing person-centred care: Coupled, decoupled or recoupled in a complex organization. BMC Health Serv Res. 14 februari 2022;22(1):196.

24. Region Dalarna [Internet]. [citerad 29 juni 2022]. Other languages - Region Dalarna. Tillgänglig vid: https://www.regiondalarna.se/ovrigt/om-webbplatsen/other-languages/

25. Glenngård AH. The Swedish Health Care System. I: International Profiles of Health Care Systems 2020 [Internet]. The Commonwealth Fund; 2020. s. 1–228. Tillgänglig vid: https://www.commonwealthfund.org/international-health-policy-center/countries

26. Britten N, Ekman I, Naldemirci Ö, Javinger M, Hedman H, Wolf A. Learning from Gothenburg model of person centred healthcare. BMJ. 01 2020;370:m2738.

27. Olsson LE, Karlsson J, Ekman I. The integrated care pathway reduced the number of hospital days by half: a prospective comparative study of patients with acute hip fracture. J Orthop Surg. 2006;1(Journal Article):3-799X-1–3.

28. Gyllensten H, Koinberg I, Carlstrom E, Olsson LE, Hansson Olofsson E. Economic evaluation of a person-centred care intervention in head and neck oncology: results from a randomized controlled trial. Support Care Cancer Off J Multinatl Assoc Support Care Cancer. 2019;27(5):1825–34.

29. Pirhonen L, Bolin K, Olofsson EH, Fors A, Ekman I, Swedberg K, m.fl. Person-Centred Care in Patients with Acute Coronary Syndrome: Cost-Effectiveness Analysis Alongside a Randomised Controlled Trial. PharmacoEconomics - Open. 2019;3(4):495–504.

30. Skivington K, Matthews L, Simpson SA, Craig P, Baird J, Blazeby JM, m.fl. A new framework for developing and evaluating complex interventions: update of Medical Research Council guidance. BMJ. 30 september 2021;374:n2061.

For peer review only - http://bmjopen.bmj.com/site/about/guidelines.xhtml

31. Craig P, Dieppe P, Macintyre M, Michie S, Nazareth I, Petticrew M. Developing and evaluating complex interventions: new guidance [Internet]. Medical Research Council; 2006 s. 1–39. Tillgänglig vid: https://www.mrc.ac.uk/documents/pdf/complex-interventions-guidance/

32. Moore GF, Audrey S, Barker M, Bond L, Bonell C, Hardeman W, m.fl. Process evaluation of complex interventions: Medical Research Council guidance. BMJ. 2015;350:h1258.

33. Proctor EK, Landsverk J, Aarons G, Chambers D, Glisson C, Mittman B. Implementation research in mental health services: an emerging science with conceptual, methodological, and training challenges. Adm Policy Ment Health. 2009;36(1):24–34.

34. Dawson A, Sim J. The nature and ethics of natural experiments. J Med Ethics. oktober 2015;41(10):848–53.

35. Hoomans T, Severens JL. Economic evaluation of implementation strategies in health care. Implement Sci. 18 december 2014;9:168.

36. Sveriges Kommuner och Regioner [Swedish Association of Local Authorities and Regions] [Internet]. [citerad 29 april 2020]. Arbetsgivaravgifter [General payroll tax]. Tillgänglig vid: https://skr.se/ekonomijuridikstatistik/ekonomi/budgetochplanering/arbetsgivaravgifter.1290.html

37. Sverige i siffror [Internet]. [citerad 29 april 2020]. Lönesök – Hur mycket tjänar...? [Wage search - How much do ... earn?]. Tillgänglig vid: http://www.scb.se/hitta-statistik/sverige-i-siffror/lonesok/

38. Årsredovisning - Region Dalarna [Year-end reports - Region Dalarna] [Internet]. [citerad 02 september 2021]. Tillgänglig vid: https://www.regiondalarna.se/om-regionen/arsredovisning/

39. Årsgenomsnitt valutakurser (ackumulerat) 2019 [Annual average exchange rates (accumulated) 2019] [Internet]. [citerad 30 juni 2022]. Tillgänglig vid: https://www.riksbank.se/sv/statistik/sok-rantor--valutakurser/arsgenomsnitt-valutakurser/

40. Wagner TH, Yoon J, Jacobs JC, So A, Kilbourne AM, Yu W, m.fl. Estimating Costs of an Implementation Intervention. Med Decis Mak Int J Soc Med Decis Mak. november 2020;40(8):959–67.

41. Krebs E, Nosyk B. Cost-Effectiveness Analysis in Implementation Science: a Research Agenda and Call for Wider Application. Curr HIV/AIDS Rep. juni 2021;18(3):176–85.

42. Powell BJ, Fernandez ME, Williams NJ, Aarons GA, Beidas RS, Lewis CC, m.fl. Enhancing the Impact of Implementation Strategies in Healthcare: A Research Agenda. Front Public Health. 2019;7:3.

43. Reeves P, Edmunds K, Searles A, Wiggers J. Economic evaluations of public health implementation-interventions: a systematic review and guideline for practice. Public Health. april 2019;169:101–13.

44. Roberts SLE, Healey A, Sevdalis N. Use of health economic evaluation in the implementation and improvement science fields—a systematic literature review. Implement Sci. 15 juli 2019;14(1):72.

For peer review only

For peer review only - http://bmjopen.bmj.com/site/about/guidelines.xhtml

45. Leeman J, Birken SA, Powell BJ, Rohweder C, Shea CM. Beyond ”implementation strategies”: classifying the full range of strategies used in implementation science and practice. Implement Sci IS. 03 november 2017;12(1):125.

46. Visser BJ, Buijink AWG, Grobusch MP. Reporting of medical research costs. Improving transparency and reproducibility of medical research. Methods Inf Med. 2014;53(4):329–31.

47. Chisholm D, Evans DB. Economic evaluation in health: saving money or improving care? J Med Econ. 01 januari 2007;10(3):325–37.

48. Pirhonen L, Gyllensten H, Fors A, Bolin K. Modelling the cost-effectiveness of person-centred care for patients with acute coronary syndrome. Eur J Health Econ HEPAC Health Econ Prev Care. 07 september 2020;21(9):1317–27.

49. Pirhonen L, Gyllensten H, Olofsson EH, Fors A, Ali L, Ekman I, m.fl. The cost-effectiveness of person-centred care provided to patients with chronic heart failure and/or chronic obstructive pulmonary disease. Health Policy OPEN. 01 december 2020;1:100005.

50. Sutton M, Garfield-Birkbeck S, Martin G, Meacock R, Morris S, Sculpher M, m.fl. Economic analysis of service and delivery interventions in health care [Internet]. Southampton (UK): NIHR Journals Library; 2018 [citerad 20 mars 2020]. (Health Services and Delivery Research). Tillgänglig vid: http://www.ncbi.nlm.nih.gov/books/NBK481909/

51. Saldana L, Ritzwoller DP, Campbell M, Block EP. Using economic evaluations in implementation science to increase transparency in costs and outcomes for organizational decision-makers. Implement Sci Commun. 11 april 2022;3(1):40.

52. O’Leary MC, Hassmiller Lich K, Frerichs L, Leeman J, Reuland DS, Wheeler SB. Extending analytic methods for economic evaluation in implementation science. Implement Sci IS. 15 april 2022;17(1):27.

For peer review only

For peer review only - http://bmjopen.bmj.com/site/about/guidelines.xhtml

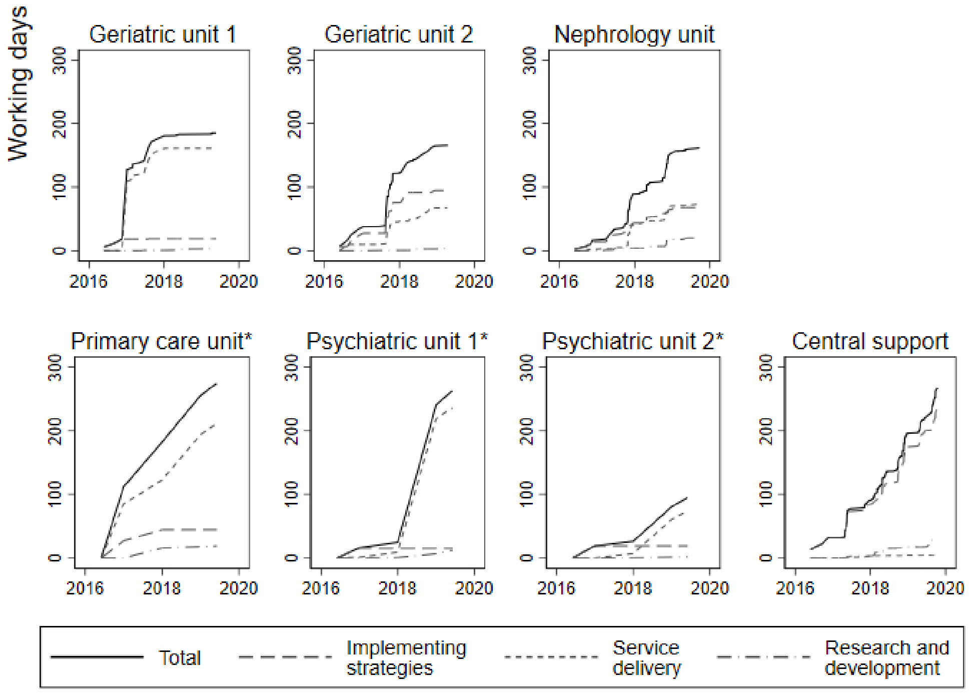

Accumulation of working days for the Department for Development and the care units by categories of costs in implementation programmes.
Figure legend: * Resource use analysed by year (other graphs are based on actual dates).

246x178mm (72 x 72 DPI)

Health economics of implementing person-centred care: the IMPROVE project

Suggested authors (order to be decided)

Hanna Gyllensten, Malin Tistad, Helena Fridberg, Eric Carlström, Lars Wallin

Background

Healthcare systems in many countries experiences increasing economic demands, both through the development of new technologies and treatments, and through a changing age distribution in the population resulting in more people with multiple chronic conditions.

Person-centred care (PCC) has been promoted both as a means to handle dissatisfaction with healthcare access and delivery,(1) and as potentially cost-saving or cost containing through more effective use of resources.(2) PCC acknowledges and endorses every person´s resources, interests, and needs, comprising shared responsibility and power, as well as a coordinated care and treatment.(3–5) It is related to the so called integrated people-centred health services promoted by the World Health Organization(6) albeit without the community perspective included in that framework.

However, PCC it is often described as a complex intervention; an intervention containing several interacting components,(7,8) through including elements tailored to each participant, activities achieved in different ways, and with multiple and sometimes varying outcomes and goals for different participants. Thus, research into its use and effects needs to handle the complexity involved,(7) including a thorough understanding of how the intervention is produced through e.g., process evaluation of the intervention created.(9) In a conceptual model for such implementation research, Procter and colleagues elaborated on the outcomes evaluation, including intervention outcomes, service outcomes and client outcomes.(10)

This study is, as far as we know one of few measuring the cost of implementing person-centred care. Several studies suggests cost-saving as one reason to implement person-centred care,(2,11) but the cost to implement the method is seldom mentioned. Even though, person-centred care has an extensive impact on the heath-care industry. In Sweden legislators and health care providers has introduced person-centred care on the agenda of development and management-control efforts is more and more about implementing person-centred care. In this study we measure the cost of such an implementation.

Aims

The aims of this project was to map the resources used in implementing PCC, in the healthcare unit as well as on a regional level. A secondary aim was to explore how this implementation affected the budget, from the healthcare provider's perspective.

> *Ursprunglig formulering:*
>
> *• Att kartlägga resursförbrukning för att införa PCV på lokal nivå respektive på regional/landstingsnivå.*
>
> *• Att undersöka hur landstingets budget påverkas av att införa PCV.*

For peer review only - http://bmjopen.bmj.com/site/about/guidelines.xhtml

Methods

Contextual factors

Region Dalarna decided in 2016 to promote a more person-centred care approach throughout the public healthcare system, as part of their work towards efficient healthcare practices (in parallel with other ongoing projects within the so called “Struktur- och förändringsarbetet”, including projects related to priority setting and resource allocation in local health systems). As part of this implementation process, changes were made on micro, meso, and macro level in the region. The *Implementing person centred care: Process evaluation of strategies, leadership and health economy* (IMPROVE) project was established to follow this implementation process. In the project, six healthcare units were followed to explore the strategies used to implement a more person-centred care and the associated outcomes, as well as the policies and leadership related aspects of this process. Data was collected through logbooks, questionnaires, interviews and review of medical records, to give a comprehensive overview of how the process evolved over time.

The implementation process studied in the IMPROVE project was introduced and implemented by Region Dalarna. The process evaluation was conducted in parallel by researchers from collaborating universities partnering with staff in the healthcare units.

Data collection

Six healthcare units were included in the process evaluation, selected based on being early adapters to the implementation of PCC. Included healthcare units were followed from six months before to 18 months after the introduction of PCC. The units included were specialized in nephrology, geriatric rehabilitation, psychiatry and primary care, respectively.

Staff involved in introducing PCC were asked to complete logbooks of their activities and hours spent. Information from the logbooks was thus used to identify both which activities were perceived by personnel in different segments of the healthcare organization to be related to implementing PCC, and to estimate the time used for this implementation. Logbooks were also collected from operational support personnel involved in supporting care units during the implementation. Costs for used resources was calculated based on the time used for each type of activity (categorized inductively after data collection) times the average cost per hour for the relevant staff category, retrieved from the healthcare provider.

In addition, included units were asked to provide their own suggestions for evaluating the economic impact of PCC on their practices. Relevant data was collected from each unit retrospectively, and the units were asked to comment on any potential time trends visible in the data.

A third data source was administrative data collected from the region, including economic results, patient quantities, mean length of admission, and hours worked. This information was used to provide a background to the data collected from logbooks and included healthcare units.

Analyses

Descriptive statistics of time used and associated costs, as reported in the logbooks, will be reported by types of activities associated with the implementation of PCC. A bootstrap methodology will be used to calculate 95% confidence intervals, to account for expected skewed distributions of costs.

The unit specific outcomes will be analyzed longitudinally and described separately for each unit. Unit specific measures included:

Nephrology/dialysis unit	Distribution between haemodalysis, assisted peritoneal dialysis and unassisted peritoneal dialysis

For peer review only - http://bmjopen.bmj.com/site/about/guidelines.xhtml

	Work hours (adjusted for number of patients)
Primary care	Number of patients listed<br>Number of visits per patient
Geriatric rehabilitation	Unit 1: Length of stay<br>Unit 2: Work hours, number of patients, work environment follow-up
Psychiatrics	Unit 1: Length of stay (taking overcrowding into consideration)<br>Unit 2: Length of stay and readmissions (taking overcrowding into consideration)<br>These two units were combined during a part of the study, this will also need to be considered.

Regional administrative data will be reported descriptively for each year the study was ongoing.

Suggested tables and figures

Table 1: Overview of respondents to the logbooks, including an overview of the activities reported

Table 2: Hours and associated costs for each type of activity reported in the logbooks

Table 3: Results for the unit specific measures [*may become divided into one per unit or so*]

References

1. Sharma T, Bamford M, Dodman D. Person-centred care: an overview of reviews. ContempNurse. 2015;51(2–3):107–20.

2. Hansson E, Ekman I, Swedberg K, Wolf A, Dudas K, Ehlers L, et al. Person-centred care for patients with chronic heart failure - a cost-utility analysis. EurJCardiovascNurs. 2015;(Journal Article).

3. Mead N, Bower P. Patient-centredness: a conceptual framework and review of the empirical literature. SocSciMed. 2000;51(7):1087–110.

4. Ekman I, Swedberg K, Taft C, Lindseth A, Norberg A, Brink E, et al. Person-centered care--ready for prime time. EurJCardiovascNurs. 2011;10(4):248–51.

5. Harding E, Wait S, Scrutton J. The state of play in person-centred care: A pragmatic review of how person-centred care is defined, applied and measured [Internet]. The Health Policy Partnership Ltd; 2015 p. 1–140. Available from: http://www.healthpolicypartnership.com/person-centred-care/

6. World Health Organization. Framework on integrated people-centred health services. WHO; 2016 p. 1–12.

7. Craig P, Dieppe P, Macintyre M, Michie S, Nazareth I, Petticrew M. Developing and evaluating complex interventions: new guidance [Internet]. Medical Research Council; 2006 p. 1–39. Available from: https://www.mrc.ac.uk/documents/pdf/complex-interventions-guidance/

8. Craig P, Dieppe P, Macintyre S, Michie S, Nazareth I, Petticrew M, et al. Developing and evaluating complex interventions: the new Medical Research Council guidance. BMJ. 2008;337(Journal Article):a1655.

9. Moore GF, Audrey S, Barker M, Bond L, Bonell C, Hardeman W, et al. Process evaluation of complex interventions: Medical Research Council guidance. BMJ. 2015;350(Journal Article):h1258.

For peer review only - http://bmjopen.bmj.com/site/about/guidelines.xhtml

10. Proctor EK, Landsverk J, Aarons G, Chambers D, Glisson C, Mittman B. Implementation research in mental health services: an emerging science with conceptual, methodological, and training challenges. AdmPolicy MentHealth. 2009;36(1):24–34.

11. Sahlen KG, Boman K, Brännstrom M. A cost-effectiveness study of person-centered integrated heart failure and palliative home care: Based on a randomized controlled trial. PalliatMed. 2016;30(3):296–302.

For peer review only

Variable	Value	Label
Row		Row number for sorting
UNIT	1	Geriatric unit 1
	2	Geriatric unit 2
	3	Nephrology unit
	4	Primary care unit
	5	Psychiatric unit 1
	6	Psychiatric unit 2
	7	Central support function
Activity_cat		administration
		care_development
		documentation
		educational_activities
		external_communication
		internal_communication
		it_support
		learning_seminars
		preparatory_work
		reporting
		research
		team_development
		workplace_meetings
COST_CATEGORY	1	costs for executing implementation strategies
	2	excess costs for service delivery as implementation/service changes
	4	research/development costs
Participants		Number of participants
Time_minutes		Total time used by the participants (in minutes)
Staff_cat		Manager
		HCP = health care professionals

For peer review only

Row	UNIT	Activity_cat	COST_CATEGORY	Participants
1	1	learning_seminars	1	7
2	1	learning_seminars	1	7
3	1	learning_seminars	1	7
4	1	care_development	2	1
5	1	educational_activities	2	4
6	1	team_development	2	40
7	1	educational_activities	2	40
8	1	care_development	2	50
9	1	educational_activities	2	5
10	1	team_development	2	20
11	1	educational_activities	2	6
12	1	educational_activities	2	10
13	1	preparatory_work	1	5
14	1	care_development	2	30
15	1	research	4	3
16	1	research	4	4
17	1	research	4	5
18	1	research	4	5
19	1	research	4	3
20	1	research	4	5
21	1	research	4	5
22	1	research	4	1
23	1	research	4	1
24	1	research	4	1
25	1	research	4	1
26	1	research	4	2
27	1	research	4	2
28	1	research	4	2
29	1	research	4	2
30	1	research	4	1
31	1	research	4	1
32	2	learning_seminars	1	8
33	2	care_development	2	1
34	2	learning_seminars	1	9
35	2	learning_seminars	1	9
36	2	preparatory_work	1	10
37	2	care_development	2	1
38	2	preparatory_work	1	25
39	2	care_development	2	10
40	2	care_development	2	1
41	2	preparatory_work	1	12
42	2	preparatory_work	1	7
43	2	preparatory_work	1	1
44	2	learning_seminars	1	2
45	2	educational_activities	2	2
46	2	preparatory_work	1	12
47	2	care_development	2	40
48	2	care_development	2	2
49	2	team_development	2	4

For peer review only

50	2	team_development	2	9
51	2	preparatory_work	1	6
52	2	preparatory_work	1	4
53	2	care_development	2	9
54	2	external_communication	4	2
55	2	team_development	2	5
56	2	team_development	2	10
57	2	care_development	2	5
58	2	preparatory_work	1	25
59	2	research	4	6
60	2	research	4	4
61	2	research	4	4
62	2	research	4	7
63	2	research	4	6
64	2	research	4	4
65	2	research	4	1
66	2	research	4	1
67	2	research	4	1
68	2	research	4	1
69	2	research	4	2
70	2	research	4	2
71	2	research	4	1
72	2	research	4	1
73	3	learning_seminars	1	3
74	3	learning_seminars	1	5
75	3	preparatory_work	1	6
76	3	team_development	2	8
77	3	workplace_meetings	1	25
78	3	team_development	2	4
79	3	learning_seminars	1	7
80	3	team_development	2	7
81	3	research	4	5
82	3	learning_seminars	1	8
83	3	learning_seminars	1	4
84	3	team_development	2	2
85	3	team_development	2	8
86	3	preparatory_work	1	2
87	3	preparatory_work	1	2
88	3	research	4	5
89	3	research	4	5
90	3	preparatory_work	1	1
91	3	research	4	1
92	3	preparatory_work	1	8
93	3	preparatory_work	1	6
94	3	preparatory_work	1	1
95	3	educational_activities	2	2
96	3	learning_seminars	1	4
97	3	preparatory_work	1	5
98	3	preparatory_work	1	3
99	3	preparatory_work	1	3

For peer review only

100	3	preparatory_work	1	3
101	3	educational_activities	2	2
102	3	preparatory_work	1	5
103	3	preparatory_work	1	2
104	3	educational_activities	2	40
105	3	preparatory_work	1	4
106	3	external_communication	4	6
107	3	care_development	2	40
108	3	learning_seminars	1	5
109	3	learning_seminars	1	6
110	3	educational_activities	2	1
111	3	educational_activities	2	1
112	3	documentation	2	1
113	3	care_development	2	12
114	3	preparatory_work	1	5
115	3	preparatory_work	1	2
116	3	educational_activities	2	1
117	3	care_development	2	4
118	3	care_development	2	1
119	3	care_development	2	1
120	3	preparatory_work	1	3
121	3	care_development	2	4
122	3	learning_seminars	1	9
123	3	preparatory_work	1	4
124	3	it_support	2	1
125	3	care_development	2	2
126	3	educational_activities	2	1
127	3	preparatory_work	1	5
128	3	preparatory_work	1	6
129	3	care_development	2	5
130	3	learning_seminars	1	5
131	3	care_development	2	4
132	3	preparatory_work	1	5
133	3	care_development	2	30
134	3	external_communication	4	6
135	3	external_communication	4	6
136	3	preparatory_work	1	6
137	3	educational_activities	2	4
138	3	educational_activities	2	4
139	3	care_development	2	4
140	3	learning_seminars	1	5
141	3	educational_activities	2	4
142	3	learning_seminars	1	4
143	3	care_development	2	4
144	3	care_development	2	5
145	3	educational_activities	2	2
146	3	research	4	3
147	3	research	4	5
148	3	research	4	8
149	3	research	4	5

For peer review only

150	3 research	4	5
151	3 research	4	5
152	3 research	4	4
153	3 research	4	1
154	3 research	4	1
155	3 research	4	1
156	3 research	4	1
157	3 research	4	1
158	3 research	4	1
159	3 research	4	1
160	3 research	4	5
161	3 research	4	5
162	3 research	4	6
163	3 research	4	1
164	3 research	4	1
165	4 learning_seminars	1	9
166	4 learning_seminars	1	9
167	4 preparatory_work	1	1
168	4 preparatory_work	1	1
169	4 external_communication	1	2
170	4 preparatory_work	1	2
171	4 preparatory_work	1	1
172	4 educational_activities	2	35
173	4 educational_activities	2	35
174	4 learning_seminars	1	7
175	4 documentation	1	1
176	4 care_development	2	7
177	4 workplace_meetings	2	70
178	4 external_communication	4	1
179	4 documentation	1	1
180	4 care_development	2	1
181	4 care_development	2	11
182	4 care_development	2	6
183	4 care_development	2	11
184	4 workplace_meetings	2	60
185	4 care_development	2	11
186	4 preparatory_work	4	1
187	4 preparatory_work	4	2
188	4 research	4	6
189	4 external_communication	4	11
190	4 research	4	6
191	4 external_communication	4	6
192	4 learning_seminars	1	9
193	4 learning_seminars	1	8
194	4 educational_activities	2	8
195	4 educational_activities	2	9
196	4 educational_activities	2	9
197	4 educational_activities	2	9
198	4 workplace_meetings	2	70
199	4 documentation	2	1

For peer review only

200	4	care_development	2	70
201	4	care_development	2	8
202	4	care_development	2	8
203	4	care_development	2	2
204	4	care_development	2	2
205	4	research	4	5
206	4	research	4	4
207	4	research	4	4
208	4	research	4	5
209	4	research	4	1
210	4	research	4	1
211	4	research	4	1
212	4	research	4	1
213	4	research	4	1
214	4	research	4	1
215	4	research	4	1
216	4	research	4	1
217	4	research	4	1
218	4	research	4	1
219	4	research	4	2
220	4	research	4	6
221	4	research	4	4
222	4	research	4	1
223	4	research	4	1
224	4	research	4	1
225	4	research	4	1
226	4	research	4	1
227	5	learning_seminars	1	4
228	5	learning_seminars	1	6
229	5	learning_seminars	1	5
230	5	care_development	2	1
231	5	team_development	2	10
232	5	team_development	2	10
233	5	team_development	2	1
234	5	team_development	2	1
235	5	care_development	2	25
236	5	team_development	2	25
237	5	care_development	2	25
238	5	care_development	2	8
239	5	care_development	2	8
240	5	care_development	2	8
241	5	care_development	2	8
242	5	care_development	2	8
243	5	care_development	2	8
244	5	care_development	2	25
245	5	care_development	2	1
246	5	care_development	2	25
247	5	care_development	2	4
248	5	external_communication	4	7
249	5	external_communication	4	2

For peer review only

250	5	external_communication	4	1
251	5	external_communication	4	3
252	5	care_development	2	7
253	5	care_development	2	20
254	5	care_development	2	20
255	5	care_development	2	20
256	5	care_development		
257	5	care_development		
258	5	care_development		
259	5	care_development		
260	5	care_development		
261	5	care_development		
262	5	care_development		
263	5	care_development		
264	5	care_development		
265	5	care_development		
266	5	care_development		
267	5	care_development		
268	5	care_development		
269	5	care_development		
270	5	research	4	3
271	5	research	4	3
272	5	research	4	3
273	5	research	4	1
274	5	research	4	1
275	5	research	4	1
276	5	research	4	1
277	5	research	4	1
278	5	research	4	1
279	5	research	4	1
280	5	research	4	1
281	5	research	4	1
282	5	research	4	4
283	5	research	4	1
284	6	learning_seminars	1	9
285	6	learning_seminars	1	9
286	6	learning_seminars	1	1
287	6	care_development	2	8
288	6	care_development	2	14
289	6	care_development	2	7
290	6	care_development	2	2
291	6	care_development	2	8
292	6	care_development	2	8
293	6	care_development	2	8
294	6	care_development	2	10
295	6	care_development	2	10
296	6	care_development	2	10
297	6	care_development	2	10
298	6	care_development	2	20
299	6	care_development	2	2

For peer review only

300	6 care_development	2	2
301	6 care_development	2	2
302	6 care_development	2	2
303	6 care_development	2	2
304	6 care_development	2	2
305	6 care_development	2	2
306	6 care_development	2	2
307	6 care_development	2	2
308	6 care_development	2	2
309	6 care_development	2	2
310	6 care_development	2	2
311	6 care_development	2	2
312	6 care_development	2	2
313	6 research	4	3
314	6 research	4	2
315	6 research	4	1
316	6 research	4	1
317	6 research	4	1
318	6 research	4	1
319	6 research	4	1
320	6 research	4	1
321	6 research	4	2
322	6 research	4	1
323	6 research	4	3
324	6 research	4	1
325	6 research	4	1
326	6 care_development	2	7
327	7 learning_seminars	1	15
328	7 learning_seminars	1	10
329	7 learning_seminars	1	11
330	7 preparatory_work	1	2
331	7 learning_seminars	1	10
332	7 preparatory_work	1	1
333	7 preparatory_work	1	2
334	7 research	4	5
335	7 internal_communication	1	15
336	7 preparatory_work	1	3
337	7 educational_activities	1	10
338	7 preparatory_work	1	2
339	7 external_communication	4	2
340	7 preparatory_work	1	5
341	7 educational_activities	1	10
342	7 educational_activities	1	10
343	7 external_communication	1	3
344	7 external_communication	1	2
345	7 learning_seminars	1	10
346	7 educational_activities	1	10
347	7 preparatory_work	1	5
348	7 preparatory_work	1	3
349	7 internal_communication	1	3

For peer review only

350	7	research	4	4
351	7	internal_communication	1	3
352	7	internal_communication	1	7
353	7	preparatory_work	1	4
354	7	administration	2	2
355	7	external_communication	4	2
356	7	preparatory_work	1	4
357	7	preparatory_work	1	8
358	7	preparatory_work	1	5
359	7	reporting	2	1
360	7	reporting	2	1
361	7	preparatory_work	1	2
362	7	preparatory_work	1	2
363	7	preparatory_work	1	5
364	7	external_communication	4	1
365	7	preparatory_work	1	3
366	7	internal_communication	1	1
367	7	reporting	2	3
368	7	preparatory_work	1	3
369	7	reporting	2	1
370	7	preparatory_work	1	1
371	7	reporting	2	1
372	7	reporting	2	1
373	7	preparatory_work	1	2
374	7	preparatory_work	1	2
375	7	external_communication	4	1
376	7	reporting	2	1
377	7	preparatory_work	1	3
378	7	preparatory_work	1	2
379	7	preparatory_work	1	3
380	7	preparatory_work	1	2
381	7	research	4	4
382	7	educational_activities	1	2
383	7	preparatory_work	1	3
384	7	external_communication	4	2
385	7	external_communication	4	2
386	7	preparatory_work	1	2
387	7	preparatory_work	1	4
388	7	preparatory_work	1	2
389	7	preparatory_work	1	2
390	7	external_communication	4	1
391	7	internal_communication	1	2
392	7	preparatory_work	1	1
393	7	preparatory_work	1	4
394	7	research	4	1
395	7	preparatory_work	1	3
396	7	preparatory_work	1	5
397	7	preparatory_work	1	3
398	7	preparatory_work	1	4
399	7	reporting	2	2

For peer review only

400	7	preparatory_work	1	2
401	7	preparatory_work	1	2
402	7	preparatory_work	1	2
403	7	preparatory_work	1	1
404	7	preparatory_work	1	1
405	7	preparatory_work	1	12
406	7	research	4	4
407	7	preparatory_work	1	1
408	7	internal_communication	1	3
409	7	preparatory_work	1	3
410	7	reporting	2	2
411	7	internal_communication	1	2
412	7	preparatory_work	1	3
413	7	preparatory_work	1	2
414	7	internal_communication	1	2
415	7	preparatory_work	1	3
416	7	preparatory_work	1	2
417	7	preparatory_work	1	1
418	7	preparatory_work	1	2
419	7	preparatory_work	1	1
420	7	preparatory_work	1	1
421	7	preparatory_work	1	2
422	7	learning_seminars	1	10
423	7	preparatory_work	1	4
424	7	research	4	4
425	7	preparatory_work	1	1
426	7	preparatory_work	1	3
427	7	research	4	4
428	7	preparatory_work	1	1
429	7	preparatory_work	1	3
430	7	preparatory_work	1	10
431	7	educational_activities	1	3
432	7	preparatory_work	1	1
433	7	internal_communication	1	12
434	7	external_communication	4	2
435	7	external_communication	4	2
436	7	preparatory_work	1	3
437	7	preparatory_work	1	3
438	7	preparatory_work	1	1
439	7	preparatory_work	1	2
440	7	preparatory_work	1	4
441	7	research	4	4
442	7	preparatory_work	1	1
443	7	preparatory_work	1	1
444	7	internal_communication	1	3
445	7	preparatory_work	1	2
446	7	preparatory_work	1	1
447	7	learning_seminars	1	10
448	7	preparatory_work	1	3
449	7	educational_activities	1	3

For peer review only

450	7 preparatory_work	1	1
451	7 preparatory_work	1	4
452	7 preparatory_work	1	2
453	7 preparatory_work	1	1
454	7 preparatory_work	1	2
455	7 preparatory_work	1	2
456	7 preparatory_work	1	2
457	7 preparatory_work	1	1
458	7 preparatory_work	1	1
459	7 preparatory_work	1	3
460	7 preparatory_work	1	4
461	7 educational_activities	1	2
462	7 preparatory_work	1	3
463	7 educational_activities	1	2
464	7 preparatory_work	1	2
465	7 preparatory_work	1	3
466	7 preparatory_work	1	1
467	7 preparatory_work	1	3
468	7 learning_seminars	1	10
469	7 preparatory_work	1	1
470	7 educational_activities	1	10
471	7 research	4	3
472	7 preparatory_work	1	4
473	7 preparatory_work	1	5
474	7 preparatory_work	1	5
475	7 preparatory_work	1	4
476	7 preparatory_work	1	1
477	7 administration	2	1
478	7 preparatory_work	1	2
479	7 preparatory_work	1	3
480	7 educational_activities	1	3
481	7 preparatory_work	1	2
482	7 preparatory_work	1	5
483	7 preparatory_work	1	5
484	7 preparatory_work	1	3
485	7 educational_activities	1	10
486	7 educational_activities	1	15
487	7 educational_activities	1	3
488	7 preparatory_work	1	5
489	7 external_communication	4	1
490	7 preparatory_work	1	3
491	7 educational_activities	1	3
492	7 preparatory_work	1	6
493	7 preparatory_work	1	5
494	7 preparatory_work	1	3
495	7 preparatory_work	1	3
496	7 external_communication	4	5
497	7 preparatory_work	1	2
498	7 preparatory_work	1	1
499	7 preparatory_work	1	3

For peer review only

500	7 research	4	5
501	7 preparatory_work	1	2
502	7 preparatory_work	1	1
503	7 preparatory_work	1	6
504	7 preparatory_work	1	3
505	7 preparatory_work	1	1
506	7 preparatory_work	1	5
507	7 preparatory_work	1	3
508	7 internal_communication	1	7
509	7 preparatory_work	1	4
510	7 preparatory_work	1	4
511	7 preparatory_work	1	3
512	7 preparatory_work	1	2
513	7 preparatory_work	1	2
514	7 educational_activities	1	3
515	7 preparatory_work	1	5
516	7 reporting	2	6
517	7 preparatory_work	1	2
518	7 preparatory_work	1	2
519	7 educational_activities	1	7
520	7 preparatory_work	1	2
521	7 preparatory_work	1	2
522	7 preparatory_work	1	1
523	7 research	4	2
524	7 learning_seminars	1	10
525	7 preparatory_work	1	3
526	7 preparatory_work	1	3
527	7 internal_communication	1	1
528	7 preparatory_work	1	2
529	7 preparatory_work	1	3
530	7 preparatory_work	1	3
531	7 preparatory_work	1	3
532	7 research	4	2
533	7 research	4	2
534	7 research	4	1

For peer review only

Time_minutes	Staff_cat
2940	HCP
2940	HCP
2940	HCP
60	managers
960	HCP
9600	HCP
2400	HCP
50000	managers
2400	HCP
1200	HCP
1800	HCP
4800	HCP
300	HCP
4200	HCP
144.55	HCP
201.866666666667	HCP
262.583333333333	HCP
300.833333333333	HCP
143.7	HCP
245.083333333333	HCP
261.583333333333	HCP
45.1	HCP
51.1	HCP
32.9666666666667	HCP
43.6666666666667	HCP
89.5666666666667	HCP
115.233333333333	HCP
193.933333333333	HCP
120.766666666667	HCP
31.0833333333333	managers
33.7166666666667	managers
3360	HCP
4800	HCP
3780	HCP
3780	HCP
2400	managers
600	managers
12000	HCP
10000	HCP
30	managers
3600	HCP
210	HCP
600	managers
960	HCP
1920	HCP
5760	HCP
400	HCP
240	managers
1920	HCP

For peer review only

2430	managers
180	HCP
7440	managers
1080	managers
120	managers
300	HCP
6000	HCP
2500	HCP
1500	HCP
302.1	HCP
232.466666666667	HCP
210.666666666667	HCP
380.333333333333	HCP
268.6	HCP
206.8	HCP
33.4666666666667	HCP
41.5333333333333	HCP
31.5666666666667	HCP
76.8166666666667	HCP
170.266666666667	HCP
126.533333333333	HCP
33.3833333333333	managers
30.5833333333333	managers
1260	HCP
2100	HCP
270	HCP
720	HCP
250	HCP
240	HCP
2940	HCP
630	HCP
450	HCP
3360	HCP
1680	HCP
120	HCP
720	HCP
120	change_agents
50	change_agents
300	HCP
500	change_agents
45	change_agents
60	managers
720	HCP
540	HCP
30	change_agents
840	HCP
1680	HCP
300	HCP
90	change_agents
135	change_agents

For peer review only

90	change_agents
840	HCP
300	change_agents
120	change_agents
8400	HCP
360	HCP
270	HCP
7200	HCP
2100	HCP
2520	HCP
420	HCP
420	HCP
45	change_agents
360	HCP
200	HCP
40	change_agents
420	HCP
240	HCP
120	change_agents
45	change_agents
90	HCP
120	HCP
3780	HCP
240	HCP
30	change_agents
300	change_agents
420	HCP
300	HCP
360	HCP
450	HCP
2100	HCP
240	managers
300	HCP
5400	HCP
2520	HCP
2520	HCP
360	HCP
1680	HCP
1680	HCP
180	managers
2100	HCP
1680	HCP
1680	HCP
240	managers
300	managers
840	HCP
135.2	HCP
249.083333333333	HCP
328.533333333333	HCP
335	HCP

For peer review only

274.916666666667	HCP
225.833333333333	HCP
223	HCP
47.2333333333333	HCP
69.5	HCP
38.4666666666667	HCP
35.5833333333333	HCP
42.5833333333333	HCP
29.9	HCP
44.8666666666667	HCP
191.916666666667	HCP
374.5	HCP
389	HCP
31.1166666666667	managers
34.7833333333333	managers
4320	HCP
4320	HCP
120	HCP
240	HCP
60	HCP
240	HCP
120	HCP
16800	HCP
16800	HCP
3360	HCP
120	HCP
840	HCP
6300	HCP
60	HCP
120	HCP
60	HCP
1320	HCP
720	HCP
660	HCP
10800	HCP
660	HCP
120	HCP
240	HCP
360	managers
4620	HCP
600	HCP
720	HCP
4320	HCP
3840	HCP
3840	HCP
4320	HCP
4320	HCP
4320	HCP
16800	HCP
360	HCP

For peer review only

4200	HCP
3360	HCP
3360	HCP
840	HCP
840	HCP
293.25	HCP
198.4	HCP
229.866666666667	HCP
225.916666666667	HCP
47.2666666666667	HCP
41.2833333333333	HCP
33.0666666666667	HCP
36.2	HCP
41.95	HCP
51.9166666666667	HCP
43.9166666666667	HCP
37.9	HCP
42.0166666666667	HCP
39.05	HCP
45.6	HCP
590.6	HCP
238.333333333333	HCP
24.2333333333333	managers
26.3	managers
37.8	managers
20.65	managers
27.8166666666667	HCP
1920	HCP
2880	HCP
2400	HCP
2400	managers
51000	HCP
25500	HCP
4680	managers
4680	managers
1500	HCP
2100	HCP
1500	HCP
480	HCP
720	HCP
720	HCP
720	HCP
720	HCP
480	HCP
1500	HCP
2400	change_agents
4500	HCP
3360	HCP
2400	HCP
960	HCP

For peer review only

	720	managers
	720	HCP
	105	HCP
	1200	HCP
	1800	HCP
	1200	HCP
		HCP
		HCP
		HCP
		HCP
		HCP
		HCP
		HCP
		HCP
		HCP
		HCP
		HCP
		HCP
		HCP
		HCP
122.9		HCP
171.5		HCP
157.85		HCP
26.2166666666667		HCP
48.8166666666667		HCP
39.6666666666667		HCP
25.9833333333333		HCP
38.9166666666667		HCP
52.9		HCP
26.9833333333333		HCP
32.7		HCP
33.8166666666667		HCP
218.8		HCP
38.4		managers
	4320	HCP
	4320	HCP
	480	HCP
	1260	HCP
	840	HCP
	4200	HCP
	1920	HCP
	960	HCP
	480	HCP
	480	HCP
	600	HCP
	600	HCP
	800	HCP
	600	HCP
	6000	HCP
	3360	HCP

For peer review only

480	HCP
240	HCP
480	HCP
60	HCP
480	HCP
480	HCP
240	HCP
240	HCP
120	HCP
120	HCP
8640	HCP
960	HCP
960	HCP
147	HCP
76.7666666666667	HCP
40.6166666666667	HCP
37.4666666666667	HCP
51.7333333333333	HCP
45.3333333333333	HCP
19.8833333333333	HCP
21.8	HCP
63.3	HCP
33.8166666666667	HCP
121.9	HCP
46.1	managers
28.2833333333333	managers
105	HCP
6300	change_agents
4200	change_agents
4620	change_agents
480	change_agents
4800	change_agents
180	change_agents
180	change_agents
300	change_agents
450	change_agents
135	change_agents
2400	change_agents
60	change_agents
60	change_agents
300	change_agents
1200	managers
1500	managers
180	change_agents
120	change_agents
5400	managers
600	change_agents
150	change_agents
360	change_agents
180	managers

For peer review only

720	change_agents
180	change_agents
840	change_agents
120	change_agents
480	change_agents
80	change_agents
240	change_agents
720	change_agents
2250	change_agents
240	change_agents
240	change_agents
120	managers
360	managers
900	change_agents
120	change_agents
360	change_agents
60	change_agents
360	change_agents
360	change_agents
60	change_agents
120	change_agents
60	change_agents
120	change_agents
120	change_agents
120	change_agents
60	change_agents
120	change_agents
360	change_agents
240	change_agents
540	change_agents
120	change_agents
480	change_agents
240	change_agents
360	change_agents
960	change_agents
960	change_agents
120	change_agents
480	change_agents
180	change_agents
180	change_agents
30	change_agents
180	change_agents
15	change_agents
240	change_agents
240	change_agents
360	change_agents
600	change_agents
360	change_agents
480	managers
60	change_agents

For peer review only

60	managers
30	managers
60	change_agents
150	change_agents
150	change_agents
360	change_agents
360	change_agents
90	change_agents
180	change_agents
180	change_agents
60	change_agents
120	change_agents
270	change_agents
120	change_agents
60	change_agents
180	change_agents
240	change_agents
30	change_agents
240	change_agents
60	change_agents
300	change_agents
120	change_agents
4800	change_agents
240	managers
240	change_agents
180	change_agents
180	change_agents
360	change_agents
90	change_agents
180	change_agents
600	change_agents
540	change_agents
180	change_agents
360	change_agents
960	change_agents
960	change_agents
180	change_agents
180	change_agents
120	change_agents
180	change_agents
720	change_agents
360	change_agents
60	change_agents
60	change_agents
360	change_agents
480	change_agents
240	change_agents
4800	change_agents
360	change_agents
1440	change_agents

For peer review only

60	change_agents
960	change_agents
60	change_agents
60	change_agents
240	change_agents
60	change_agents
480	change_agents
90	change_agents
60	change_agents
180	change_agents
480	change_agents
960	managers
1080	change_agents
960	managers
360	change_agents
180	change_agents
60	change_agents
360	change_agents
4800	change_agents
180	change_agents
4200	change_agents
540	change_agents
480	change_agents
600	change_agents
600	change_agents
480	change_agents
360	change_agents
240	change_agents
180	change_agents
540	change_agents
900	change_agents
240	change_agents
600	change_agents
600	change_agents
300	change_agents
2100	change_agents
2700	change_agents
900	managers
300	change_agents
60	change_agents
540	change_agents
270	change_agents
720	change_agents
300	change_agents
720	change_agents
810	change_agents
3000	change_agents
480	change_agents
60	change_agents
540	change_agents

For peer review only

2250	change_agents
120	change_agents
150	change_agents
1620	managers
180	managers
120	change_agents
450	change_agents
720	change_agents
630	change_agents
120	change_agents
120	change_agents
360	change_agents
180	change_agents
120	change_agents
540	managers
750	change_agents
360	managers
240	change_agents
600	change_agents
840	managers
30	change_agents
120	change_agents
120	change_agents
120	change_agents
4200	managers
360	change_agents
720	change_agents
120	change_agents
240	change_agents
360	change_agents
270	change_agents
540	change_agents
198.866666666667	change_agents
104.966666666667	change_agents
35.95	change_agents

For peer review only

Geriatric rehabilitation

Unit 1

Year	Month	Length of stay, mean
2016	January	31.5
2016	February	28.6
2016	March	22.0
2016	April	22.8
2016	May	31.9
2016	June	26.1
2016	July	39.3
2016	August	24.3
2016	September	34.6
2016	October	30.0
2016	November	34.0
2016	December	28.1
2017	January	26.2
2017	February	25.1
2017	March	23.3
2017	April	25.1
2017	May	26.9
2017	June	29.5
2017	July	32.5
2017	August	28.2
2017	September	22.2
2017	October	27.7
2017	November	20.3
2017	December	29.2
2018	January	29.3
2018	February	19.3
2018	March	32.6
2018	April	28.1
2018	May	29.8
2018	June	29.7
2018	July	24.4
2018	August	39.4
2018	September	22.5
2018	October	28.0
2018	November	22.7
2018	December	19.9
2019	January	29.5
2019	February	18.9
2019	March	23.6
2019	April	37.2
2019	May	28.5
2019	June	22.8
2019	July	19.0
2019	August	21.1
2019	September	26.0
2019	October	25.0
2019	November	26.9

For peer review only

Unit 2

Year	Number of discharges
2016	691
2017	706
2018	675
2019	710

Psychiatrics

Unit 1 and unit 2, combined (aggregated data)

Year	Length of stay, mean ±SD	Time to readmissions, mean ±SD
2016	15.5 ±16.4	219.3 ±277.6
2017	15.2 ±15.0	160.2 ±180.8
2018	14.6 ±23.7	79.7 ±88.3
2019	13.9 ±14.1	28.1 ±29.8

For peer review only

For peer review only

±

For peer review only

Online-Only Supplements

An analysis on personnel costs and working time for implementing a more person-centred care approach: A case study with embedded units in a Swedish region

Authors

Hanna Gyllensten[1,2] Associate professor

Malin Tistad[3,4] Associate professor

Helena Fridberg[3] PhD

Lars Wallin[1,2,3] Professor

Author affiliations

[1] Institute of Health and Care Sciences, University of Gothenburg, Box 457, SE-405 30 Gothenburg, Sweden.

[2] University of Gothenburg Centre for Person-Centred Care (GPCC), Sahlgrenska Academy, University of Gothenburg, Sweden.

[3] School of Health and Welfare, Dalarna University, Falun, Sweden.

[4] Department of Neurobiology, Care Sciences and Society, Karolinska Institutet, Stockholm, Sweden.

Content

eTable 1: Resource use reported in logbooks by type of activity and organisational units. 2

eFigure 1: Length of stay at the geriatric unit (unit 1) and trend over time. 3

eFigure 2: Length of stay at the psychiatric units (combined) and trend over time. 4

eFigure 3: Time to readmission at the psychiatric units (combined) and trend over time. 5

For peer review only - http://bmjopen.bmj.com/site/about/guidelines.xhtml

eTable 1: Resource use reported in logbooks by type of activity and organisational units.

Activities by cost categories[a]	Organisational unit							
	Geriatric unit 1 working days[b]	Geriatric unit 2 working days[b]	Nephrology unit working days[b]	Primary care unit working days[b]	Psychiatric unit 1 working days[b]	Psychiatric unit 2 working days[b]	DD working days[b]	Total working days[b]
Implementation strategies								
Documentation	0	0	0	1	0	0	0	1
Educational activities	0	0	0	0	0	0	46	46
External communication	0	0	0	0	0	0	1	1
Internal communication	0	0	0	0	0	0	8	8
Learning seminars	18	25	57	42	15	19	92	268
Preparatory work	1	70	11	2	0	0	88	171
Workplace meetings	0	0	1	0	0	0	0	1
Service delivery								
Administration	0	0	0	0	0	0	2	2
Care development	113	41	32	35	53	74	0	348
Documentation	0	0	0	1	0	0	0	1
Educational activities	26	4	37	105	0	0	0	172
IT support	0	0	0	0	0	0	0	0
Reporting	0	0	0	0	0	0	4	4
Team development	23	22	5	0	183	0	0	233
Workplace meetings	0	0	0	71	0	0	0	71
Research/development								
External communication	0	0	11	11	10	0	15	48
Preparatory work	0	0	0	1	0	0	0	1
Research	5	4	9	7	2	2	13	42
Total	185	167	162	275	263	95	267	1414

[a] Developed from previously reported cost categories in implementation programmes: 1) costs for executing implementation strategies, 2) excess costs for service delivery as implementation/service changes, 3) opportunity costs to providers and patients, and 4) research/development costs (35). No account was taken for changes in costs for direct patient care (category 3).

[b] Assuming 8-hour working days.

Abbreviations: DD = Department for development.

For peer review only

eFigure 1: Length of stay at the geriatric unit (unit 1) and trend over time.

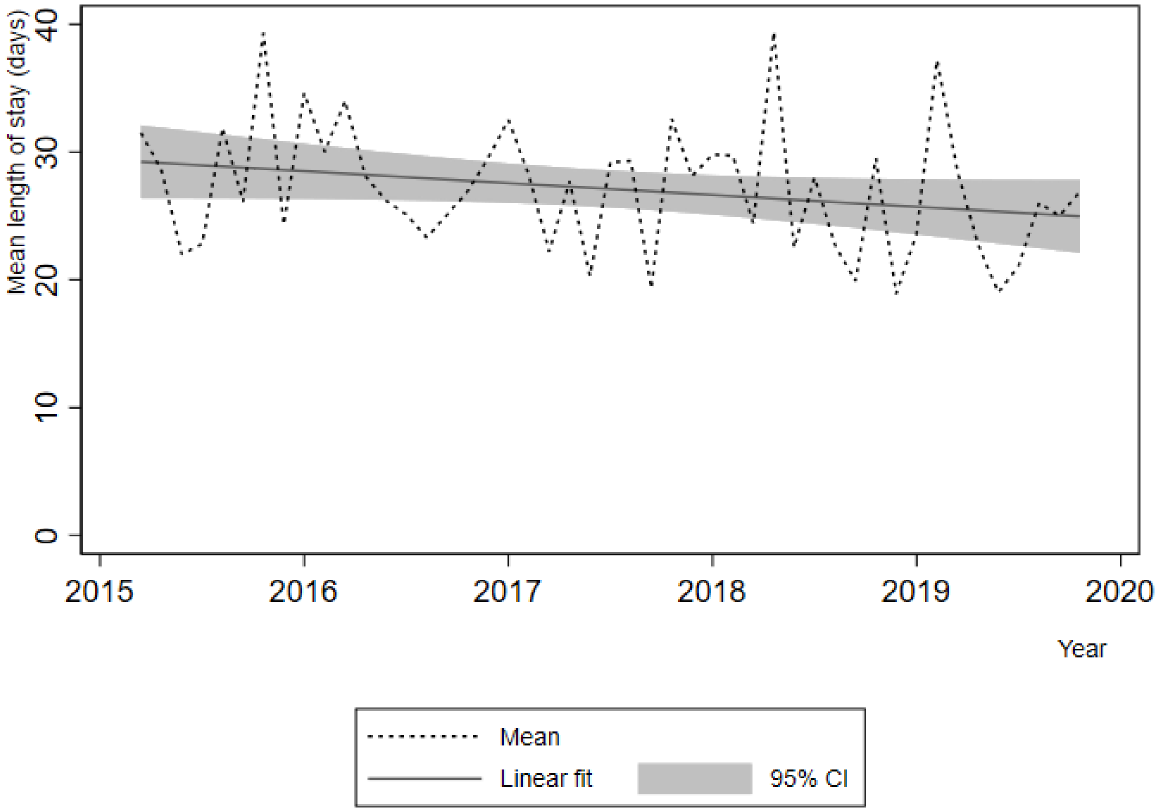

For peer review only - http://bmjopen.bmj.com/site/about/guidelines.xhtml

eFigure 2: Length of stay at the psychiatric units (combined) and trend over time.

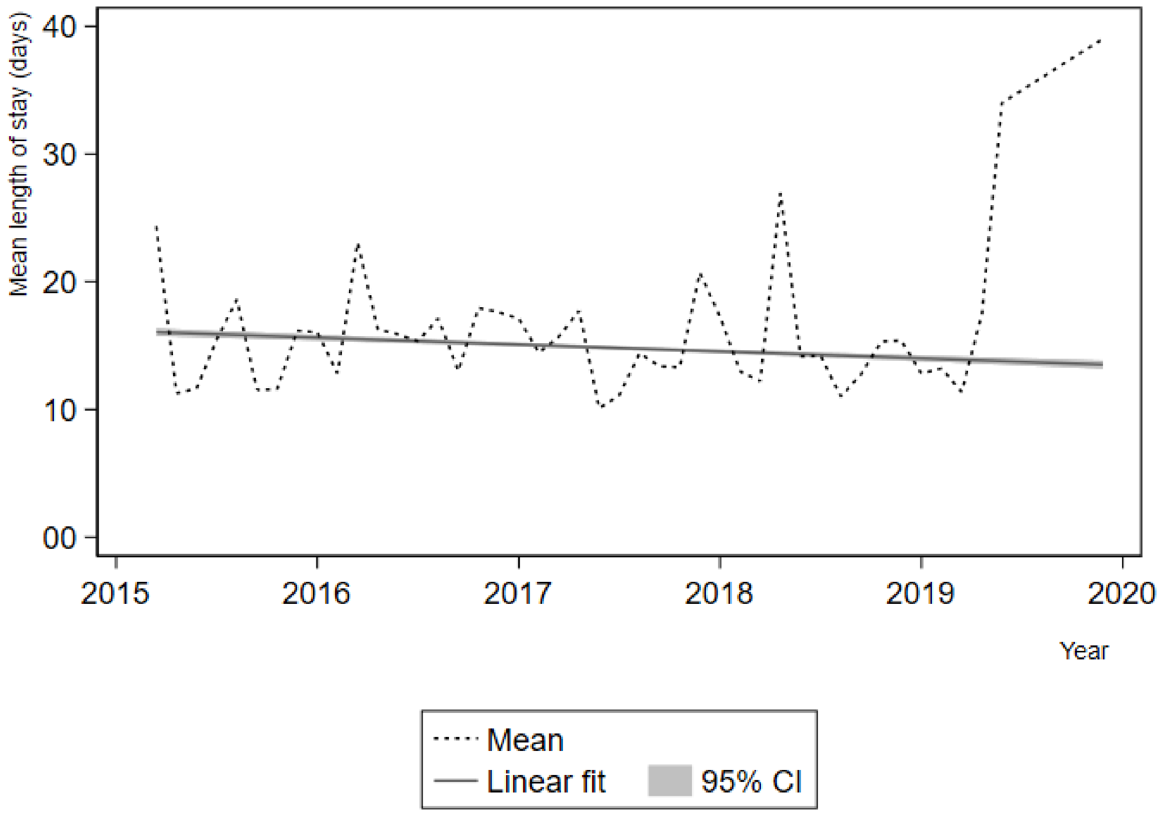

Observe that the 95% confidence interval is not visible (overlaid by the linear equation line) due to large sample size.

For peer review only - http://bmjopen.bmj.com/site/about/guidelines.xhtml

eFigure 3: Time to readmission at the psychiatric units (combined) and trend over time.

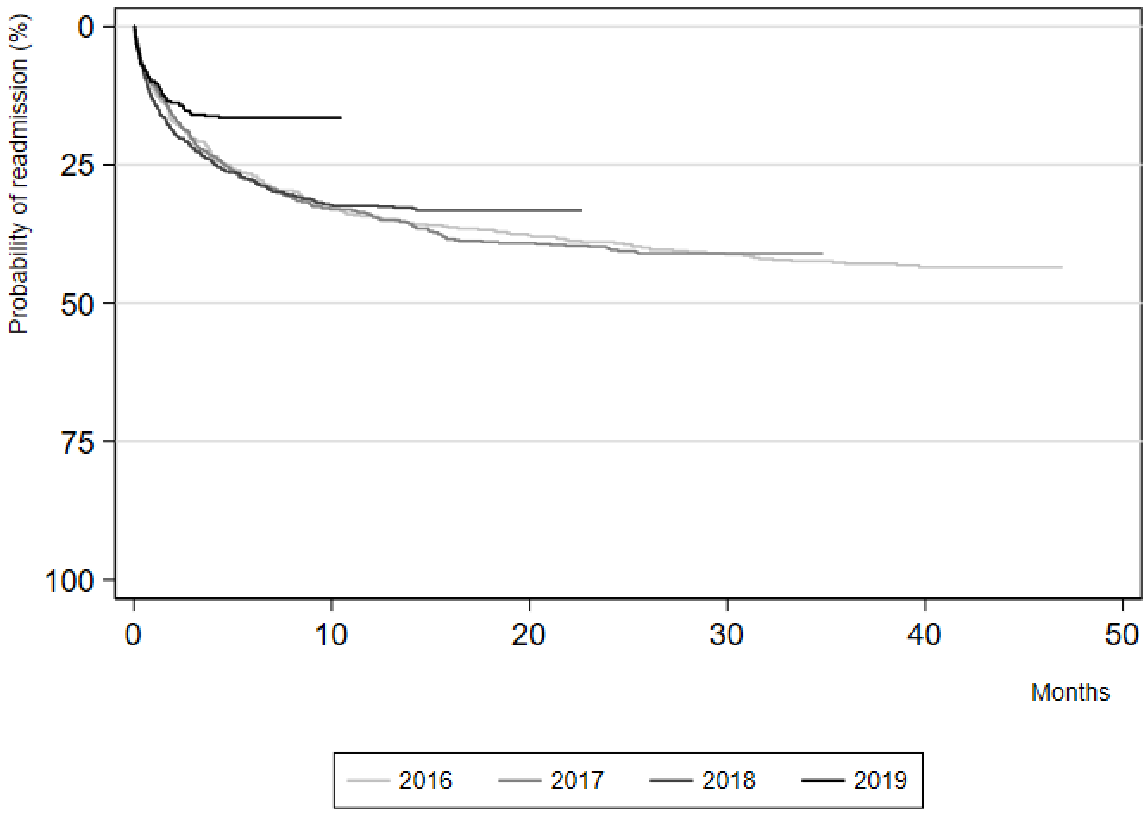

For peer review only

For peer review only - http://bmjopen.bmj.com/site/about/guidelines.xhtml

CHEERS 2022 Checklist

Topic	No.	Item	Location where item is reported
**Title**			
	1	Identify the study as an economic evaluation and specify the interventions being compared.	Page 1
**Abstract**			
	2	Provide a structured summary that highlights context, key methods, results, and alternative analyses.	Page 2
**Introduction**			
**Background and objectives**	3	Give the context for the study, the study question, and its practical relevance for decision making in policy or practice.	Page 4
**Methods**			
**Health economic analysis plan**	4	Indicate whether a health economic analysis plan was developed and where available.	Supplement S1
**Study population**	5	Describe characteristics of the study population (such as age range, demographics, socioeconomic, or clinical characteristics).	N/A (staff only)
**Setting and location**	6	Provide relevant contextual information that may influence findings.	Pages 5-6
**Comparators**	7	Describe the interventions or strategies being compared and why chosen.	Page 6
**Perspective**	8	State the perspective(s) adopted by the study and why chosen.	Pages 9-10
**Time horizon**	9	State the time horizon for the study and why appropriate.	N/A
**Discount rate**	10	Report the discount rate(s) and reason chosen.	N/A
**Selection of outcomes**	11	Describe what outcomes were used as the measure(s) of benefit(s) and harm(s).	N/A
**Measurement of outcomes**	12	Describe how outcomes used to capture benefit(s) and harm(s) were measured.	N/A

For peer review only - http://bmjopen.bmj.com/site/about/guidelines.xhtml

Topic	No.	Item	Location where item is reported
**Valuation of outcomes**	13	Describe the population and methods used to measure and value outcomes.	N/A
**Measurement and valuation of resources and costs**	14	Describe how costs were valued.	Pages 9-10
**Currency, price date, and conversion**	15	Report the dates of the estimated resource quantities and unit costs, plus the currency and year of conversion.	Page 10
**Rationale and description of model**	16	If modelling is used, describe in detail and why used. Report if the model is publicly available and where it can be accessed.	N/A
**Analytics and assumptions**	17	Describe any methods for analysing or statistically transforming data, any extrapolation methods, and approaches for validating any model used.	Page 10
**Characterising heterogeneity**	18	Describe any methods used for estimating how the results of the study vary for subgroups.	N/A
**Characterising distributional effects**	19	Describe how impacts are distributed across different individuals or adjustments made to reflect priority populations.	N/A
**Characterising uncertainty**	20	Describe methods to characterise any sources of uncertainty in the analysis.	N/A
**Approach to engagement with patients and others affected by the study**	21	Describe any approaches to engage patients or service recipients, the general public, communities, or stakeholders (such as clinicians or payers) in the design of the study.	Pages 8 and 16 (staff)<br>No patient/public
**Results**			
**Study parameters**	22	Report all analytic inputs (such as values, ranges, references) including uncertainty or distributional assumptions.	Table 3
**Summary of main results**	23	Report the mean values for the main categories of costs and outcomes of interest and summarise them in the most appropriate overall measure.	Table 2
**Effect of uncertainty**	24	Describe how uncertainty about analytic judgments, inputs, or projections affect findings. Report the effect of choice of discount rate and time horizon, if applicable.	N/A

For peer review only - http://bmjopen.bmj.com/site/about/guidelines.xhtml

Topic	No.	Item	Location where item is reported
**Effect of engagement with patients and others affected by the study**	25	Report on any difference patient/service recipient, general public, community, or stakeholder involvement made to the approach or findings of the study	N/A
**Discussion**			
**Study findings, limitations, generalisability, and current knowledge**	26	Report key findings, limitations, ethical or equity considerations not captured, and how these could affect patients, policy, or practice.	Page 16
**Other relevant information**			
**Source of funding**	27	Describe how the study was funded and any role of the funder in the identification, design, conduct, and reporting of the analysis	Page 21
**Conflicts of interest**	28	Report authors conflicts of interest according to journal or International Committee of Medical Journal Editors requirements.	Page 21

From: Husereau D, Drummond M, Augustovski F, et al. Consolidated Health Economic Evaluation Reporting Standards 2022 (CHEERS 2022) Explanation and Elaboration: A Report of the ISPOR CHEERS II Good Practices Task Force. Value Health 2022;25. doi:10.1016/j.jval.2021.10.008

review only

For peer review only - http://bmjopen.bmj.com/site/about/guidelines.xhtml
